# Data for Genetic Analysis Workshop 18: human whole genome sequence, blood pressure, and simulated phenotypes in extended pedigrees

**DOI:** 10.1186/1753-6561-8-S1-S2

**Published:** 2014-06-17

**Authors:** Laura Almasy, Thomas D Dyer, Juan M Peralta, Goo Jun, Andrew R Wood, Christian Fuchsberger, Marcio A Almeida, Jack W Kent, Sharon Fowler, Tom W Blackwell, Sobha Puppala, Satish Kumar, Joanne E Curran, Donna Lehman, Goncalo Abecasis, Ravindranath Duggirala, John Blangero, The T2D-GENES Consortium

**Affiliations:** 1 Department of Genetics, Texas Biomedical Research Institute, San Antonio, Texas, 78227, USA; 2 Department of Biostatistics, Center for Statistical Genetics, University of Michigan, Ann Arbor, Michigan, 48109, USA; 3 Genetics of Complex Traits, Peninsula College of Medicine and Dentistry, University of Exeter, Exeter, UK; 4 Division of Clinical Epidemiology, Department of Medicine, University of San Antonio Health Science Center at San Antonio, San Antonio, Texas, 78229, USA

## Abstract

Genetic Analysis Workshop 18 (GAW18) focused on identification of genes and functional variants that influence complex phenotypes in human sequence data. Data for the workshop were donated by the T2D-GENES Consortium and included whole genome sequences for odd-numbered autosomes in 464 key individuals selected from 20 Mexican American families, a dense set of single-nucleotide polymorphisms in 959 individuals in these families, and longitudinal data on systolic and diastolic blood pressure measured at 1-4 examinations over a period of 20 years. Simulated phenotypes were generated based on the real sequence data and pedigree structures. In the design of the simulation model, gene expression measures from the San Antonio Family Heart Study (not distributed as part of the GAW18 data) were used to identify genes whose mRNA levels were correlated with blood pressure. Observed variants within these genes were designated as functional in the GAW18 simulation if they were nonsynonymous and predicted to have deleterious effects on protein function or if they were noncoding and associated with mRNA levels. Two simulated longitudinal phenotypes were modeled to have the same trait distributions as the real systolic and diastolic blood pressure data, with effects of age, sex, and medication use, including a genotype-medication interaction. For each phenotype, more than 1000 sequence variants in more than 200 genes present on the odd-numbered autosomes individually explained less than 0.01-2.78% of phenotypic variance. Cumulatively, variants in the most influential gene explained 7.79% of trait variance. An additional simulated phenotype, Q1, was designed to be correlated among family members but to not be associated with any sequence variants. Two hundred replicates of the phenotypes were simulated, with each including data for 849 individuals.

## Background

The Genetic Analysis Workshop 18 (GAW18) data set consisted of whole genome sequence data in a pedigree-based sample, longitudinal phenotype data for hypertension and related traits, and 200 replicates of simulated longitudinal phenotype data that used the real genotypes, pedigree structures, and trait distributions. Genetic data for GAW18 included aligned and called whole genome sequences for odd-numbered autosomes, sequence calls cleaned of mendelian errors for the sequenced individuals and imputed genotypes for their family members, and the dense single-nucleotide polymorphism (SNP) data used for the imputing of sequence in family members. In addition, genotype dosages were provided for each called SNP in terms of number of minor alleles carried (0, 1, or 2) with a weighted average used for imputed genotypes that could not be determined unambiguously. Real phenotype data included sex, age, year of examination, systolic and diastolic blood pressure, use of antihypertensive medications, and tobacco smoking at up to four time points.

## Methods

### T2D-GENES study

The Type 2 Diabetes Genetic Exploration by Next-Generation Sequencing in Ethnic Samples (T2D-GENES) Consortium is a collaborative international effort to identify genes influencing susceptibility to type 2 diabetes. The GAW18 data set was drawn from T2D-GENES Project 2, a complex pedigree-based study designed to identify low-frequency or rare variants that influence susceptibility to type 2 diabetes using information from whole genome sequencing (WGS) of 1043 individuals from 20 Mexican American pedigrees enriched for type 2 diabetes from San Antonio, Texas. These family data were obtained from two studies: the San Antonio Family Heart Study (SAFHS) and the San Antonio Family Diabetes/Gallbladder Study (SAFDGS), which are together referred to as the San Antonio Family Studies (SAFS). The T2D-GENES Consortium sequenced approximately 600 individuals chosen for their value in imputing sequence information in about 450 additional family members. This is possible because all individuals in the sample were previously assessed for a high-density SNP framework. WGS is being performed commercially at Complete Genomics Inc (CGI), and the GAW18 data set was based on the sequence data for the first 483 T2D-GENES samples.

The T2D-GENES Project 2 family data were drawn from two San Antonio-based family studies: SAFHS and SAFDGS. The SAFHS began in 1991 with 40- to 60-year-old low-income Mexican Americans, selected at random without regard to presence or absence of disease, who were almost exclusively from Mexican American census tracts in San Antonio, Texas [1]. All first-, second-, and third-degree relatives of the proband and of the proband's spouse, age 16 years or older, were eligible to participate in the study. Participants were recalled for up to four examinations over an approximately 20-year period. The SAFDGS also began in 1991 as the San Antonio Family Diabetes Study [2] and recruited low-income Mexican Americans with type 2 diabetes identified in an earlier epidemiologic survey, the San Antonio Heart Study. All first-, second-, and third-degree relatives, age 18 or older, were invited to participate in the study. Participants were recalled twice for a total of up to three examinations per person. The second recall began the gallbladder component of the study, recruited new family members, and added 8 newly recruited families [3,4].

From the SAFS families, 20 large pedigrees, consisting of 1043 individuals, were selected for T2D-GENES Project 2 by focusing on large lineages to maximize the number of potential copies of founder alleles and to get an optimal ratio of sequencing efficiency and number of individuals with type 2 diabetes. These pedigrees average 52 individuals, with a maximum pedigree size of 87 individuals. To select individuals for WGS, the program ExomePicks (http://genome.sph.umich.edu/wiki/ExomePicks) was used to choose approximately 600 individuals. ExomePicks is designed to select an optimal subset of individuals to sequence to infer WGS calls in remaining family members using a framework of previously typed SNPs to identify regions of identity-by-descent sharing between sequenced and unsequenced individuals. The sequences for the remaining family members were obtained using family-based imputation around a previously assessed high-density SNP framework.

The data set for GAW18 included 20 pedigrees with 21-76 individuals with blood pressure measurements at one or more exams. These families included two examined pairs of monozygotic twins in two different families. The maximum set of genetically unrelated individuals with phenotype data that could be extracted from these pedigrees consisted of 157 individuals.

### Whole genome sequence data

GAW18 used an early version of the T2D-GENES "freeze 1" data set, prepared in early 2012. WGS data used for GAW18 came from 483 individuals sequenced by CGI at an average 60× coverage. Of these, 19 samples failed to meet SNP quality control criteria, such as number of SNPs called, fractions and ratio of homozygous and heterozygous sites, and fraction of novel SNPs, leaving sequence data for 464 individuals. Pedigree information was verified by estimated kinship coefficients, principal components analysis (PCA), and number of mendelian errors between parent and offspring samples. A novel multisample SNP filtering pipeline was used to collect quality measures across all samples, including allele balance, strand bias, fraction of bases with low quality, and fraction of mendelian errors. Support vector machine (SVM) classifiers were used to filter out low-quality SNPs.

In the 483 individuals, 26.8M SNPs were identified, and after eliminating the 19 outlier individuals, 24M SNPs passed SVM and insertion-deletion (INDEL) proximity filters. More than 69% of the quality-controlled SNPs were not in dbSNP 129, and the overall transition to transversion ratio was 2.18, whereas SNPs in dbSNP have a transition to transversion ratio of 2.19. More than 51% of SNPs had minor allele frequency (MAF) less than 1%. The aligned, called sequence data for the 464 individuals who passed quality control checks were provided in variant call format (vcf) files for GAW18. Information fields provided in the vcf files included number of samples with fully called data, allele frequency, dbSNP membership, dbSNP rs identifier, strand bias Pearson's correlation, strand bias *z*-score, cycle bias Pearson's correlation, cycle bias *z*-score, cycle-strand Pearson's correlation, base-quality inflation *z*-score, ratio of base-quality inflation, alternate allele quality *z*-score, alternate allele inflation score, and fraction of bases with map quality of 0, less than 10, less than 20, and less than 30.

A novel population-based imputation approach, prephasing imputation, was used to impute WGS data for 961 individuals in the 20 large pedigrees based on an existing framework of dense SNPs designed for genome-wide association studies (GWAS) [5]. This approach works in two steps. First, haplotypes are estimated for each individual for the GWAS data (prephasing). Second, the estimated haplotypes are used directly for imputation of sequence variants.

MaCH was used for prephasing the GWAS data [6]. This haplotyping approach proceeds through a series of iterative steps. In each step a new pair of haplotypes is sampled for each individual as an imperfect mosaic of the estimated haplotypes ("templates") for other individuals in the data set. After a number of iterations, "best-guess" haplotypes are constructed for each individual by combining information across the sampled haplotype configurations. For the present data set, 20 iterations and 400 templates were used. After GWAS genotypes are phased, each haplotype can be imputed separately if it is assumed that the GWAS haplotypes are conditionally independent, given a reference panel. The reference panel provides template haplotypes for the imputation model, and marginal probabilities for the untyped alleles in each GWAS haplotype are estimated by means of standard hidden Markov model (HMM) calculations (the "forward-backward" algorithm).

As an initial approximating procedure, the preliminary imputation ignored family structure. To eliminate errors and improve the overall quality of imputation, we then proceeded to identify all obligate mendelian errors using the computer program SimWalk2 [7]. This analysis used all available pedigree information. Using a mendelian probability model, likely errors were identified and iteratively blanked until each marker configuration could pass a likelihood calculation test (ie, produce a nonzero likelihood), indicating the absence of mendelian inconsistencies. Following this error detection phase, we then reimputed the blanked genotypes, now using information on the genotypes of surrounding family members. The program MERLIN [8] was employed for this, using the general approach of Burdick et al [9]. Because of the high computational burden associated with this procedure in large pedigrees, for each individual with missing genotypes, we formed a trimmed locally optimal pedigree containing as much haplotype transmission data as could be reasonably placed in memory to optimize the speed of computation while maximizing genetic information. For this procedure we used a maximum of 16 bits of pedigree extension per individual. For each missing genotype the probabilities of each possible genotype were calculated in the context of the local haplotypes. The resulting probabilities were then used to generate an appropriately weighted gene dosage variable. These gene dosage measures were provided for each sequence variant. However, when imputed genotype calls were ambiguous, blanks were retained in the imputed genotype file.

Genotype calls cleaned of mendelian errors were provided for 959 individuals (464 directly sequenced and the rest imputed) for 8,348,674 locations in the genome. These sequence data were for odd-numbered autosomes only and did not include structural variants. GWAS data for 472,049 SNPs on odd-numbered autosomes were provided for these 959 family members. These data were obtained using different versions of the Illumina Infinium Beadchips: HumanHap550v3, supplemented with HumanExon510Sv1; Human660W-Quadv1; Human1Mv1; and Human1M-Duov3. The raw GWAS genotype data obtained were processed using standard quality control procedures, and SNP genotypes cleaned of mendelian errors were provided for odd-numbered autosomes for use by GAW18 participants who wished to work on methods for imputing sequence data through the pedigrees. Finally, for participants who wished to analyze data using the full pedigree structure but who were not interested in methodological issues related to cleaning or imputing sequence data, a file of called variant dosages was provided with the entry for each variant being the estimated number of minor alleles carried, obtained either from the direct sequence or by imputation.

### Phenotype data

Participants in both SAFHS and SAFDGS were followed in a mixed longitudinal fashion. Blood pressure measurements were taken during one or more study exams from 932 SAFS participants in the 20 T2D-GENES sequencing families (Table 1). Of these individuals, 246 had one blood pressure measure, 183 had two, 309 had three, and 194 had four. For each examination, blood pressure was measured three times after a five-minute rest with a Random Zero sphygmomanometer. The numbers provided were the averages of the second and third readings. The phenotype data provided consisted of sex, age at examination, year of examination, systolic blood pressure (SBP), diastolic blood pressure (DBP), current use of antihypertensive medications, hypertension diagnosis, and current tobacco smoking at the time of each exam for up to four time points. Hypertension was defined as SBP > 140, DBP > 90, or antihypertensive medication use at that examination. Note that because diagnosis for each time point was defined using the data only at that particular examination, affected individuals may appear to revert and become unaffected at a later exam if their SBP or DBP decreased and they were unmedicated.

**Table 1:** SAFS phenotype data

	Exam 1	Exam 2	Exam 3	Exam 4
*N*^a^	855	605	622	233
Year of exam	1981^b^-1996	1997-2000	1998-2006	2009-2011
Mean age at exam (range)	39.6 (16-94)	42.9 (17-97)	46.3 (18-95)	50.9 (30-81)
Mean SBP (range)	122 (80-216)	125 (90-211)	125 (76-220)	128 (93-233)
Mean DBP (range)	71 (40-123)	72 (43-115)	71 (32-108)	78 (46-126)
Antihypertensive medication use (%)	9.79	18.97	28.75	43.29
Hypertension (%)	18.13	28.38	34.77	51.93
Current smokers (%)	22.90	18.25	20.00	11.16

a. Number with blood pressure measurements.

b. Although both SAFHS and SAFDGS began in 1991, data from an earlier study were included at exam 1 for one participant.

### Simulated phenotypes

Simulated phenotypes were modeled after the real data with SBP and DBP distributions and frequencies of hypertension, medication use, and tobacco smoking taken from the SAFS T2D-GENES data. An additional simulated quantitative trait not present in the real data set, Q1, was also added. The simulation model used the real pedigrees and the cleaned imputed sequence data for each individual and was constructed to maintain the heritabilities of SBP and DBP and the observed correlations between them. Note, however, that upper and lower bounds were not placed on simulated SBP or DBP, nor was simulated SBP required to be greater than simulated DBP. This resulted in a handful of individuals with biologically implausible simulated trait values.

The sample for the simulated data set was the individuals who had both phenotype data and imputed sequence data in the real data set: 849 individuals. Two hundred replicates of simulated phenotype data were generated. All individuals had simulated phenotype data at three time points with no missing data, with the exception of Q1, which was provided only for the first exam. Each individual's sex was taken from the real data set, and ages from the real data set were used when possible. For individuals not examined at all time points in the real data set, missing ages at exam were filled in by adding or subtracting 3.9 years between exams 1 and 2 and/or 6.9 years between exams 1 and 3. Because age and sex were based on the real T2D-GENES data, they did not vary across replicates of the simulated data. SBP, DBP, hypertension diagnosis, medication use, and tobacco smoking were generated anew for each simulation replicate.

### Simulation model

The GAW18 simulation model was extensively informed by patterns in the real SAFS blood pressure data. Measures of gene expression in lymphocytes were available from the first SAFHS exam [10] and were used to select "functional" genes for the GAW18 phenotype simulation. Genetic correlations between measures of mRNA expression and SBP and DBP were estimated. Genes on odd-numbered chromosomes whose expression levels were both phenotypically and genetically correlated with either SBP or DBP at a *p *value of 0.05 were selected, and their expression levels were tested for association (at *p *< 0.05) against the sequence data to identify cis-regulatory variants within 5kb upstream and downstream of the gene. For each gene we then performed a stepwise regression to identify the conditional effects of individual SNPs, again requiring a *p *< 0.05 for a SNP to pass this conditional association test. The estimated conditional effect size for each SNP was the basis for its phenotypic effect on the simulated phenotypes. In addition, PolyPhen was used to identify potentially deleterious coding variants in these same genes.

Direction and magnitude of effect for each SNP were determined using the observed correlation between mRNA and SBP/DBP for regulatory variants. For coding variants we assumed that deleterious variants would decrease function. If mRNA was positively correlated with SBP or DBP, then deleterious coding variants decreased mean phenotype levels; and, conversely, if mRNA was negatively correlated with SBP/DBP, then deleterious coding variants increased blood pressures. The magnitude of the effect size for coding variants was primarily a multiplicative function of the PolyPhen-2 score (PP2S, ranging from 0 to 1) and the observed genetic correlation (*ρ_g_*) between transcript and SBP/DBP measure. Specifically, for a given phenotype, effect size was determined by [(percentile of ranked PP2S) × (PP2S^2^) × (*ρ_g_*) × *k *× *l*], where *k *is an overall constant and *l *is a gene-specific constant. There were 1243 variants in 245 genes that influenced variation in simulated DBP and 1040 variants in 205 genes that influenced variation in simulated SBP. Table 2 lists the top 15 genes with the largest effects on each of simulated DBP and SBP, and Table 3 lists the top 55 individual variants, all of which are nonsynonymous coding variants that account for at least a tenth of a percent of variance in simulated DBP, SBP, or both. Additional File 1 lists all 1458 functional variants. Individual variants accounted for less than 0.001% to as much as 2.78% of the phenotypic variance in DBP and SBP. If the effects of all variants in a gene were combined, each gene accounted for less than 0.001% to as much as 7.79% of phenotypic variance.

**Table 2:** Top 15 genes influencing simulated SBP and DBP

Gene	Chromosome	Position of first functional SNP (bp)	Total variance explained (%)	Variance explained by largest functional variant (%)	Number of functional variants
**DBP**					
*MAP4*	3	47912898	6.48	2.29	15
*TNN*	1	174996637	4.08	1.98	18
*NRF1*	7	129252980	2.65	1.08	14
*LEPR*	1	65912722	2.50	2.19	8
*FLT3*	13	28567172	1.22	1.01	10
*ZFP37*	9	115803080	0.92	0.49	9
*CGN*	1	151491026	0.83	0.60	16
*MTRR*	5	7870973	0.36	0.10	11
*SLC35E2*	1	1658093	0.36	0.14	7
*ZNF443*	19	12513424	0.34	0.26	13
*RAI1*	17	17498492	0.32	0.30	7
*PTTG1IP*	21	46266768	0.28	0.16	7
*CABP2*	11	67288594	0.21	0.21	1
*ZNF544*	19	58740339	0.21	0.18	9
*REPIN1*	7	150041836	0.20	0.10	6
**SBP**					
*MAP4*	3	47912898	7.79	2.79	15
*NRF1*	7	129252980	4.67	1.91	14
*TNN*	1	174996637	3.87	1.89	16
*LEPR*	1	65912722	2.23	2.06	8
*FLT3*	13	28567172	0.97	0.82	8
*GTF2IRD1*	7	73822336	0.36	0.10	10
*FLNB*	3	58089761	0.29	0.27	8
*ZNF443*	19	12513424	0.22	0.17	11
*GSN*	9	123969834	0.21	0.09	13
*CABP2*	11	67288594	0.17	0.17	1
*LRP8*	1	53712727	0.17	0.17	2
*PSMD5*	9	123575167	0.16	0.13	6
*GAB2*	11	77935643	0.14	0.08	4
*ABTB1*	3	127394820	0.13	0.10	2
*KRTAP11-1*	21	3225351	0.13	0.13	1

**Table 3:** Top 55 variants influencing simulated SBP and DBP, in decreasing order of effect size

Gene	Chromosome	Position	MAF	Beta^a ^DBP	DBP variance explained (%)	Beta^a ^SBP	SBP variance explained (%)
*MAP4*	3	48040283	0.0318	−6.22	2.29	−9.91	2.78
*LEPR*	1	66075952	0.1567	2.76	2.19	3.87	2.06
*MAP4*	3	47957996	0.0301	−4.64	1.22	−7.39	1.49
*MAP4*	3	47956424	0.3777	−1.50	1.17	−2.38	1.43
*MAP4*	3	48040284	0.0131	−6.95	0.91	−11.07	1.11
*FLT3*	13	28624294	0.4167	1.38	1.01	1.79	0.81
*MAP4*	3	47913455	0.0049	−5.46	0.36	−8.70	0.44
*RAI1*	17	17696755	0.4870	0.75	0.30	0.50	0.06
*MAP4*	3	47957741	0.0016	−5.09	0.24	−8.10	0.30
*FLNB*	3	58109162	0.4947	0.39	0.08	1.00	0.27
*ZNF443*	19	12541795	0.3624	−0.65	0.26	−0.77	0.17
*CABP2*	11	67288594	0.3911	0.62	0.21	0.80	0.17
*ZNF544*	19	58772579	0.2150	−0.71	0.18	−0.54	0.05
*LRP8*	1	53712727	0.2117	0.00	0.00	−0.99	0.17
*EPS8L1*	19	55598724	0.4456	0.50	0.14	0.55	0.08
*FLT3*	13	28601297	0.0016	3.24	0.11	4.21	0.09
*MTRR*	5	7897191	0.3660	−0.44	0.10	0.00	0.00
*ZNF17*	19	57931303	0.2993	−0.47	0.10	−0.37	0.03
*HIF3A*	19	46812451	0.0369	0.99	0.09	0.95	0.04
*ZNF180*	19	44983567	0.3417	−0.40	0.08	−0.46	0.05
*GAB2*	11	77937768	0.0082	0.00	0.00	3.09	0.08
*SIX5*	19	46269076	0.3784	0.38	0.08	0.00	0.00
*TCIRG1*	11	67809268	0.0369	1.07	0.08	1.38	0.06
*MTRR*	5	7889304	0.1248	−0.50	0.07	0.00	0.00
*MTRR*	5	7870973	0.2305	−0.38	0.06	0.00	0.00
*MTRR*	5	7891506	0.1230	−0.46	0.06	0.00	0.00
*NEXN*	1	78392446	0.1298	−0.48	0.06	0.00	0.00
*P2RY2*	11	72946204	0.0985	−0.45	0.04	−0.59	0.03
*COL5A3*	19	10085054	0.1448	0.32	0.03	0.54	0.04
*SAT2*	17	7529902	0.0434	0.34	0.01	1.11	0.04
*COL5A3*	19	10085062	0.1349	0.35	0.03	0.59	0.04
*CAPN12*	19	39228244	0.1054	0.30	0.02	0.56	0.03
*ZNF443*	19	12541250	0.0305	−0.58	0.03	−0.69	0.02
*RHOD*	11	66834232	0.0083	−0.95	0.03	0.00	0.00
*RCN3*	19	50045878	0.0417	0.52	0.02	0.96	0.03
*ZNF177*	19	9490760	0.0217	−0.74	0.03	−0.69	0.01
*ZNF443*	19	12541547	0.4150	−0.20	0.02	−0.23	0.02
*SPTBN4*	19	41056229	0.0327	−0.59	0.01	−1.11	0.02
*DNASE1L3*	3	58183636	0.0456	0.36	0.01	0.67	0.02
*EPHA2*	1	16456763	0.0180	0.94	0.02	1.24	0.02
*CYP1A2*	15	75047412	0.0017	0.93	0.02	0.00	0.00
*P2RX5*	17	3599205	0.0170	0.59	0.02	0.00	0.00
*EMP3*	19	48833608	0.0306	0.00	0.00	−0.65	0.02
*CCL24*	7	75442723	0.0148	−0.66	0.01	−1.12	0.02
*CAPN12*	19	39230852	0.0349	0.41	0.01	0.77	0.02
*C1QBP*	17	5338281	0.0098	0.78	0.01	1.38	0.02
*KRT23*	17	39092756	0.1707	0.00	0.00	−0.31	0.01
*RHOD*	11	66837996	0.0065	−0.84	0.01	0.00	0.00
*FPR1*	19	52249211	0.1419	−0.22	0.01	−0.28	0.01
*C9ORF72*	9	27561628	0.0554	0.35	0.01	0.00	0.00
*TCIRG1*	11	67814983	0.0033	0.83	0.01	1.07	0.01
*SNAPC3*	9	15459821	0.0264	−0.46	0.01	−0.60	0.01
*RAI1*	17	17700053	0.0033	0.67	0.01	0.45	<0.01
*BTD*	3	15686693	0.0213	−0.63	0.01	−0.56	<0.01
*SUMF1*	3	4508742	0.1856	0.17	0.01	0.22	0.01

a. Beta = change in mean phenotype value per minor allele carried.

Simulated SBP and DBP varied by sex and increased with age. Effect sizes for these covariates were estimated from the real data set. Simulated DBP was modeled to be an average of 3.715 lower in females and increased by 0.158 per year of age, but only in females. Simulated DBP did not increase by age in males. Simulated SBP was 5.565 lower in females and increased by 0.266 per year of age in males and 0.708 per year of age in females. Age effects were standardized to a mean age of 37.74. Also, paralleling the observed data, simulated cigarette smoking was not related to simulated SBP or DBP.

The total heritability for each simulated phenotype was fixed at the heritability observed in the SAFS data: 0.279 for SBP and 0.317 for DBP. The heritability not accounted for by the variants listed in Additional File 1 was generated using a set of 1000 random variants in genes without main effects from the odd-numbered chromosomes with at least one 3′ or 5′ SNP with MAF > 0.4. These common variants varied across replicates, and each had equal effect sizes. Half were randomly assigned to lower blood pressure and half to raise it. Each of these background variants changed simulated SBP by 0.11187 and simulated DBP by 0.08786 per minor allele carried. The sign of this effect was randomly changed from variant to variant. This polygenic component was further transformed so that it had a zero mean. The mean and variance of simulated SBP and DBP were modeled after the observed SAFS exam 1 data, and the phenotypic variance not accounted for by genetic components was generated by means of a random "environmental" component that was correlated between simulated DBP and SBP and across examinations but uncorrelated between family members.

For each replicate, after SBP and DBP were generated, individuals with SBP > 140 or DBP > 90 were assigned to be hypertensive. A proportion of hypertensive individuals were then chosen to be "treated," and their simulated SBP and DBP were decreased by 6.2 and 7.9, respectively, with the effect of medication being estimated from the SAFS exam 1 data. The probability of a hypertensive individual being medicated was modeled after the real data set and started at 0.55 at exam 1 and rose to 0.67 at exam 2 and 0.82 at exam 3. Individuals carrying coding variants in *CYP3A43 *that were predicted to be deleterious by PolyPhen-2 (Table 4) were assigned to be medication nonresponders, and their blood pressures were not modified regardless of treatment status.

**Table 4:** Deleterious *CYP3A43 *coding variants used in simulated genotype-medication response interaction

Chromosome	Position (bp)	Reference allele	Alternate allele	PolyPhen score	Minor allele frequency
7	99454482	G	A	0.983	0.0068
7	99457518	A	G	0.99	0.0016
7	99457605	C	G	0.925	0.0501

At subsequent simulated examinations, SBP and DBP values were regenerated with the new age at exam using the same genetic values as exam 1 but with a random environmental component that was correlated with the environmental component at exam 1. This led to genetic correlations of *ρ_g _*= 1 within trait across time (eg, SBP at simulated exam 1 with SBP at simulated exam 2 or 3) and equal to that observed at exam 1 for SBP-DBP genetic correlations. Environmental components were also correlated between DBP and SBP. The correlation in environmental components between each pair of simulated blood pressures is given in Table 5. Individuals assigned by simulation to receive hypertensive treatment at exam 1 retained their affection status at subsequent exam regardless of their simulated blood pressures. Additional individuals were diagnosed with hypertension if their exam 2 or exam 3 blood pressures were over the thresholds. Individuals simulated to receive antihypertensive treatment at exam 1 remained on treatment, and an additional proportion of affected individuals began treatment, mimicking the pattern of increasing proportion of affected individuals being treated in later exams seen in the real SAFS data. Cigarette smoking, on the other hand, decreased over the exams with 22.9% of individuals randomly selected to be smokers at exam 1 and 1.45% quitting at each exam to mimic the trend of decreasing smoking seen in the real data set.

**Table 5:** Correlations between simulated random environmental components

	DBP exam 2	DBP exam 3	SBP exam 1	SBP exam 2	SBP exam 3
DBP exam 1	0.41	0.32	0.34	0.14	0.20
DBP exam 2		0.33	0.23	0.46	0.38
DBP exam 3			0.06	0.06	0.48
SBP exam 1				0.57	0.47
SBP exam 2					0.69

Q1 was simulated as a normally distributed quantitative trait that was correlated among family members (additive genetic heritability = 0.68) but not influenced by any of the genotyped SNPs. Mean levels of Q1 were higher in females and decreased with age. Q1 was not influenced by cigarette smoking and was not correlated with simulated SBP, DBP, or hypertension. Measured at only a single simulated exam, Q1 was generated primarily to facilitate assessment of type I error. Given that it was simulated independently of the genotype data, any observed associations were necessarily false positives.

## Conclusions

The GAW18 data set represents the first whole genome sequence distributed for a Genetic Analysis Workshop. These data were newly generated at the time of the workshop, and GAW18 participants had access to them only shortly after the T2D-GENES investigators themselves. Partly because of this, some aspects of the data set were a bit unpolished. For example, Hinrichs et al [11] identified some weaknesses in the imputation methods used and, in fact, T2D-GENES has since redone the imputation. Because of this and because the sample size was subsequently increased, the T2D-GENES Project 2 data now available in the National Institutes of Health dbGaP repository differ from the data distributed for GAW18. Despite these minor weaknesses, the GAW18 data set is still valuable and provides opportunities to address timely analytical challenges through comprehensive WGS in a large sample, complex real phenotypes with complications such as medication effects, and longitudinal data. The simulated phenotypes also presented analytical challenges, in particular, the simulation of small effect sizes with an attempt to build these realistically using observed heritabilities and biologically meaningful weightings of functional variants derived from PolyPhen-2 and from analyses of real gene expression data.

When the data were prepared, we anticipated that GAW18 participants could use them to address a variety of timely problems and issues in statistical genetics methods development. We expected that the WGS and GWAS data would be used to improve approaches for imputing sequence calls in unsequenced family members and to explore the use of existing SNP data for use in error checking of WGS data sets. Methods for gene localization and nomination of potential functional variants have been a consistent focus of the Genetic Analysis Workshops, and we anticipated that this would be a major use of the data. In addition to standard association-based methods of localization, the large pedigrees in the data set made it possible to examine linkage and combined linkage-association approaches. The fact that these families are Mexican American also introduced admixture mapping and population genetics as potential areas of investigation. The inclusion of phenotypic data from multiple examinations facilitated development of methods for genetic analysis of longitudinal data. We also expected that GAW18 participants would seek to use various organizing and filtering principles and additional sources of biological knowledge to focus their analyses and limit the inherent multiple testing in a WGS search. These included grouping sequence variants at the level of genes or pathways or using bioinformatics databases to select variants annotated as coding or regulatory or to place informative prior probabilities on the potential functionality of variants. Indeed, GAW18 participants put these data to all these uses and more.

## Competing interests

The authors declare that they have no competing interests.

## Authors' contributions

TDD, JMP, GJ, ARW, CF, MAA, TWB, DL, GA, SK, JEC, RD, and JB were involved in generation, quality control, data cleaning, and preparation of WGS and genotype data. LA, SF, SP, DL, RD, and JB were involved in generation, quality control, data cleaning, and preparation of SAFS phenotype data. LA, TDD, JMP, JWK, and JB were involved in generation, cleaning, and preparation of simulated phenotype data. LA, RD, and JB drafted the manuscript. All authors read and approved the final manuscript.

## Supplementary Material

Additional file 1 **All simulated functional loci, ordered by chromosome and position**. Click here for file
