# Supplementary material for: Data for Genetic Analysis Workshop 18: human whole genome sequence, blood pressure, and simulated phenotypes in extended pedigrees
Source: BMC Proc. 2014 Jun 17;8(Suppl 1):S2. doi: 10.1186/1753-6561-8-S1-S2 (PMC4145406; doi:10.1186/1753-6561-8-S1-S2)
Supplement: Additional file 1 — All simulated functional loci, ordered by chromosome and position. [file 1753-6561-8-S1-S2-S1.doc]

**Additional file 1**

## All simulated functional loci, ordered by chromosome and position

## Type: N-S, nonsynonymous coding variant; REG, regulatory variant correlated with gene expression. Beta is the change in simulated blood pressure per minor allele carried. PVAR is the percentage of phenotypic variance explained.

| **Chromosome** | **Position** | **Gene** | **MAF** | **Type** | **Beta DBP** | **PVAR DBP** | **Beta SBP** | **PVAR SBP** |
| --- | --- | --- | --- | --- | --- | --- | --- | --- |
| 1 | 1658093 | SLC35E2 | 0.0608 | REG | −0.10 | <0.01 | 0.00 | 0.00 |
| 1 | 1660950 | SLC35E2 | 0.0114 | REG | 0.44 | <0.01 | 0.00 | 0.00 |
| 1 | 1665621 | SLC35E2 | 0.0033 | REG | −0.26 | <0.01 | 0.00 | 0.00 |
| 1 | 1671782 | SLC35E2 | 0.3068 | REG | 0.48 | 0.11 | 0.00 | 0.00 |
| 1 | 1672142 | SLC35E2 | 0.3001 | REG | −0.57 | 0.14 | 0.00 | 0.00 |
| 1 | 1672715 | SLC35E2 | 0.4897 | REG | 0.40 | 0.09 | 0.00 | 0.00 |
| 1 | 1680036 | SLC35E2 | 0.4971 | REG | 0.15 | 0.01 | 0.00 | 0.00 |
| 1 | 1748361 | GNB1 | 0.0033 | REG | 0.00 | 0.00 | −0.51 | <0.01 |
| 1 | 1749995 | GNB1 | 0.0033 | REG | 0.00 | 0.00 | −0.76 | <0.01 |
| 1 | 1767565 | GNB1 | 0.4635 | REG | 0.00 | 0.00 | 0.06 | <0.01 |
| 1 | 1773674 | GNB1 | 0.3855 | REG | 0.00 | 0.00 | 0.06 | <0.01 |
| 1 | 2441077 | PANK4 | 0.0033 | REG | −0.31 | <0.01 | −0.26 | <0.01 |
| 1 | 2441358 | PANK4 | 0.3628 | N-S | 0.00 | 0.00 | 0.00 | 0.00 |
| 1 | 2442686 | PANK4 | 0.0181 | REG | 0.26 | <0.01 | 0.22 | <0.01 |
| 1 | 2444414 | PANK4 | 0.2468 | N-S | 0.00 | 0.00 | 0.00 | 0.00 |
| 1 | 2444469 | PANK4 | 0.0271 | N-S | −0.17 | <0.01 | −0.20 | <0.01 |
| 1 | 2450394 | PANK4 | 0.3769 | REG | 0.12 | 0.01 | 0.11 | <0.01 |
| 1 | 2452744 | PANK4 | 0.0049 | N-S | 0.00 | 0.00 | 0.00 | 0.00 |
| 1 | 2456781 | PANK4 | 0.0033 | REG | 0.26 | <0.01 | 0.23 | <0.01 |
| 1 | 16402308 | EPHA2 | 0.0117 | REG | 0.37 | 0.01 | 0.35 | <0.01 |
| 1 | 16450546 | EPHA2 | 0.0016 | REG | −0.91 | 0.01 | −0.87 | <0.01 |
| 1 | 16456763 | EPHA2 | 0.0180 | N-S | 0.94 | 0.02 | 1.24 | 0.02 |
| 1 | 16459836 | EPHA2 | 0.0065 | N-S | 0.00 | 0.00 | 0.00 | 0.00 |
| 1 | 16461546 | EPHA2 | 0.0065 | N-S | 0.04 | <0.01 | 0.05 | <0.01 |
| 1 | 16464489 | EPHA2 | 0.1291 | N-S | 0.00 | 0.00 | 0.00 | 0.00 |
| 1 | 16521427 | EPHA2 | 0.0429 | REG | −0.21 | 0.01 | −0.20 | <0.01 |
| 1 | 16521438 | EPHA2 | 0.0692 | REG | −0.32 | 0.01 | −0.30 | 0.01 |
| 1 | 16521721 | EPHA2 | 0.0228 | REG | 0.41 | 0.01 | 0.40 | <0.01 |
| 1 | 18957349 | PAX7 | 0.0180 | REG | 0.44 | <0.01 | 0.00 | 0.00 |
| 1 | 19003477 | PAX7 | 0.0065 | REG | −0.43 | <0.01 | 0.00 | 0.00 |
| 1 | 19016516 | PAX7 | 0.0150 | REG | 0.26 | <0.01 | 0.00 | 0.00 |
| 1 | 19066969 | PAX7 | 0.0419 | REG | −0.23 | <0.01 | 0.00 | 0.00 |
| 1 | 19075204 | PAX7 | 0.0883 | REG | −0.17 | <0.01 | 0.00 | 0.00 |
| 1 | 19076285 | PAX7 | 0.0033 | REG | −0.37 | <0.01 | 0.00 | 0.00 |
| 1 | 19079624 | PAX7 | 0.0457 | REG | −0.17 | <0.01 | 0.00 | 0.00 |
| 1 | 20910770 | CDA | 0.4680 | REG | 0.06 | <0.01 | 0.06 | <0.01 |
| 1 | 20915418 | CDA | 0.3544 | REG | 0.09 | <0.01 | 0.08 | <0.01 |
| 1 | 20915701 | CDA | 0.2909 | N-S | 0.00 | 0.00 | 0.00 | 0.00 |
| 1 | 20924330 | CDA | 0.1897 | REG | 0.25 | 0.02 | 0.23 | 0.01 |
| 1 | 20926488 | CDA | 0.0033 | REG | 0.36 | <0.01 | 0.34 | <0.01 |
| 1 | 20932028 | CDA | 0.0728 | REG | −0.14 | <0.01 | −0.13 | <0.01 |
| 1 | 20932697 | CDA | 0.0374 | REG | 0.17 | <0.01 | 0.16 | <0.01 |
| 1 | 21155679 | EIF4G3 | 0.0035 | N-S | −0.42 | 0.01 | −0.48 | <0.01 |
| 1 | 21156862 | EIF4G3 | 0.2396 | REG | 0.29 | 0.04 | 0.24 | 0.01 |
| 1 | 21201623 | EIF4G3 | 0.0033 | REG | 0.55 | <0.01 | 0.46 | <0.01 |
| 1 | 21207497 | EIF4G3 | 0.0545 | REG | −0.16 | <0.01 | −0.13 | <0.01 |
| 1 | 21220089 | EIF4G3 | 0.0016 | N-S | −0.33 | <0.01 | −0.38 | <0.01 |
| 1 | 21247365 | EIF4G3 | 0.2342 | REG | −0.35 | 0.06 | −0.29 | 0.02 |
| 1 | 21266411 | EIF4G3 | 0.0149 | REG | −0.25 | <0.01 | −0.20 | <0.01 |
| 1 | 21267993 | EIF4G3 | 0.0065 | N-S | −0.42 | <0.01 | −0.48 | <0.01 |
| 1 | 21275335 | EIF4G3 | 0.0082 | REG | −0.22 | <0.01 | −0.19 | <0.01 |
| 1 | 21296910 | EIF4G3 | 0.0099 | REG | 0.26 | <0.01 | 0.22 | <0.01 |
| 1 | 21302893 | EIF4G3 | 0.1141 | REG | 0.11 | <0.01 | 0.09 | <0.01 |
| 1 | 21315739 | EIF4G3 | 0.0049 | REG | 0.41 | <0.01 | 0.35 | <0.01 |
| 1 | 21345856 | EIF4G3 | 0.0131 | REG | −0.18 | <0.01 | −0.15 | <0.01 |
| 1 | 21441136 | EIF4G3 | 0.0082 | REG | −0.65 | 0.01 | −0.54 | <0.01 |
| 1 | 21498875 | EIF4G3 | 0.0278 | REG | −0.18 | <0.01 | −0.15 | <0.01 |
| 1 | 22931091 | C1QA | 0.2457 | REG | −0.04 | <0.01 | −0.04 | <0.01 |
| 1 | 22959353 | C1QA | 0.0182 | REG | −0.17 | <0.01 | −0.20 | <0.01 |
| 1 | 22961947 | C1QA | 0.4527 | REG | −0.05 | <0.01 | −0.06 | <0.01 |
| 1 | 22963050 | C1QA | 0.0417 | REG | 0.21 | <0.01 | 0.24 | <0.01 |
| 1 | 22968610 | C1QA | 0.0400 | REG | 0.17 | <0.01 | 0.19 | <0.01 |
| 1 | 24025181 | TCEB3 | 0.2913 | REG | −0.12 | 0.01 | −0.07 | <0.01 |
| 1 | 24027111 | TCEB3 | 0.0049 | REG | −0.46 | <0.01 | −0.27 | <0.01 |
| 1 | 24074044 | TCEB3 | 0.0049 | REG | −0.75 | <0.01 | −0.44 | <0.01 |
| 1 | 24077451 | TCEB3 | 0.1418 | N-S | 0.00 | 0.00 | 0.00 | 0.00 |
| 1 | 24077957 | TCEB3 | 0.0016 | N-S | 0.00 | 0.00 | 0.00 | 0.00 |
| 1 | 24077987 | TCEB3 | 0.0018 | N-S | 0.00 | 0.00 | 0.00 | 0.00 |
| 1 | 24078234 | TCEB3 | 0.0180 | N-S | 0.00 | 0.00 | 0.00 | 0.00 |
| 1 | 24078903 | TCEB3 | 0.0016 | N-S | 0.00 | 0.00 | 0.00 | 0.00 |
| 1 | 24078940 | TCEB3 | 0.0018 | N-S | 0.00 | 0.00 | 0.00 | 0.00 |
| 1 | 26114310 | SEPN1 | 0.0164 | REG | 0.00 | 0.00 | −0.24 | <0.01 |
| 1 | 26143709 | SEPN1 | 0.0033 | REG | 0.00 | 0.00 | 0.38 | <0.01 |
| 1 | 26224302 | STMN1 | 0.0286 | REG | 0.19 | <0.01 | 0.00 | 0.00 |
| 1 | 26284164 | STMN1 | 0.0066 | REG | 0.49 | <0.01 | 0.00 | 0.00 |
| 1 | 27681534 | MAP3K6 | 0.2983 | REG | −0.11 | 0.01 | −0.09 | <0.01 |
| 1 | 27682530 | MAP3K6 | 0.0516 | N-S | 0.00 | 0.00 | 0.00 | 0.00 |
| 1 | 27683836 | MAP3K6 | 0.0050 | REG | −0.62 | 0.01 | −0.50 | <0.01 |
| 1 | 27687101 | MAP3K6 | 0.0332 | REG | 0.27 | 0.01 | 0.22 | <0.01 |
| 1 | 27687466 | MAP3K6 | 0.1528 | N-S | −0.01 | <0.01 | −0.01 | <0.01 |
| 1 | 27688633 | MAP3K6 | 0.1754 | N-S | 0.00 | 0.00 | 0.00 | 0.00 |
| 1 | 27688663 | MAP3K6 | 0.0033 | N-S | −0.60 | <0.01 | −0.66 | <0.01 |
| 1 | 27689421 | MAP3K6 | 0.0016 | N-S | −0.53 | <0.01 | −0.58 | <0.01 |
| 1 | 27690770 | MAP3K6 | 0.0756 | N-S | 0.00 | 0.00 | 0.00 | 0.00 |
| 1 | 27942656 | FGR | 0.0179 | REG | 0.36 | <0.01 | 0.26 | <0.01 |
| 1 | 27949569 | FGR | 0.0033 | N-S | −0.47 | <0.01 | −0.47 | <0.01 |
| 1 | 27951127 | FGR | 0.0760 | REG | 0.11 | <0.01 | 0.08 | <0.01 |
| 1 | 27988214 | FGR | 0.0082 | REG | 0.30 | <0.01 | 0.22 | <0.01 |
| 1 | 27989620 | FGR | 0.3104 | REG | 0.07 | <0.01 | 0.05 | <0.01 |
| 1 | 27991061 | FGR | 0.0535 | REG | −0.19 | <0.01 | −0.14 | <0.01 |
| 1 | 28155332 | PPP1R8 | 0.0671 | REG | −0.16 | <0.01 | 0.00 | 0.00 |
| 1 | 28155886 | PPP1R8 | 0.0658 | REG | −0.12 | <0.01 | 0.00 | 0.00 |
| 1 | 28160447 | PPP1R8 | 0.0065 | REG | −0.61 | <0.01 | 0.00 | 0.00 |
| 1 | 28168022 | PPP1R8 | 0.0065 | REG | −0.76 | <0.01 | 0.00 | 0.00 |
| 1 | 28171620 | PPP1R8 | 0.0082 | REG | 0.28 | <0.01 | 0.00 | 0.00 |
| 1 | 28176716 | PPP1R8 | 0.0065 | N-S | 0.00 | 0.00 | 0.00 | 0.00 |
| 1 | 39472934 | NDUFS5 | 0.0016 | REG | 0.48 | <0.01 | 0.37 | <0.01 |
| 1 | 39474057 | NDUFS5 | 0.2341 | REG | −0.11 | 0.01 | −0.09 | <0.01 |
| 1 | 40439598 | CAP1 | 0.3487 | REG | 0.06 | <0.01 | 0.09 | <0.01 |
| 1 | 40510432 | CAP1 | 0.0564 | REG | −0.10 | <0.01 | −0.17 | <0.01 |
| 1 | 40722795 | ZMPSTE24 | 0.1914 | REG | 0.07 | <0.01 | 0.07 | <0.01 |
| 1 | 40733907 | ZMPSTE24 | 0.0016 | REG | 0.44 | <0.01 | 0.42 | <0.01 |
| 1 | 41329534 | CITED4 | 0.3855 | REG | 0.10 | 0.01 | 0.00 | 0.00 |
| 1 | 41332243 | CITED4 | 0.0017 | REG | −0.23 | <0.01 | 0.00 | 0.00 |
| 1 | 41346344 | CITED4 | 0.3421 | REG | −0.08 | <0.01 | 0.00 | 0.00 |
| 1 | 41349666 | CITED4 | 0.2928 | REG | 0.03 | <0.01 | 0.00 | 0.00 |
| 1 | 41351412 | CITED4 | 0.0016 | REG | 0.48 | <0.01 | 0.00 | 0.00 |
| 1 | 41351541 | CITED4 | 0.3963 | REG | −0.12 | 0.01 | 0.00 | 0.00 |
| 1 | 41443294 | CITED4 | 0.1089 | REG | 0.13 | <0.01 | 0.00 | 0.00 |
| 1 | 43837921 | MED8 | 0.0049 | REG | 0.40 | <0.01 | 0.19 | <0.01 |
| 1 | 43850473 | MED8 | 0.2964 | REG | −0.22 | 0.03 | −0.11 | <0.01 |
| 1 | 45987574 | PRDX1 | 0.2071 | REG | 0.10 | <0.01 | 0.09 | <0.01 |
| 1 | 53712727 | LRP8 | 0.2117 | N-S | 0.00 | 0.00 | −0.99 | 0.17 |
| 1 | 53722932 | LRP8 | 0.0099 | N-S | 0.00 | 0.00 | 0.00 | 0.00 |
| 1 | 53730037 | LRP8 | 0.0033 | N-S | 0.00 | 0.00 | −0.22 | <0.01 |
| 1 | 53792651 | LRP8 | 0.2856 | N-S | 0.00 | 0.00 | 0.00 | 0.00 |
| 1 | 65912722 | LEPR | 0.0033 | REG | 1.53 | 0.05 | 1.66 | 0.03 |
| 1 | 65914232 | LEPR | 0.0950 | REG | −0.69 | 0.08 | −0.74 | 0.04 |
| 1 | 66036441 | LEPR | 0.2950 | N-S | 0.00 | 0.00 | 0.00 | 0.00 |
| 1 | 66056270 | LEPR | 0.0049 | REG | 3.08 | 0.10 | 3.34 | 0.06 |
| 1 | 66058513 | LEPR | 0.4402 | N-S | 0.03 | <0.01 | 0.05 | <0.01 |
| 1 | 66064113 | LEPR | 0.4382 | REG | −0.38 | 0.08 | −0.41 | 0.04 |
| 1 | 66075690 | LEPR | 0.0065 | N-S | 0.09 | <0.01 | 0.13 | <0.01 |
| 1 | 66075952 | LEPR | 0.1567 | N-S | 2.76 | 2.19 | 3.87 | 2.06 |
| 1 | 78376190 | NEXN | 0.0033 | REG | −0.42 | <0.01 | 0.00 | 0.00 |
| 1 | 78392446 | NEXN | 0.1298 | N-S | −0.48 | 0.06 | 0.00 | 0.00 |
| 1 | 78400268 | NEXN | 0.0033 | REG | −0.37 | <0.01 | 0.00 | 0.00 |
| 1 | 89642985 | GBP4 | 0.0033 | REG | −0.27 | <0.01 | −0.18 | <0.01 |
| 1 | 89652078 | GBP4 | 0.3458 | N-S | 0.00 | 0.00 | 0.00 | 0.00 |
| 1 | 89652097 | GBP4 | 0.3295 | N-S | 0.00 | 0.00 | 0.00 | 0.00 |
| 1 | 89652102 | GBP4 | 0.3241 | N-S | 0.00 | 0.00 | 0.00 | 0.00 |
| 1 | 89655783 | GBP4 | 0.0130 | N-S | 0.00 | 0.00 | 0.00 | 0.00 |
| 1 | 89661089 | GBP4 | 0.0033 | N-S | −0.58 | <0.01 | −0.55 | <0.01 |
| 1 | 89665523 | GBP4 | 0.1876 | REG | 0.10 | <0.01 | 0.07 | <0.01 |
| 1 | 89722911 | GBP4 | 0.0016 | REG | 0.38 | <0.01 | 0.26 | <0.01 |
| 1 | 100819366 | CDC14A | 0.0049 | N-S | 0.00 | 0.00 | 0.00 | 0.00 |
| 1 | 100889098 | CDC14A | 0.0098 | REG | 0.00 | 0.00 | 0.23 | <0.01 |
| 1 | 100890359 | CDC14A | 0.0679 | REG | 0.00 | 0.00 | −0.31 | 0.01 |
| 1 | 100928377 | CDC14A | 0.0016 | N-S | 0.00 | 0.00 | 0.00 | 0.00 |
| 1 | 100929453 | CDC14A | 0.0147 | REG | 0.00 | 0.00 | −0.24 | <0.01 |
| 1 | 100931073 | CDC14A | 0.4676 | REG | 0.00 | 0.00 | 0.06 | <0.01 |
| 1 | 100956077 | CDC14A | 0.0016 | REG | 0.00 | 0.00 | −0.36 | <0.01 |
| 1 | 100975996 | CDC14A | 0.0183 | REG | 0.00 | 0.00 | 0.14 | <0.01 |
| 1 | 100977271 | CDC14A | 0.0677 | REG | 0.00 | 0.00 | 0.22 | <0.01 |
| 1 | 104211332 | AMY1A | 0.0480 | REG | 0.00 | 0.00 | 0.16 | <0.01 |
| 1 | 104303222 | AMY1A | 0.0215 | REG | 0.00 | 0.00 | −0.13 | <0.01 |
| 1 | 104613453 | AMY1A | 0.0050 | REG | 0.00 | 0.00 | 0.26 | <0.01 |
| 1 | 109757295 | SARS | 0.0149 | REG | −0.43 | <0.01 | 0.00 | 0.00 |
| 1 | 110478311 | AHCYL1 | 0.0016 | REG | 0.00 | 0.00 | 0.62 | <0.01 |
| 1 | 110522900 | AHCYL1 | 0.0065 | REG | 0.00 | 0.00 | −0.53 | <0.01 |
| 1 | 110528231 | AHCYL1 | 0.0016 | REG | 0.00 | 0.00 | 0.57 | <0.01 |
| 1 | 110557015 | AHCYL1 | 0.0840 | REG | 0.00 | 0.00 | 0.17 | <0.01 |
| 1 | 110560915 | AHCYL1 | 0.1037 | REG | 0.00 | 0.00 | 0.14 | <0.01 |
| 1 | 112321586 | KCND3 | 0.0098 | REG | −0.50 | <0.01 | −0.59 | <0.01 |
| 1 | 112326392 | KCND3 | 0.0033 | REG | 0.74 | <0.01 | 0.86 | <0.01 |
| 1 | 112355413 | KCND3 | 0.0115 | REG | 0.36 | <0.01 | 0.42 | <0.01 |
| 1 | 112391401 | KCND3 | 0.1202 | REG | −0.10 | <0.01 | −0.12 | <0.01 |
| 1 | 112393959 | KCND3 | 0.0165 | REG | −0.21 | <0.01 | −0.25 | <0.01 |
| 1 | 112444219 | KCND3 | 0.0033 | REG | −0.44 | <0.01 | −0.52 | <0.01 |
| 1 | 112468634 | KCND3 | 0.0049 | REG | −0.52 | <0.01 | −0.62 | <0.01 |
| 1 | 112914056 | KCND3 | 0.0065 | REG | −0.42 | <0.01 | −0.49 | <0.01 |
| 1 | 113535271 | LRIG2 | 0.0115 | REG | −0.42 | <0.01 | −0.26 | <0.01 |
| 1 | 113591119 | LRIG2 | 0.0081 | REG | 0.61 | <0.01 | 0.38 | <0.01 |
| 1 | 113657233 | LRIG2 | 0.0115 | N-S | 0.14 | <0.01 | 0.12 | <0.01 |
| 1 | 113660097 | LRIG2 | 0.1762 | REG | 0.16 | 0.01 | 0.10 | <0.01 |
| 1 | 113661918 | LRIG2 | 0.0082 | N-S | 0.20 | <0.01 | 0.17 | <0.01 |
| 1 | 113671013 | LRIG2 | 0.0116 | REG | −0.22 | <0.01 | −0.14 | <0.01 |
| 1 | 118409721 | GDAP2 | 0.0033 | REG | 0.45 | <0.01 | 0.59 | <0.01 |
| 1 | 118420943 | GDAP2 | 0.0016 | REG | −0.76 | <0.01 | −1.00 | <0.01 |
| 1 | 118468511 | GDAP2 | 0.0236 | REG | −0.24 | <0.01 | −0.31 | <0.01 |
| 1 | 119575818 | WARS2 | 0.0295 | N-S | 0.00 | 0.00 | 0.00 | 0.00 |
| 1 | 119591844 | WARS2 | 0.0016 | REG | −0.72 | 0.01 | −0.49 | <0.01 |
| 1 | 119593659 | WARS2 | 0.1445 | REG | −0.36 | 0.04 | −0.25 | 0.01 |
| 1 | 119600175 | WARS2 | 0.1013 | REG | 0.41 | 0.04 | 0.28 | 0.01 |
| 1 | 119617449 | WARS2 | 0.0114 | REG | 0.62 | 0.01 | 0.42 | <0.01 |
| 1 | 119632815 | WARS2 | 0.0197 | REG | −0.44 | 0.01 | −0.30 | <0.01 |
| 1 | 119674088 | WARS2 | 0.1409 | REG | 0.21 | 0.01 | 0.14 | <0.01 |
| 1 | 119683231 | WARS2 | 0.0115 | N-S | 0.97 | 0.01 | 0.91 | 0.01 |
| 1 | 119699582 | WARS2 | 0.0101 | REG | −0.30 | 0.01 | −0.21 | <0.01 |
| 1 | 119780359 | WARS2 | 0.0067 | REG | −0.65 | 0.01 | −0.45 | <0.01 |
| 1 | 119799041 | WARS2 | 0.0033 | REG | −0.63 | 0.01 | −0.43 | <0.01 |
| 1 | 145433850 | TXNIP | 0.0098 | REG | 0.28 | <0.01 | 0.21 | <0.01 |
| 1 | 145439846 | TXNIP | 0.0016 | N-S | −0.57 | <0.01 | −0.61 | <0.01 |
| 1 | 145440086 | TXNIP | 0.0065 | N-S | −0.06 | <0.01 | −0.06 | <0.01 |
| 1 | 145444883 | TXNIP | 0.2247 | REG | −0.21 | 0.01 | −0.16 | <0.01 |
| 1 | 145454421 | TXNIP | 0.0033 | REG | 0.62 | <0.01 | 0.47 | <0.01 |
| 1 | 150798577 | ARNT | 0.4174 | REG | −0.24 | 0.03 | 0.00 | 0.00 |
| 1 | 150798976 | ARNT | 0.0033 | REG | 0.48 | <0.01 | 0.00 | 0.00 |
| 1 | 151140732 | SCNM1 | 0.0065 | N-S | 0.00 | 0.00 | 0.00 | 0.00 |
| 1 | 151141485 | SCNM1 | 0.0049 | N-S | 0.45 | <0.01 | 1.04 | 0.01 |
| 1 | 151491026 | CGN | 0.0049 | N-S | −1.90 | 0.08 | 0.00 | 0.00 |
| 1 | 151491836 | CGN | 0.0050 | N-S | −1.81 | 0.08 | 0.00 | 0.00 |
| 1 | 151493096 | CGN | 0.0033 | N-S | 0.00 | 0.00 | 0.00 | 0.00 |
| 1 | 151496012 | CGN | 0.0067 | N-S | −0.69 | 0.02 | 0.00 | 0.00 |
| 1 | 151496718 | CGN | 0.0216 | N-S | −0.13 | <0.01 | 0.00 | 0.00 |
| 1 | 151497202 | CGN | 0.1453 | N-S | 0.00 | 0.00 | 0.00 | 0.00 |
| 1 | 151498143 | CGN | 0.0065 | N-S | 0.00 | 0.00 | 0.00 | 0.00 |
| 1 | 151501841 | CGN | 0.1245 | N-S | −1.49 | 0.60 | 0.00 | 0.00 |
| 1 | 151501901 | CGN | 0.0033 | N-S | −1.12 | 0.01 | 0.00 | 0.00 |
| 1 | 151501908 | CGN | 0.0016 | N-S | 0.00 | 0.00 | 0.00 | 0.00 |
| 1 | 151502427 | CGN | 0.0149 | N-S | −0.38 | 0.01 | 0.00 | 0.00 |
| 1 | 151502589 | CGN | 0.0049 | N-S | −1.55 | 0.02 | 0.00 | 0.00 |
| 1 | 151502978 | CGN | 0.0017 | N-S | 0.00 | 0.00 | 0.00 | 0.00 |
| 1 | 151505035 | CGN | 0.0017 | N-S | −0.74 | 0.01 | 0.00 | 0.00 |
| 1 | 151506526 | CGN | 0.0233 | N-S | 0.00 | 0.00 | 0.00 | 0.00 |
| 1 | 151509754 | CGN | 0.0033 | N-S | 0.00 | 0.00 | 0.00 | 0.00 |
| 1 | 153324927 | S100A9 | 0.0016 | REG | 0.37 | <0.01 | 0.48 | <0.01 |
| 1 | 153330777 | S100A9 | 0.0033 | REG | 0.34 | <0.01 | 0.44 | <0.01 |
| 1 | 153337943 | S100A12 | 0.1922 | REG | 0.06 | <0.01 | 0.10 | <0.01 |
| 1 | 153344636 | S100A12 | 0.0877 | REG | −0.19 | 0.01 | −0.31 | 0.01 |
| 1 | 153435267 | S100A6 | 0.0033 | REG | 0.00 | 0.00 | −0.39 | <0.01 |
| 1 | 153502121 | S100A6 | 0.1123 | REG | 0.00 | 0.00 | 0.16 | <0.01 |
| 1 | 153507176 | S100A6 | 0.0049 | N-S | 0.00 | 0.00 | −0.04 | <0.01 |
| 1 | 153507809 | S100A6 | 0.0099 | N-S | 0.00 | 0.00 | −0.41 | <0.01 |
| 1 | 154149240 | TPM3 | 0.3291 | REG | 0.06 | <0.01 | 0.08 | <0.01 |
| 1 | 155210498 | GBA | 0.0115 | N-S | 0.00 | 0.00 | 0.00 | 0.00 |
| 1 | 155215402 | GBA | 0.1571 | REG | −0.10 | <0.01 | 0.00 | 0.00 |
| 1 | 155261709 | PKLR | 0.0033 | N-S | 1.23 | 0.03 | 1.87 | 0.03 |
| 1 | 155268408 | PKLR | 0.0065 | REG | 0.88 | 0.01 | 0.96 | 0.01 |
| 1 | 155837865 | SYT11 | 0.0228 | N-S | 0.00 | 0.00 | 0.00 | 0.00 |
| 1 | 156054035 | LMNA | 0.0082 | REG | −0.19 | <0.01 | 0.00 | 0.00 |
| 1 | 156077363 | LMNA | 0.0033 | REG | −0.20 | <0.01 | 0.00 | 0.00 |
| 1 | 156081019 | LMNA | 0.0033 | REG | −0.42 | <0.01 | 0.00 | 0.00 |
| 1 | 156089324 | LMNA | 0.0747 | REG | 0.41 | 0.03 | 0.00 | 0.00 |
| 1 | 156092565 | LMNA | 0.0016 | REG | 0.27 | <0.01 | 0.00 | 0.00 |
| 1 | 156096205 | LMNA | 0.0133 | REG | −0.13 | <0.01 | 0.00 | 0.00 |
| 1 | 156100239 | LMNA | 0.0016 | REG | 0.39 | <0.01 | 0.00 | 0.00 |
| 1 | 156105062 | LMNA | 0.0082 | N-S | 0.00 | 0.00 | 0.00 | 0.00 |
| 1 | 156255327 | TMEM79 | 0.0082 | N-S | 0.00 | 0.00 | 0.00 | 0.00 |
| 1 | 156255456 | TMEM79 | 0.2808 | N-S | 0.00 | 0.00 | 0.00 | 0.00 |
| 1 | 156255576 | TMEM79 | 0.0016 | N-S | 0.00 | 0.00 | 0.00 | 0.00 |
| 1 | 156256116 | TMEM79 | 0.0016 | N-S | −0.40 | <0.01 | 0.00 | 0.00 |
| 1 | 156260644 | TMEM79 | 0.3725 | REG | 0.08 | <0.01 | 0.00 | 0.00 |
| 1 | 158150619 | CD1D | 0.0098 | REG | 0.00 | 0.00 | −0.52 | <0.01 |
| 1 | 158152716 | CD1D | 0.0033 | N-S | 0.00 | 0.00 | 0.00 | 0.00 |
| 1 | 158174400 | CD1D | 0.0033 | REG | 0.00 | 0.00 | −0.32 | <0.01 |
| 1 | 158261888 | CD1C | 0.0049 | REG | −0.25 | <0.01 | −0.45 | <0.01 |
| 1 | 158267777 | CD1C | 0.0649 | REG | 0.07 | <0.01 | 0.13 | <0.01 |
| 1 | 159275976 | FCER1A | 0.0180 | N-S | 0.05 | <0.01 | 0.06 | <0.01 |
| 1 | 159277689 | FCER1A | 0.0177 | N-S | 0.03 | <0.01 | 0.05 | <0.01 |
| 1 | 163051259 | RGS5 | 0.0049 | REG | −0.36 | <0.01 | 0.00 | 0.00 |
| 1 | 163114891 | RGS5 | 0.0083 | REG | −0.45 | <0.01 | 0.00 | 0.00 |
| 1 | 163142027 | RGS5 | 0.0033 | REG | −0.37 | <0.01 | 0.00 | 0.00 |
| 1 | 163161795 | RGS5 | 0.0268 | REG | 0.27 | <0.01 | 0.00 | 0.00 |
| 1 | 163190469 | RGS5 | 0.0049 | REG | −0.73 | <0.01 | 0.00 | 0.00 |
| 1 | 163253512 | RGS5 | 0.0049 | REG | 0.60 | <0.01 | 0.00 | 0.00 |
| 1 | 174996637 | TNN | 0.3686 | REG | 0.26 | 0.03 | 0.32 | 0.02 |
| 1 | 175046652 | TNN | 0.0148 | N-S | 4.48 | 0.48 | 6.31 | 0.46 |
| 1 | 175046762 | TNN | 0.0066 | N-S | 0.00 | 0.00 | 0.00 | 0.00 |
| 1 | 175046789 | TNN | 0.4981 | N-S | 0.00 | 0.00 | 0.00 | 0.00 |
| 1 | 175046826 | TNN | 0.0198 | N-S | 0.05 | <0.01 | 0.07 | <0.01 |
| 1 | 175046835 | TNN | 0.0098 | N-S | 3.43 | 0.19 | 4.83 | 0.18 |
| 1 | 175046920 | TNN | 0.0016 | N-S | 0.00 | 0.00 | 0.01 | <0.01 |
| 1 | 175049379 | TNN | 0.0777 | N-S | 0.15 | <0.01 | 0.24 | <0.01 |
| 1 | 175054626 | TNN | 0.0082 | N-S | 4.22 | 0.29 | 5.94 | 0.27 |
| 1 | 175086261 | TNN | 0.0016 | N-S | 4.82 | 0.25 | 6.79 | 0.24 |
| 1 | 175087729 | TNN | 0.3458 | N-S | 0.01 | <0.01 | 0.01 | <0.01 |
| 1 | 175092622 | TNN | 0.0016 | N-S | 4.47 | 0.19 | 6.30 | 0.18 |
| 1 | 175092631 | TNN | 0.0049 | N-S | 0.01 | <0.01 | 0.01 | <0.01 |
| 1 | 175092637 | TNN | 0.0066 | N-S | 4.83 | 0.53 | 6.80 | 0.50 |
| 1 | 175092674 | TNN | 0.3161 | N-S | 2.01 | 1.98 | 2.84 | 1.89 |
| 1 | 175092707 | TNN | 0.2344 | N-S | 0.00 | 0.00 | 0.00 | 0.00 |
| 1 | 175097264 | TNN | 0.0016 | N-S | 4.06 | 0.13 | 5.72 | 0.13 |
| 1 | 175105996 | TNN | 0.1147 | N-S | 0.00 | 0.00 | 0.00 | 0.00 |
| 1 | 179049606 | TOR3A | 0.3813 | REG | 0.10 | <0.01 | 0.00 | 0.00 |
| 1 | 179051300 | TOR3A | 0.3931 | N-S | 0.00 | 0.00 | 0.00 | 0.00 |
| 1 | 179059258 | TOR3A | 0.0033 | REG | 0.25 | <0.01 | 0.00 | 0.00 |
| 1 | 179061317 | TOR3A | 0.1382 | REG | 0.15 | 0.01 | 0.00 | 0.00 |
| 1 | 179064142 | TOR3A | 0.0066 | N-S | 0.39 | 0.01 | 0.00 | 0.00 |
| 1 | 179064875 | TOR3A | 0.4005 | REG | −0.18 | 0.02 | 0.00 | 0.00 |
| 1 | 179065563 | TOR3A | 0.0638 | REG | 0.15 | <0.01 | 0.00 | 0.00 |
| 1 | 182032009 | GLUL | 0.1247 | REG | 0.16 | 0.01 | 0.18 | <0.01 |
| 1 | 182345984 | GLUL | 0.0049 | REG | 0.59 | <0.01 | 0.63 | <0.01 |
| 1 | 182348016 | GLUL | 0.0724 | REG | 0.16 | <0.01 | 0.17 | <0.01 |
| 1 | 182348380 | GLUL | 0.0082 | REG | −0.43 | <0.01 | −0.46 | <0.01 |
| 1 | 182352981 | GLUL | 0.0016 | REG | −0.90 | 0.01 | −0.96 | <0.01 |
| 1 | 182354890 | GLUL | 0.0033 | REG | 0.95 | 0.01 | 1.02 | <0.01 |
| 1 | 182355604 | GLUL | 0.0098 | REG | 0.71 | 0.01 | 0.76 | <0.01 |
| 1 | 183661246 | RGL1 | 0.0298 | REG | −0.33 | 0.01 | −0.32 | <0.01 |
| 1 | 183721974 | RGL1 | 0.0066 | REG | 0.80 | 0.01 | 0.80 | <0.01 |
| 1 | 183738218 | RGL1 | 0.0066 | REG | −2.05 | 0.01 | −2.04 | 0.01 |
| 1 | 183785892 | RGL1 | 0.1938 | REG | 0.10 | <0.01 | 0.10 | <0.01 |
| 1 | 183842235 | RGL1 | 0.0065 | REG | 0.50 | <0.01 | 0.50 | <0.01 |
| 1 | 183846650 | RGL1 | 0.0016 | REG | −0.90 | 0.01 | −0.90 | <0.01 |
| 1 | 183850225 | RGL1 | 0.0033 | REG | −0.68 | <0.01 | −0.67 | <0.01 |
| 1 | 183893194 | RGL1 | 0.0016 | REG | 0.88 | 0.01 | 0.87 | <0.01 |
| 1 | 183896333 | RGL1 | 0.0049 | REG | 0.62 | <0.01 | 0.62 | <0.01 |
| 1 | 186282622 | TPR | 0.0082 | REG | 0.30 | <0.01 | 0.00 | 0.00 |
| 1 | 186289510 | TPR | 0.0696 | N-S | −0.48 | 0.03 | 0.00 | 0.00 |
| 1 | 186313197 | TPR | 0.0049 | N-S | −0.23 | <0.01 | 0.00 | 0.00 |
| 1 | 186316488 | TPR | 0.0506 | N-S | −0.11 | <0.01 | 0.00 | 0.00 |
| 1 | 186321242 | TPR | 0.0754 | N-S | −0.16 | <0.01 | 0.00 | 0.00 |
| 1 | 186324810 | TPR | 0.0016 | N-S | −0.06 | <0.01 | 0.00 | 0.00 |
| 1 | 186328958 | TPR | 0.0033 | REG | 0.49 | <0.01 | 0.00 | 0.00 |
| 1 | 186342527 | TPR | 0.0066 | N-S | −0.12 | <0.01 | 0.00 | 0.00 |
| 1 | 204378775 | PPP1R15B | 0.0098 | N-S | 0.00 | 0.00 | 0.00 | 0.00 |
| 1 | 204379452 | PPP1R15B | 0.0264 | N-S | 0.00 | 0.00 | 0.00 | 0.00 |
| 1 | 204379617 | PPP1R15B | 0.0082 | N-S | 0.00 | 0.00 | 0.00 | 0.00 |
| 1 | 204380110 | PPP1R15B | 0.0033 | N-S | 0.35 | <0.01 | 0.00 | 0.00 |
| 1 | 204389134 | PPP1R15B | 0.0230 | REG | −0.26 | <0.01 | 0.00 | 0.00 |
| 1 | 204390204 | PPP1R15B | 0.1208 | REG | −0.14 | <0.01 | 0.00 | 0.00 |
| 1 | 205575893 | ELK4 | 0.1527 | REG | 0.24 | 0.02 | 0.28 | 0.01 |
| 1 | 211668235 | SLC30A1 | 0.0033 | REG | −0.37 | <0.01 | 0.00 | 0.00 |
| 1 | 211668593 | SLC30A1 | 0.0842 | REG | 0.08 | <0.01 | 0.00 | 0.00 |
| 1 | 211755204 | SLC30A1 | 0.1301 | REG | 0.09 | <0.01 | 0.00 | 0.00 |
| 1 | 211830217 | SLC30A1 | 0.0567 | REG | 0.14 | <0.01 | 0.00 | 0.00 |
| 1 | 223857483 | CAPN2 | 0.0485 | REG | 0.10 | <0.01 | 0.21 | <0.01 |
| 1 | 223887395 | CAPN2 | 0.0066 | REG | 0.34 | <0.01 | 0.74 | <0.01 |
| 1 | 223889448 | CAPN2 | 0.0293 | REG | −0.19 | <0.01 | −0.40 | <0.01 |
| 1 | 223895544 | CAPN2 | 0.0033 | REG | −0.27 | <0.01 | −0.59 | <0.01 |
| 1 | 223943536 | CAPN2 | 0.1803 | REG | 0.07 | <0.01 | 0.15 | <0.01 |
| 1 | 223947081 | CAPN2 | 0.0082 | N-S | 0.00 | 0.00 | 0.00 | 0.00 |
| 1 | 223949314 | CAPN2 | 0.0066 | N-S | 0.00 | 0.00 | 0.00 | 0.00 |
| 1 | 223949936 | CAPN2 | 0.0017 | N-S | 0.00 | 0.00 | 0.00 | 0.00 |
| 1 | 223954080 | CAPN2 | 0.2110 | N-S | 0.00 | 0.00 | 0.00 | 0.00 |
| 1 | 223959891 | CAPN2 | 0.0033 | N-S | 0.00 | 0.00 | 0.00 | 0.00 |
| 1 | 229481321 | ACTA1 | 0.0049 | REG | 0.41 | <0.01 | 0.56 | <0.01 |
| 1 | 229572398 | ACTA1 | 0.0066 | REG | 0.46 | <0.01 | 0.64 | <0.01 |
| 1 | 229573774 | ACTA1 | 0.0098 | REG | 0.80 | 0.01 | 1.11 | 0.01 |
| 1 | 230379092 | GALNT2 | 0.0033 | N-S | 0.00 | 0.00 | 0.00 | 0.00 |
| 1 | 230386238 | GALNT2 | 0.0016 | N-S | 0.00 | 0.00 | 0.00 | 0.00 |
| 1 | 230415148 | GALNT2 | 0.1899 | N-S | 0.00 | 0.00 | 0.00 | 0.00 |
| 1 | 234736280 | IRF2BP2 | 0.0049 | REG | −0.20 | <0.01 | 0.00 | 0.00 |
| 1 | 234744480 | IRF2BP2 | 0.0597 | N-S | 0.00 | 0.00 | 0.00 | 0.00 |
| 1 | 234761797 | IRF2BP2 | 0.0049 | REG | −0.42 | <0.01 | 0.00 | 0.00 |
| 1 | 234763954 | IRF2BP2 | 0.0887 | REG | −0.08 | <0.01 | 0.00 | 0.00 |
| 1 | 236966848 | MTR | 0.0065 | N-S | 0.00 | 0.00 | 0.00 | 0.00 |
| 1 | 236980016 | MTR | 0.0016 | REG | −0.62 | <0.01 | 0.00 | 0.00 |
| 1 | 236987946 | MTR | 0.0033 | REG | 0.30 | <0.01 | 0.00 | 0.00 |
| 1 | 236990141 | MTR | 0.0264 | N-S | 0.00 | 0.00 | 0.00 | 0.00 |
| 1 | 236992526 | MTR | 0.0065 | N-S | 0.00 | 0.00 | 0.00 | 0.00 |
| 1 | 237006830 | MTR | 0.0033 | REG | −0.34 | <0.01 | 0.00 | 0.00 |
| 1 | 237012997 | MTR | 0.0255 | REG | 0.61 | 0.03 | 0.00 | 0.00 |
| 1 | 237015678 | MTR | 0.0033 | REG | 0.36 | <0.01 | 0.00 | 0.00 |
| 1 | 237019600 | MTR | 0.0017 | REG | 0.18 | <0.01 | 0.00 | 0.00 |
| 1 | 237048500 | MTR | 0.1874 | N-S | −0.10 | <0.01 | 0.00 | 0.00 |
| 1 | 237054504 | MTR | 0.0065 | N-S | −0.14 | <0.01 | 0.00 | 0.00 |
| 1 | 237058212 | MTR | 0.0238 | REG | −0.81 | 0.04 | 0.00 | 0.00 |
| 1 | 237058743 | MTR | 0.0099 | N-S | 0.00 | 0.00 | 0.00 | 0.00 |
| 1 | 237203236 | MTR | 0.0049 | REG | 0.37 | <0.01 | 0.00 | 0.00 |
| 1 | 241767660 | OPN3 | 0.0049 | N-S | 0.01 | <0.01 | 0.00 | 0.00 |
| 1 | 241767708 | OPN3 | 0.0050 | N-S | 0.00 | 0.00 | 0.00 | 0.00 |
| 1 | 241776313 | OPN3 | 0.0197 | REG | 0.27 | <0.01 | 0.00 | 0.00 |
| 1 | 241778561 | OPN3 | 0.0016 | REG | −0.56 | <0.01 | 0.00 | 0.00 |
| 1 | 241790148 | OPN3 | 0.2093 | REG | −0.37 | 0.05 | 0.00 | 0.00 |
| 1 | 241791117 | OPN3 | 0.1968 | REG | 0.27 | 0.03 | 0.00 | 0.00 |
| 1 | 241800958 | OPN3 | 0.0114 | REG | 0.64 | 0.01 | 0.00 | 0.00 |
| 3 | 4459755 | SUMF1 | 0.0033 | N-S | 0.00 | 0.00 | 0.00 | 0.00 |
| 3 | 4508742 | SUMF1 | 0.1856 | N-S | 0.17 | 0.01 | 0.22 | 0.01 |
| 3 | 4508871 | SUMF1 | 0.0049 | N-S | 0.02 | <0.01 | 0.03 | <0.01 |
| 3 | 4532469 | SUMF1 | 0.4902 | REG | 0.09 | 0.01 | 0.08 | <0.01 |
| 3 | 8812615 | RAD18 | 0.0033 | REG | 0.46 | <0.01 | 0.00 | 0.00 |
| 3 | 8816976 | RAD18 | 0.3325 | REG | −0.07 | <0.01 | 0.00 | 0.00 |
| 3 | 8936360 | RAD18 | 0.0082 | REG | 0.51 | <0.01 | 0.00 | 0.00 |
| 3 | 8955375 | RAD18 | 0.0033 | N-S | 0.00 | 0.00 | 0.00 | 0.00 |
| 3 | 8955389 | RAD18 | 0.3757 | N-S | 0.00 | 0.00 | 0.00 | 0.00 |
| 3 | 13569411 | FBLN2 | 0.0016 | REG | −0.61 | 0.01 | −0.57 | <0.01 |
| 3 | 13581314 | FBLN2 | 0.0017 | REG | 0.67 | 0.01 | 0.63 | <0.01 |
| 3 | 13591468 | FBLN2 | 0.0115 | REG | −0.54 | 0.01 | −0.51 | <0.01 |
| 3 | 13594608 | FBLN2 | 0.0555 | REG | −0.19 | 0.01 | −0.18 | <0.01 |
| 3 | 13651325 | FBLN2 | 0.0016 | REG | −0.67 | <0.01 | −0.62 | <0.01 |
| 3 | 13657525 | FBLN2 | 0.0191 | REG | 0.29 | 0.01 | 0.27 | <0.01 |
| 3 | 13682349 | FBLN2 | 0.0371 | REG | −0.25 | 0.01 | −0.23 | <0.01 |
| 3 | 15648008 | BTD | 0.0033 | REG | 0.49 | 0.01 | 0.32 | <0.01 |
| 3 | 15657226 | BTD | 0.0049 | REG | 0.36 | <0.01 | 0.23 | <0.01 |
| 3 | 15666325 | BTD | 0.0188 | REG | −0.36 | 0.01 | −0.24 | <0.01 |
| 3 | 15677019 | BTD | 0.0065 | N-S | 0.00 | 0.00 | 0.00 | 0.00 |
| 3 | 15677410 | BTD | 0.0130 | REG | 0.45 | 0.01 | 0.29 | <0.01 |
| 3 | 15681316 | BTD | 0.0016 | REG | 0.36 | <0.01 | 0.24 | <0.01 |
| 3 | 15685989 | BTD | 0.0017 | N-S | −0.66 | 0.01 | −0.59 | <0.01 |
| 3 | 15686243 | BTD | 0.0049 | N-S | 0.00 | 0.00 | 0.00 | 0.00 |
| 3 | 15686693 | BTD | 0.0213 | N-S | −0.63 | 0.01 | −0.56 | <0.01 |
| 3 | 33841065 | PDCD6IP | 0.4768 | REG | −0.27 | 0.04 | −0.26 | 0.02 |
| 3 | 33845186 | PDCD6IP | 0.0016 | REG | 0.52 | <0.01 | 0.51 | <0.01 |
| 3 | 33855359 | PDCD6IP | 0.0490 | REG | 0.14 | <0.01 | 0.14 | <0.01 |
| 3 | 33867279 | PDCD6IP | 0.4050 | REG | 0.16 | 0.01 | 0.15 | 0.01 |
| 3 | 33877626 | PDCD6IP | 0.4523 | N-S | 0.00 | 0.00 | 0.00 | 0.00 |
| 3 | 33893987 | PDCD6IP | 0.0664 | N-S | 0.00 | 0.00 | 0.00 | 0.00 |
| 3 | 33902658 | PDCD6IP | 0.0412 | REG | 0.12 | <0.01 | 0.12 | <0.01 |
| 3 | 33905566 | PDCD6IP | 0.3813 | N-S | 0.00 | 0.00 | 0.00 | 0.00 |
| 3 | 35676303 | PDCD6IP | 0.0441 | REG | −0.12 | <0.01 | −0.11 | <0.01 |
| 3 | 35679775 | PDCD6IP | 0.0016 | REG | 0.38 | <0.01 | 0.37 | <0.01 |
| 3 | 35680206 | PDCD6IP | 0.0082 | REG | 0.30 | <0.01 | 0.29 | <0.01 |
| 3 | 35681448 | PDCD6IP | 0.0065 | REG | 0.41 | <0.01 | 0.39 | <0.01 |
| 3 | 37034946 | MLH1 | 0.3107 | REG | −0.11 | 0.01 | 0.00 | 0.00 |
| 3 | 37039716 | MLH1 | 0.0049 | REG | −0.64 | <0.01 | 0.00 | 0.00 |
| 3 | 37045960 | MLH1 | 0.0033 | REG | −0.28 | <0.01 | 0.00 | 0.00 |
| 3 | 37048495 | MLH1 | 0.0016 | N-S | −0.50 | <0.01 | 0.00 | 0.00 |
| 3 | 37053568 | MLH1 | 0.1701 | N-S | 0.00 | 0.00 | 0.00 | 0.00 |
| 3 | 37061893 | MLH1 | 0.0033 | N-S | 0.00 | 0.00 | 0.00 | 0.00 |
| 3 | 37072537 | MLH1 | 0.0082 | REG | 0.33 | <0.01 | 0.00 | 0.00 |
| 3 | 37075084 | MLH1 | 0.0033 | REG | 0.36 | <0.01 | 0.00 | 0.00 |
| 3 | 37089615 | MLH1 | 0.0016 | REG | −0.49 | <0.01 | 0.00 | 0.00 |
| 3 | 37092025 | MLH1 | 0.0065 | N-S | −0.48 | 0.01 | 0.00 | 0.00 |
| 3 | 45987980 | CXCR6 | 0.0310 | N-S | 0.00 | 0.00 | 0.00 | 0.00 |
| 3 | 47455261 | SCAP | 0.0134 | REG | 0.00 | 0.00 | −0.32 | <0.01 |
| 3 | 47459679 | SCAP | 0.4565 | N-S | 0.00 | 0.00 | 0.00 | 0.00 |
| 3 | 47462171 | SCAP | 0.0016 | N-S | 0.00 | 0.00 | 0.00 | 0.00 |
| 3 | 47467805 | SCAP | 0.0033 | REG | 0.00 | 0.00 | −0.37 | <0.01 |
| 3 | 47519537 | SCAP | 0.0033 | REG | 0.00 | 0.00 | −0.38 | <0.01 |
| 3 | 47521574 | SCAP | 0.0896 | REG | 0.00 | 0.00 | 0.22 | 0.01 |
| 3 | 47912898 | MAP4 | 0.0049 | REG | 1.71 | 0.02 | 2.34 | 0.02 |
| 3 | 47913455 | MAP4 | 0.0049 | N-S | −5.46 | 0.36 | −8.70 | 0.44 |
| 3 | 47924216 | MAP4 | 0.0066 | REG | 1.35 | 0.06 | 1.84 | 0.05 |
| 3 | 47955326 | MAP4 | 0.0066 | REG | −1.93 | 0.03 | −2.63 | 0.03 |
| 3 | 47956424 | MAP4 | 0.3777 | N-S | −1.50 | 1.17 | −2.38 | 1.43 |
| 3 | 47957741 | MAP4 | 0.0016 | N-S | −5.08 | 0.24 | −8.10 | 0.30 |
| 3 | 47957996 | MAP4 | 0.0301 | N-S | −4.64 | 1.22 | −7.39 | 1.49 |
| 3 | 47958037 | MAP4 | 0.3420 | N-S | 0.00 | 0.00 | 0.00 | 0.00 |
| 3 | 47973345 | MAP4 | 0.0082 | REG | 2.14 | 0.06 | 2.92 | 0.05 |
| 3 | 48040283 | MAP4 | 0.0318 | N-S | −6.22 | 2.29 | −9.91 | 2.78 |
| 3 | 48040284 | MAP4 | 0.0131 | N-S | −6.95 | 0.91 | −11.07 | 1.11 |
| 3 | 48054461 | MAP4 | 0.1187 | REG | 0.46 | 0.03 | 0.63 | 0.03 |
| 3 | 48061725 | MAP4 | 0.0050 | REG | 1.79 | 0.03 | 2.44 | 0.03 |
| 3 | 48069438 | MAP4 | 0.0065 | REG | −1.78 | 0.03 | −2.43 | 0.03 |
| 3 | 48091219 | MAP4 | 0.0065 | REG | 2.54 | 0.03 | 3.46 | 0.02 |
| 3 | 50177068 | SEMA3F | 0.0065 | REG | 0.76 | <0.01 | 0.65 | <0.01 |
| 3 | 50185967 | SEMA3F | 0.0300 | REG | 0.25 | <0.01 | 0.21 | <0.01 |
| 3 | 50197098 | SEMA3F | 0.0115 | N-S | 0.09 | <0.01 | 0.10 | <0.01 |
| 3 | 50202745 | SEMA3F | 0.0114 | REG | 0.76 | <0.01 | 0.65 | <0.01 |
| 3 | 50222926 | SEMA3F | 0.2231 | N-S | 0.00 | 0.00 | 0.00 | 0.00 |
| 3 | 50361307 | TUSC2 | 0.0033 | REG | 0.50 | <0.01 | 0.90 | <0.01 |
| 3 | 56721540 | ARHGEF3 | 0.4892 | REG | 0.11 | 0.01 | 0.08 | <0.01 |
| 3 | 56771251 | ARHGEF3 | 0.4891 | N-S | 0.00 | 0.00 | 0.00 | 0.00 |
| 3 | 56771323 | ARHGEF3 | 0.0049 | N-S | −0.32 | <0.01 | −0.33 | <0.01 |
| 3 | 56852890 | ARHGEF3 | 0.0049 | REG | 0.57 | <0.01 | 0.42 | <0.01 |
| 3 | 56870810 | ARHGEF3 | 0.0033 | REG | 0.39 | <0.01 | 0.29 | <0.01 |
| 3 | 56890889 | ARHGEF3 | 0.0082 | REG | −0.70 | <0.01 | −0.52 | <0.01 |
| 3 | 56895493 | ARHGEF3 | 0.0316 | REG | 0.16 | <0.01 | 0.12 | <0.01 |
| 3 | 56905047 | ARHGEF3 | 0.0193 | REG | −0.29 | <0.01 | −0.22 | <0.01 |
| 3 | 56958677 | ARHGEF3 | 0.3210 | REG | 0.08 | <0.01 | 0.06 | <0.01 |
| 3 | 57047316 | ARHGEF3 | 0.0033 | REG | 0.65 | 0.01 | 0.48 | <0.01 |
| 3 | 57063499 | ARHGEF3 | 0.0034 | REG | −0.43 | <0.01 | −0.32 | <0.01 |
| 3 | 57550755 | ARF4 | 0.0033 | REG | −0.73 | <0.01 | 0.00 | 0.00 |
| 3 | 57551552 | ARF4 | 0.0033 | REG | −0.49 | <0.01 | 0.00 | 0.00 |
| 3 | 57586528 | ARF4 | 0.0033 | REG | −0.59 | <0.01 | 0.00 | 0.00 |
| 3 | 58089761 | FLNB | 0.0066 | N-S | 0.33 | <0.01 | 0.85 | <0.01 |
| 3 | 58090893 | FLNB | 0.0033 | N-S | 0.00 | 0.00 | 0.00 | 0.00 |
| 3 | 58097971 | FLNB | 0.0016 | N-S | 0.00 | 0.00 | 0.00 | 0.00 |
| 3 | 58109162 | FLNB | 0.4947 | N-S | 0.39 | 0.08 | 1.00 | 0.27 |
| 3 | 58109228 | FLNB | 0.0065 | N-S | 0.00 | 0.00 | 0.00 | 0.00 |
| 3 | 58118555 | FLNB | 0.4849 | N-S | 0.00 | 0.00 | 0.00 | 0.00 |
| 3 | 58132588 | FLNB | 0.0016 | N-S | 0.02 | <0.01 | 0.04 | <0.01 |
| 3 | 58134505 | FLNB | 0.0233 | N-S | 0.00 | 0.00 | 0.00 | 0.00 |
| 3 | 58139144 | FLNB | 0.0049 | N-S | 0.32 | <0.01 | 0.81 | <0.01 |
| 3 | 58145348 | FLNB | 0.0098 | N-S | 0.09 | <0.01 | 0.23 | <0.01 |
| 3 | 58159340 | FLNB | 0.0778 | REG | −0.10 | <0.01 | −0.19 | <0.01 |
| 3 | 58161774 | FLNB | 0.2362 | REG | −0.08 | <0.01 | −0.15 | <0.01 |
| 3 | 58181747 | DNASE1L3 | 0.3905 | REG | −0.07 | <0.01 | −0.10 | <0.01 |
| 3 | 58183636 | DNASE1L3 | 0.0456 | N-S | 0.36 | 0.01 | 0.67 | 0.02 |
| 3 | 58186719 | DNASE1L3 | 0.2050 | REG | −0.08 | <0.01 | −0.11 | <0.01 |
| 3 | 58187191 | DNASE1L3 | 0.0065 | REG | −0.34 | <0.01 | −0.46 | <0.01 |
| 3 | 58190853 | DNASE1L3 | 0.0049 | REG | 0.29 | <0.01 | 0.39 | <0.01 |
| 3 | 58191230 | DNASE1L3 | 0.0681 | N-S | 0.00 | 0.00 | 0.00 | 0.00 |
| 3 | 58192585 | DNASE1L3 | 0.2637 | REG | 0.04 | <0.01 | 0.05 | <0.01 |
| 3 | 58197373 | DNASE1L3 | 0.0311 | REG | 0.12 | <0.01 | 0.16 | <0.01 |
| 3 | 71816586 | PROK2 | 0.0033 | REG | 0.37 | <0.01 | 0.00 | 0.00 |
| 3 | 71824151 | PROK2 | 0.4168 | REG | −0.07 | <0.01 | 0.00 | 0.00 |
| 3 | 71829242 | PROK2 | 0.1961 | REG | 0.33 | 0.04 | 0.00 | 0.00 |
| 3 | 71829786 | PROK2 | 0.1460 | REG | −0.10 | <0.01 | 0.00 | 0.00 |
| 3 | 71836209 | PROK2 | 0.2149 | REG | 0.07 | <0.01 | 0.00 | 0.00 |
| 3 | 71837235 | PROK2 | 0.4492 | REG | −0.03 | <0.01 | 0.00 | 0.00 |
| 3 | 72115343 | PROK2 | 0.0147 | REG | −0.24 | <0.01 | 0.00 | 0.00 |
| 3 | 72136524 | PROK2 | 0.0049 | REG | 0.34 | <0.01 | 0.00 | 0.00 |
| 3 | 72422986 | RYBP | 0.0067 | REG | 0.73 | 0.01 | 0.00 | 0.00 |
| 3 | 72447890 | RYBP | 0.4517 | REG | −0.27 | 0.04 | 0.00 | 0.00 |
| 3 | 72451817 | RYBP | 0.0299 | REG | 0.14 | <0.01 | 0.00 | 0.00 |
| 3 | 72494085 | RYBP | 0.2405 | REG | 0.09 | <0.01 | 0.00 | 0.00 |
| 3 | 72494258 | RYBP | 0.0033 | REG | 0.49 | <0.01 | 0.00 | 0.00 |
| 3 | 118930386 | B4GALT4 | 0.1491 | REG | −0.12 | <0.01 | −0.13 | <0.01 |
| 3 | 118945796 | B4GALT4 | 0.3483 | N-S | 0.00 | 0.00 | 0.00 | 0.00 |
| 3 | 118955611 | B4GALT4 | 0.0511 | REG | 0.19 | <0.01 | 0.19 | <0.01 |
| 3 | 123170592 | PTPLB | 0.0050 | REG | 0.41 | <0.01 | 0.49 | <0.01 |
| 3 | 123215503 | PTPLB | 0.0033 | REG | −0.29 | <0.01 | −0.35 | <0.01 |
| 3 | 123226739 | PTPLB | 0.0663 | REG | −0.14 | <0.01 | −0.17 | <0.01 |
| 3 | 123252747 | PTPLB | 0.0049 | REG | 0.65 | <0.01 | 0.79 | <0.01 |
| 3 | 124621412 | MUC13 | 0.0116 | REG | 0.67 | 0.01 | 0.00 | 0.00 |
| 3 | 124627007 | MUC13 | 0.0033 | N-S | −0.05 | <0.01 | 0.00 | 0.00 |
| 3 | 124627024 | MUC13 | 0.2056 | N-S | 0.00 | 0.00 | 0.00 | 0.00 |
| 3 | 124631855 | MUC13 | 0.0033 | REG | 0.92 | 0.01 | 0.00 | 0.00 |
| 3 | 124633261 | MUC13 | 0.0134 | REG | 0.74 | 0.01 | 0.00 | 0.00 |
| 3 | 124641476 | MUC13 | 0.1303 | REG | 0.15 | 0.01 | 0.00 | 0.00 |
| 3 | 124646594 | MUC13 | 0.4237 | N-S | 0.00 | 0.00 | 0.00 | 0.00 |
| 3 | 124646693 | MUC13 | 0.0116 | N-S | −0.11 | <0.01 | 0.00 | 0.00 |
| 3 | 124646837 | MUC13 | 0.1786 | N-S | −0.09 | <0.01 | 0.00 | 0.00 |
| 3 | 124680477 | MUC13 | 0.0066 | REG | 0.38 | 0.01 | 0.00 | 0.00 |
| 3 | 127394820 | ABTB1 | 0.0880 | REG | −0.29 | 0.02 | −0.58 | 0.03 |
| 3 | 127397553 | ABTB1 | 0.0894 | REG | 0.50 | 0.05 | 0.99 | 0.10 |
| 3 | 135713654 | PPP2R3A | 0.0049 | REG | 0.43 | <0.01 | 0.28 | <0.01 |
| 3 | 135714537 | PPP2R3A | 0.0049 | REG | 0.46 | <0.01 | 0.30 | <0.01 |
| 3 | 135720539 | PPP2R3A | 0.0016 | N-S | 0.00 | 0.00 | 0.00 | 0.00 |
| 3 | 135720540 | PPP2R3A | 0.1173 | N-S | 0.01 | <0.01 | 0.01 | <0.01 |
| 3 | 135720851 | PPP2R3A | 0.2997 | N-S | 0.00 | 0.00 | 0.00 | 0.00 |
| 3 | 135721781 | PPP2R3A | 0.0398 | N-S | 0.00 | 0.00 | 0.00 | 0.00 |
| 3 | 135722030 | PPP2R3A | 0.0016 | N-S | 0.00 | 0.00 | 0.00 | 0.00 |
| 3 | 135722264 | PPP2R3A | 0.1270 | N-S | 0.00 | 0.00 | 0.00 | 0.00 |
| 3 | 135728750 | PPP2R3A | 0.0016 | REG | 0.68 | <0.01 | 0.44 | <0.01 |
| 3 | 135745762 | PPP2R3A | 0.0082 | N-S | 0.00 | 0.00 | 0.00 | 0.00 |
| 3 | 135745810 | PPP2R3A | 0.0065 | N-S | 0.00 | 0.00 | 0.00 | 0.00 |
| 3 | 135745816 | PPP2R3A | 0.0016 | N-S | 0.62 | <0.01 | 0.56 | <0.01 |
| 3 | 135745911 | PPP2R3A | 0.0794 | N-S | 0.00 | 0.00 | 0.00 | 0.00 |
| 3 | 135789360 | PPP2R3A | 0.0033 | N-S | 0.53 | <0.01 | 0.47 | <0.01 |
| 3 | 135794793 | PPP2R3A | 0.0033 | REG | 1.06 | 0.03 | 0.69 | 0.01 |
| 3 | 135838819 | PPP2R3A | 0.0066 | REG | 0.39 | <0.01 | 0.25 | <0.01 |
| 3 | 135845487 | PPP2R3A | 0.0049 | REG | −0.55 | <0.01 | −0.36 | <0.01 |
| 3 | 135865869 | PPP2R3A | 0.0049 | REG | 0.59 | 0.01 | 0.38 | <0.01 |
| 3 | 139276557 | NMNAT3 | 0.0196 | REG | −0.19 | <0.01 | −0.25 | <0.01 |
| 3 | 139280101 | NMNAT3 | 0.0049 | N-S | −0.29 | <0.01 | −0.53 | <0.01 |
| 3 | 139310157 | NMNAT3 | 0.0082 | REG | −0.41 | <0.01 | −0.55 | <0.01 |
| 3 | 139323752 | NMNAT3 | 0.0892 | REG | −0.11 | <0.01 | −0.15 | <0.01 |
| 3 | 139338945 | NMNAT3 | 0.0033 | REG | 0.95 | 0.01 | 1.27 | 0.01 |
| 3 | 139377321 | NMNAT3 | 0.2599 | REG | −0.07 | <0.01 | −0.10 | <0.01 |
| 3 | 139387692 | NMNAT3 | 0.0082 | REG | 0.27 | <0.01 | 0.36 | <0.01 |
| 3 | 139388094 | NMNAT3 | 0.0049 | REG | −0.36 | <0.01 | −0.48 | <0.01 |
| 3 | 139393365 | NMNAT3 | 0.2925 | REG | 0.16 | 0.01 | 0.22 | 0.01 |
| 3 | 139395691 | NMNAT3 | 0.0247 | REG | −0.23 | <0.01 | −0.30 | <0.01 |
| 3 | 141040055 | ZBTB38 | 0.2010 | REG | −0.12 | 0.01 | −0.18 | 0.01 |
| 3 | 141040674 | ZBTB38 | 0.0016 | REG | 0.20 | <0.01 | 0.29 | <0.01 |
| 3 | 141058687 | ZBTB38 | 0.2560 | REG | 0.07 | <0.01 | 0.11 | <0.01 |
| 3 | 141062635 | ZBTB38 | 0.0065 | REG | 0.30 | <0.01 | 0.44 | <0.01 |
| 3 | 141086120 | ZBTB38 | 0.0033 | REG | −0.31 | <0.01 | −0.47 | <0.01 |
| 3 | 141093285 | ZBTB38 | 0.4363 | REG | −0.03 | <0.01 | −0.05 | <0.01 |
| 3 | 141107611 | ZBTB38 | 0.0016 | REG | 0.21 | <0.01 | 0.31 | <0.01 |
| 3 | 141160882 | ZBTB38 | 0.2108 | REG | −0.24 | 0.02 | −0.36 | 0.02 |
| 3 | 141162128 | ZBTB38 | 0.2109 | N-S | −0.29 | 0.03 | −0.60 | 0.06 |
| 3 | 141162185 | ZBTB38 | 0.2460 | N-S | 0.00 | 0.00 | −0.01 | <0.01 |
| 3 | 141163074 | ZBTB38 | 0.0049 | N-S | 0.00 | 0.00 | −0.01 | <0.01 |
| 3 | 141650812 | TFDP2 | 0.0049 | REG | 0.00 | 0.00 | −0.82 | <0.01 |
| 3 | 141693906 | TFDP2 | 0.0033 | REG | 0.00 | 0.00 | −0.37 | <0.01 |
| 3 | 141743448 | TFDP2 | 0.0033 | REG | 0.00 | 0.00 | −0.36 | <0.01 |
| 3 | 141754451 | TFDP2 | 0.0049 | REG | 0.00 | 0.00 | −0.40 | <0.01 |
| 3 | 141781034 | TFDP2 | 0.0264 | REG | 0.00 | 0.00 | 0.15 | <0.01 |
| 3 | 141803191 | TFDP2 | 0.0033 | REG | 0.00 | 0.00 | −0.43 | <0.01 |
| 3 | 150180263 | SERP1 | 0.0367 | REG | −0.16 | <0.01 | 0.00 | 0.00 |
| 3 | 150258357 | SERP1 | 0.0519 | REG | 0.09 | <0.01 | 0.00 | 0.00 |
| 3 | 150262513 | SERP1 | 0.3467 | REG | 0.05 | <0.01 | 0.00 | 0.00 |
| 3 | 169717234 | GPR160 | 0.1888 | REG | 0.24 | 0.02 | 0.00 | 0.00 |
| 3 | 169717820 | GPR160 | 0.1698 | REG | −0.18 | 0.01 | 0.00 | 0.00 |
| 3 | 169752951 | GPR160 | 0.4141 | REG | −0.09 | <0.01 | 0.00 | 0.00 |
| 3 | 169757762 | GPR160 | 0.4237 | REG | −0.08 | <0.01 | 0.00 | 0.00 |
| 3 | 169781050 | GPR160 | 0.0066 | REG | −0.17 | <0.01 | 0.00 | 0.00 |
| 3 | 169801800 | GPR160 | 0.0065 | N-S | 0.00 | 0.00 | 0.00 | 0.00 |
| 3 | 169801953 | GPR160 | 0.0016 | N-S | 0.00 | 0.00 | 0.00 | 0.00 |
| 3 | 184553064 | VPS8 | 0.0016 | REG | 0.54 | <0.01 | 0.00 | 0.00 |
| 3 | 184562138 | VPS8 | 0.0033 | REG | 1.05 | 0.01 | 0.00 | 0.00 |
| 3 | 184628217 | VPS8 | 0.0236 | REG | −0.24 | <0.01 | 0.00 | 0.00 |
| 3 | 184639928 | VPS8 | 0.0049 | REG | 0.83 | 0.01 | 0.00 | 0.00 |
| 3 | 184646391 | VPS8 | 0.0082 | REG | 0.51 | <0.01 | 0.00 | 0.00 |
| 3 | 184693247 | VPS8 | 0.0033 | REG | −0.54 | <0.01 | 0.00 | 0.00 |
| 3 | 196561319 | PAK2 | 0.2978 | REG | −0.09 | <0.01 | −0.14 | 0.01 |
| 3 | 196561545 | PAK2 | 0.0016 | REG | 0.23 | <0.01 | 0.36 | <0.01 |
| 3 | 196561604 | PAK2 | 0.1117 | REG | −0.16 | 0.01 | −0.25 | 0.01 |
| 3 | 196591355 | PAK2 | 0.0442 | REG | 0.07 | <0.01 | 0.11 | <0.01 |
| 3 | 196597635 | SENP5 | 0.0017 | REG | 0.00 | 0.00 | −0.53 | <0.01 |
| 3 | 196603413 | SENP5 | 0.0033 | REG | 0.00 | 0.00 | 0.40 | <0.01 |
| 3 | 196612300 | SENP5 | 0.0065 | N-S | 0.00 | 0.00 | 0.00 | 0.00 |
| 3 | 196613072 | SENP5 | 0.0115 | N-S | 0.00 | 0.00 | −0.06 | <0.01 |
| 3 | 196627915 | SENP5 | 0.0098 | REG | 0.00 | 0.00 | 0.43 | <0.01 |
| 3 | 196645040 | SENP5 | 0.0131 | REG | 0.00 | 0.00 | −0.32 | <0.01 |
| 3 | 196669950 | LOC152217 | 0.0788 | REG | 0.11 | <0.01 | 0.13 | <0.01 |
| 5 | 7870973 | MTRR | 0.2305 | N-S | −0.38 | 0.06 | 0.00 | 0.00 |
| 5 | 7878179 | MTRR | 0.2007 | N-S | 0.00 | 0.00 | 0.00 | 0.00 |
| 5 | 7878244 | MTRR | 0.0068 | N-S | −0.06 | <0.01 | 0.00 | 0.00 |
| 5 | 7878424 | MTRR | 0.0689 | N-S | 0.00 | 0.00 | 0.00 | 0.00 |
| 5 | 7885907 | MTRR | 0.0099 | N-S | −0.48 | 0.01 | 0.00 | 0.00 |
| 5 | 7885959 | MTRR | 0.3791 | N-S | 0.00 | 0.00 | 0.00 | 0.00 |
| 5 | 7889304 | MTRR | 0.1248 | N-S | −0.50 | 0.07 | 0.00 | 0.00 |
| 5 | 7891506 | MTRR | 0.1230 | N-S | −0.46 | 0.06 | 0.00 | 0.00 |
| 5 | 7893121 | MTRR | 0.2803 | REG | −0.36 | 0.07 | 0.00 | 0.00 |
| 5 | 7897191 | MTRR | 0.3660 | N-S | −0.44 | 0.10 | 0.00 | 0.00 |
| 5 | 39117115 | FYB | 0.0082 | REG | 0.00 | 0.00 | 0.51 | <0.01 |
| 5 | 39136870 | FYB | 0.0033 | REG | 0.00 | 0.00 | −0.58 | <0.01 |
| 5 | 39177446 | FYB | 0.0098 | REG | 0.00 | 0.00 | −0.57 | <0.01 |
| 5 | 39179447 | FYB | 0.0033 | REG | 0.00 | 0.00 | −0.80 | <0.01 |
| 5 | 39200154 | FYB | 0.4276 | REG | 0.00 | 0.00 | 0.07 | <0.01 |
| 5 | 39235409 | FYB | 0.0033 | REG | 0.00 | 0.00 | −0.45 | <0.01 |
| 5 | 39281833 | FYB | 0.0082 | REG | 0.00 | 0.00 | 0.51 | <0.01 |
| 5 | 88016008 | MEF2C | 0.4227 | REG | 0.06 | <0.01 | 0.08 | <0.01 |
| 5 | 88029517 | MEF2C | 0.0114 | REG | 0.52 | <0.01 | 0.69 | <0.01 |
| 5 | 88053321 | MEF2C | 0.0049 | REG | 0.25 | <0.01 | 0.34 | <0.01 |
| 5 | 88079168 | MEF2C | 0.0049 | REG | 0.31 | <0.01 | 0.41 | <0.01 |
| 5 | 88143749 | MEF2C | 0.0838 | REG | −0.07 | <0.01 | −0.10 | <0.01 |
| 5 | 88159249 | MEF2C | 0.0033 | REG | −0.35 | <0.01 | −0.47 | <0.01 |
| 5 | 89309032 | MEF2C | 0.0049 | REG | 0.35 | <0.01 | 0.47 | <0.01 |
| 5 | 96259174 | LNPEP | 0.0455 | REG | 0.00 | 0.00 | 0.20 | <0.01 |
| 5 | 96275550 | LNPEP | 0.0133 | REG | 0.00 | 0.00 | −0.41 | <0.01 |
| 5 | 96278948 | LNPEP | 0.2488 | REG | 0.00 | 0.00 | −0.12 | <0.01 |
| 5 | 96301390 | LNPEP | 0.0033 | REG | 0.00 | 0.00 | −0.48 | <0.01 |
| 5 | 96315349 | LNPEP | 0.0062 | N-S | 0.00 | 0.00 | 0.00 | 0.00 |
| 5 | 96322360 | LNPEP | 0.0353 | N-S | 0.00 | 0.00 | 0.00 | 0.00 |
| 5 | 96329622 | LNPEP | 0.0066 | N-S | 0.00 | 0.00 | 0.00 | 0.00 |
| 5 | 96333460 | LNPEP | 0.0065 | REG | 0.00 | 0.00 | 0.88 | <0.01 |
| 5 | 96341912 | LNPEP | 0.0065 | N-S | 0.00 | 0.00 | 0.00 | 0.00 |
| 5 | 96350710 | LNPEP | 0.1897 | N-S | 0.00 | 0.00 | −0.16 | 0.01 |
| 5 | 96351110 | LNPEP | 0.0049 | REG | 0.00 | 0.00 | 0.76 | <0.01 |
| 5 | 96363459 | LNPEP | 0.0678 | N-S | 0.00 | 0.00 | 0.00 | 0.00 |
| 5 | 112934024 | KCNN2 | 0.0082 | REG | 0.00 | 0.00 | 0.58 | <0.01 |
| 5 | 113700560 | KCNN2 | 0.0599 | REG | 0.00 | 0.00 | −0.18 | <0.01 |
| 5 | 113703660 | KCNN2 | 0.0033 | REG | 0.00 | 0.00 | −0.33 | <0.01 |
| 5 | 113748550 | KCNN2 | 0.0016 | REG | 0.00 | 0.00 | −0.42 | <0.01 |
| 5 | 113763861 | KCNN2 | 0.0016 | REG | 0.00 | 0.00 | 0.91 | <0.01 |
| 5 | 113776559 | KCNN2 | 0.0284 | REG | 0.00 | 0.00 | 0.26 | <0.01 |
| 5 | 113781554 | KCNN2 | 0.0065 | REG | 0.00 | 0.00 | 0.57 | <0.01 |
| 5 | 113783989 | KCNN2 | 0.1250 | REG | 0.00 | 0.00 | 0.13 | <0.01 |
| 5 | 113808740 | KCNN2 | 0.0033 | REG | 0.00 | 0.00 | −1.33 | 0.02 |
| 5 | 113885527 | KCNN2 | 0.0065 | REG | 0.00 | 0.00 | 0.61 | <0.01 |
| 5 | 113939493 | KCNN2 | 0.0249 | REG | 0.00 | 0.00 | −0.23 | <0.01 |
| 5 | 113950430 | KCNN2 | 0.0017 | REG | 0.00 | 0.00 | 0.48 | <0.01 |
| 5 | 118608839 | TNFAIP8 | 0.1796 | REG | −0.13 | 0.01 | −0.08 | <0.01 |
| 5 | 118623286 | TNFAIP8 | 0.0016 | REG | 1.03 | 0.01 | 0.65 | <0.01 |
| 5 | 118627589 | TNFAIP8 | 0.0365 | REG | 0.17 | <0.01 | 0.11 | <0.01 |
| 5 | 118640376 | TNFAIP8 | 0.0066 | REG | −0.33 | <0.01 | −0.21 | <0.01 |
| 5 | 118641635 | TNFAIP8 | 0.0065 | REG | 0.82 | <0.01 | 0.52 | <0.01 |
| 5 | 118643714 | TNFAIP8 | 0.0483 | REG | −0.22 | <0.01 | −0.14 | <0.01 |
| 5 | 118670786 | TNFAIP8 | 0.0883 | REG | −0.14 | <0.01 | −0.09 | <0.01 |
| 5 | 118675901 | TNFAIP8 | 0.3221 | REG | −0.10 | 0.01 | −0.06 | <0.01 |
| 5 | 118695797 | TNFAIP8 | 0.0082 | REG | −0.41 | <0.01 | −0.26 | <0.01 |
| 5 | 118700232 | TNFAIP8 | 0.0955 | REG | −0.12 | <0.01 | −0.08 | <0.01 |
| 5 | 132168802 | GDF9 | 0.0033 | REG | −0.81 | <0.01 | −1.33 | 0.01 |
| 5 | 132197442 | GDF9 | 0.0016 | N-S | 0.50 | <0.01 | 1.13 | 0.01 |
| 5 | 134595331 | H2AFY | 0.0033 | REG | 0.46 | <0.01 | 0.00 | 0.00 |
| 5 | 134635680 | H2AFY | 0.0245 | REG | −0.31 | <0.01 | 0.00 | 0.00 |
| 5 | 134694413 | H2AFY | 0.0065 | REG | −0.41 | <0.01 | 0.00 | 0.00 |
| 5 | 134708309 | H2AFY | 0.0082 | REG | −0.32 | <0.01 | 0.00 | 0.00 |
| 5 | 134729522 | H2AFY | 0.1862 | REG | 0.11 | <0.01 | 0.00 | 0.00 |
| 5 | 134738215 | H2AFY | 0.0066 | REG | −0.90 | 0.01 | 0.00 | 0.00 |
| 5 | 137532394 | CDC23 | 0.0033 | REG | 0.64 | <0.01 | 0.52 | <0.01 |
| 5 | 137550054 | CDC23 | 0.3822 | REG | 0.18 | 0.02 | 0.15 | 0.01 |
| 5 | 149820710 | RPS14 | 0.0065 | REG | 0.47 | <0.01 | 0.51 | <0.01 |
| 5 | 149827494 | RPS14 | 0.3084 | REG | 0.11 | 0.01 | 0.12 | <0.01 |
| 5 | 149873608 | RPS14 | 0.0148 | REG | 0.36 | <0.01 | 0.39 | <0.01 |
| 5 | 169676011 | LCP2 | 0.0033 | REG | 0.00 | 0.00 | 0.41 | <0.01 |
| 5 | 169725750 | LCP2 | 0.4348 | REG | 0.00 | 0.00 | 0.06 | <0.01 |
| 5 | 169754212 | LCP2 | 0.0033 | REG | 0.00 | 0.00 | −0.38 | <0.01 |
| 5 | 170882519 | FGF18 | 0.0050 | REG | −0.22 | <0.01 | 0.00 | 0.00 |
| 5 | 170886327 | FGF18 | 0.0049 | REG | −0.52 | <0.01 | 0.00 | 0.00 |
| 5 | 171284337 | FGF18 | 0.0098 | REG | 0.59 | <0.01 | 0.00 | 0.00 |
| 5 | 171465473 | STK10 | 0.0180 | REG | 0.00 | 0.00 | −0.23 | <0.01 |
| 5 | 171475774 | STK10 | 0.0132 | REG | 0.00 | 0.00 | 0.21 | <0.01 |
| 5 | 171509416 | STK10 | 0.0033 | N-S | 0.00 | 0.00 | −0.62 | <0.01 |
| 5 | 171509487 | STK10 | 0.0016 | N-S | 0.00 | 0.00 | 0.00 | 0.00 |
| 5 | 171520790 | STK10 | 0.0460 | N-S | 0.00 | 0.00 | 0.00 | 0.00 |
| 5 | 171520792 | STK10 | 0.0016 | N-S | 0.00 | 0.00 | 0.00 | 0.00 |
| 5 | 171528330 | STK10 | 0.0033 | REG | 0.00 | 0.00 | −0.75 | <0.01 |
| 5 | 171529240 | STK10 | 0.0066 | REG | 0.00 | 0.00 | 0.73 | <0.01 |
| 5 | 171532724 | STK10 | 0.0016 | N-S | 0.00 | 0.00 | −0.10 | <0.01 |
| 5 | 171534307 | STK10 | 0.0049 | REG | 0.00 | 0.00 | −0.36 | <0.01 |
| 5 | 171536092 | STK10 | 0.1360 | REG | 0.00 | 0.00 | 0.09 | <0.01 |
| 5 | 171574822 | STK10 | 0.2997 | REG | 0.00 | 0.00 | 0.06 | <0.01 |
| 5 | 171591925 | STK10 | 0.0016 | REG | 0.00 | 0.00 | −0.46 | <0.01 |
| 5 | 172196752 | DUSP1 | 0.0082 | N-S | 0.00 | 0.00 | 0.00 | 0.00 |
| 5 | 174908609 | SFXN1 | 0.0329 | REG | −0.25 | 0.01 | 0.00 | 0.00 |
| 5 | 174909860 | SFXN1 | 0.0016 | REG | 0.41 | <0.01 | 0.00 | 0.00 |
| 5 | 174910624 | SFXN1 | 0.4130 | REG | −0.05 | <0.01 | 0.00 | 0.00 |
| 5 | 174935529 | SFXN1 | 0.0033 | REG | 0.80 | <0.01 | 0.00 | 0.00 |
| 5 | 174936589 | SFXN1 | 0.0049 | REG | 0.34 | <0.01 | 0.00 | 0.00 |
| 5 | 174959880 | SFXN1 | 0.3031 | REG | 0.10 | 0.01 | 0.00 | 0.00 |
| 5 | 176526701 | NSD1 | 0.0016 | REG | −0.33 | <0.01 | −0.29 | <0.01 |
| 5 | 176581319 | NSD1 | 0.0199 | REG | 0.16 | <0.01 | 0.14 | <0.01 |
| 5 | 176590538 | NSD1 | 0.0016 | REG | 0.51 | <0.01 | 0.44 | <0.01 |
| 5 | 176599645 | NSD1 | 0.0033 | REG | 0.90 | <0.01 | 0.79 | <0.01 |
| 5 | 176603244 | NSD1 | 0.0179 | REG | 0.38 | 0.01 | 0.33 | <0.01 |
| 5 | 176608728 | NSD1 | 0.0033 | REG | −0.76 | 0.01 | −0.66 | <0.01 |
| 5 | 176637240 | NSD1 | 0.1779 | N-S | 0.00 | 0.00 | 0.00 | 0.00 |
| 5 | 176637471 | NSD1 | 0.0131 | N-S | 0.00 | 0.00 | 0.00 | 0.00 |
| 5 | 176637576 | NSD1 | 0.1889 | N-S | 0.39 | 0.05 | 0.47 | 0.04 |
| 5 | 176638209 | NSD1 | 0.0016 | N-S | 0.58 | <0.01 | 0.69 | <0.01 |
| 5 | 176638506 | NSD1 | 0.0164 | N-S | 0.00 | 0.00 | 0.00 | 0.00 |
| 5 | 176639122 | NSD1 | 0.0049 | N-S | 0.00 | 0.00 | 0.00 | 0.00 |
| 5 | 176721119 | NSD1 | 0.0282 | N-S | 0.01 | <0.01 | 0.01 | <0.01 |
| 5 | 176721151 | NSD1 | 0.0282 | N-S | 0.00 | 0.00 | 0.00 | 0.00 |
| 5 | 176722005 | NSD1 | 0.0148 | N-S | 0.08 | <0.01 | 0.10 | <0.01 |
| 7 | 5555356 | ACTB | 0.0016 | REG | −0.30 | <0.01 | −0.32 | <0.01 |
| 7 | 5564272 | ACTB | 0.0016 | REG | 0.44 | <0.01 | 0.47 | <0.01 |
| 7 | 5628886 | ACTB | 0.0033 | REG | 0.35 | <0.01 | 0.37 | <0.01 |
| 7 | 6419049 | RAC1 | 0.3411 | REG | 0.00 | 0.00 | 0.30 | 0.02 |
| 7 | 6427962 | RAC1 | 0.0049 | REG | 0.00 | 0.00 | 0.55 | <0.01 |
| 7 | 6428410 | RAC1 | 0.0133 | REG | 0.00 | 0.00 | −0.17 | <0.01 |
| 7 | 6438217 | RAC1 | 0.0414 | REG | 0.00 | 0.00 | 0.13 | <0.01 |
| 7 | 6447041 | RAC1 | 0.3816 | REG | 0.00 | 0.00 | −0.16 | 0.01 |
| 7 | 27211071 | HOXA10 | 0.0016 | REG | 0.32 | <0.01 | 0.29 | <0.01 |
| 7 | 27211548 | HOXA10 | 0.0196 | REG | −0.21 | <0.01 | −0.19 | <0.01 |
| 7 | 43629820 | STK17A | 0.0465 | REG | 0.17 | <0.01 | 0.14 | <0.01 |
| 7 | 43647506 | STK17A | 0.0081 | REG | 0.61 | <0.01 | 0.53 | <0.01 |
| 7 | 43659302 | STK17A | 0.0016 | N-S | 0.53 | <0.01 | 0.64 | <0.01 |
| 7 | 43661708 | STK17A | 0.0016 | REG | −0.52 | <0.01 | −0.46 | <0.01 |
| 7 | 43664280 | STK17A | 0.1353 | N-S | 0.00 | 0.00 | 0.00 | 0.00 |
| 7 | 44244834 | YKT6 | 0.0016 | REG | −0.33 | <0.01 | −0.50 | <0.01 |
| 7 | 44245987 | YKT6 | 0.0016 | N-S | 0.30 | <0.01 | 0.63 | <0.01 |
| 7 | 44251199 | YKT6 | 0.0099 | REG | −0.19 | <0.01 | −0.28 | <0.01 |
| 7 | 44253381 | YKT6 | 0.0065 | REG | −0.25 | <0.01 | −0.38 | <0.01 |
| 7 | 56162172 | CHCHD2 | 0.3909 | REG | 0.00 | 0.00 | −0.09 | <0.01 |
| 7 | 56162336 | CHCHD2 | 0.0017 | REG | 0.00 | 0.00 | −0.48 | <0.01 |
| 7 | 56171760 | CHCHD2 | 0.1153 | REG | 0.00 | 0.00 | 0.24 | 0.01 |
| 7 | 66453476 | SBDS | 0.0377 | N-S | 0.00 | 0.00 | 0.00 | 0.00 |
| 7 | 66458306 | SBDS | 0.0099 | REG | 0.18 | <0.01 | 0.00 | 0.00 |
| 7 | 73822336 | GTF2IRD1 | 0.0033 | REG | 0.00 | 0.00 | −2.41 | 0.03 |
| 7 | 73878915 | GTF2IRD1 | 0.0098 | REG | 0.00 | 0.00 | 0.56 | <0.01 |
| 7 | 73880548 | GTF2IRD1 | 0.0924 | REG | 0.00 | 0.00 | −0.96 | 0.09 |
| 7 | 73885869 | GTF2IRD1 | 0.0803 | REG | 0.00 | 0.00 | 0.79 | 0.06 |
| 7 | 73907541 | GTF2IRD1 | 0.0033 | REG | 0.00 | 0.00 | 0.54 | <0.01 |
| 7 | 73924488 | GTF2IRD1 | 0.0883 | REG | 0.00 | 0.00 | −0.19 | <0.01 |
| 7 | 73935262 | GTF2IRD1 | 0.1883 | REG | 0.00 | 0.00 | 0.72 | 0.10 |
| 7 | 73957401 | GTF2IRD1 | 0.1738 | REG | 0.00 | 0.00 | −0.61 | 0.06 |
| 7 | 73969541 | GTF2IRD1 | 0.3000 | N-S | 0.00 | 0.00 | 0.00 | 0.00 |
| 7 | 73991012 | GTF2IRD1 | 0.0033 | REG | 0.00 | 0.00 | −1.09 | <0.01 |
| 7 | 74001059 | GTF2IRD1 | 0.0082 | REG | 0.00 | 0.00 | −0.73 | <0.01 |
| 7 | 75442723 | CCL24 | 0.0148 | N-S | −0.66 | 0.01 | −1.12 | 0.02 |
| 7 | 75442730 | CCL24 | 0.4579 | N-S | 0.00 | 0.00 | 0.00 | 0.00 |
| 7 | 75446655 | CCL24 | 0.0033 | REG | −0.67 | 0.01 | −0.82 | 0.01 |
| 7 | 76111938 | DTX2 | 0.0049 | N-S | 0.00 | 0.00 | 0.00 | 0.00 |
| 7 | 76112702 | DTX2 | 0.4486 | REG | 0.06 | <0.01 | 0.07 | <0.01 |
| 7 | 76131684 | DTX2 | 0.0098 | N-S | 0.00 | 0.00 | 0.00 | 0.00 |
| 7 | 76131695 | DTX2 | 0.3293 | REG | −0.07 | <0.01 | −0.08 | <0.01 |
| 7 | 77510797 | PHTF2 | 0.0016 | REG | −0.48 | <0.01 | 0.00 | 0.00 |
| 7 | 77616921 | PHTF2 | 0.0118 | REG | −0.29 | <0.01 | 0.00 | 0.00 |
| 7 | 77620262 | PHTF2 | 0.0049 | REG | 0.46 | <0.01 | 0.00 | 0.00 |
| 7 | 77626255 | PHTF2 | 0.0016 | REG | −0.52 | <0.01 | 0.00 | 0.00 |
| 7 | 100172858 | LRCH4 | 0.1451 | N-S | 0.00 | 0.00 | 0.09 | <0.01 |
| 7 | 100174752 | LRCH4 | 0.0016 | N-S | 0.00 | 0.00 | 0.00 | 0.00 |
| 7 | 100175010 | LRCH4 | 0.0114 | N-S | 0.00 | 0.00 | 0.60 | 0.01 |
| 7 | 100176317 | LRCH4 | 0.0033 | N-S | 0.00 | 0.00 | 0.07 | <0.01 |
| 7 | 100180896 | LRCH4 | 0.0496 | REG | 0.00 | 0.00 | 0.09 | <0.01 |
| 7 | 100183609 | LRCH4 | 0.0016 | N-S | 0.00 | 0.00 | 0.56 | <0.01 |
| 7 | 100797100 | AP1S1 | 0.0049 | REG | −0.46 | <0.01 | −0.91 | <0.01 |
| 7 | 100798274 | AP1S1 | 0.3632 | REG | 0.07 | <0.01 | 0.14 | <0.01 |
| 7 | 129252980 | NRF1 | 0.0098 | REG | −3.02 | 0.10 | −5.79 | 0.17 |
| 7 | 129253370 | NRF1 | 0.4853 | REG | 1.19 | 0.83 | 2.28 | 1.46 |
| 7 | 129260534 | NRF1 | 0.4665 | REG | −1.36 | 1.08 | −2.61 | 1.91 |
| 7 | 129265143 | NRF1 | 0.1648 | REG | −0.76 | 0.18 | −1.46 | 0.32 |
| 7 | 129267301 | NRF1 | 0.0049 | REG | −1.57 | 0.03 | −3.01 | 0.05 |
| 7 | 129283698 | NRF1 | 0.1530 | REG | 0.55 | 0.09 | 1.06 | 0.16 |
| 7 | 129287542 | NRF1 | 0.0083 | REG | 0.90 | 0.02 | 1.72 | 0.03 |
| 7 | 129288493 | NRF1 | 0.0148 | REG | −0.87 | 0.02 | −1.67 | 0.04 |
| 7 | 129294299 | NRF1 | 0.0198 | REG | −0.71 | 0.02 | −1.36 | 0.03 |
| 7 | 129300274 | NRF1 | 0.0049 | REG | 1.55 | 0.03 | 2.97 | 0.05 |
| 7 | 129303786 | NRF1 | 0.1320 | REG | 0.55 | 0.07 | 1.05 | 0.13 |
| 7 | 129304415 | NRF1 | 0.0163 | REG | −1.58 | 0.08 | −3.03 | 0.15 |
| 7 | 129314947 | NRF1 | 0.0065 | REG | −1.87 | 0.03 | −3.58 | 0.06 |
| 7 | 129392863 | NRF1 | 0.0033 | REG | 2.65 | 0.06 | 5.09 | 0.10 |
| 7 | 134007960 | AKR1B1 | 0.0033 | REG | 0.00 | 0.00 | −0.35 | <0.01 |
| 7 | 134124005 | AKR1B1 | 0.1526 | REG | 0.00 | 0.00 | −0.15 | <0.01 |
| 7 | 134135621 | AKR1B1 | 0.0049 | N-S | 0.00 | 0.00 | 0.01 | <0.01 |
| 7 | 134143814 | AKR1B1 | 0.0016 | N-S | 0.00 | 0.00 | 0.72 | 0.01 |
| 7 | 134147698 | AKR1B1 | 0.0016 | REG | 0.00 | 0.00 | 0.36 | <0.01 |
| 7 | 134211221 | AKR1B1 | 0.4824 | REG | 0.00 | 0.00 | 0.10 | <0.01 |
| 7 | 134211231 | AKR1B1 | 0.0147 | REG | 0.00 | 0.00 | 0.30 | <0.01 |
| 7 | 134944374 | CNOT4 | 0.0081 | REG | 0.38 | <0.01 | 0.35 | <0.01 |
| 7 | 134944544 | CNOT4 | 0.4424 | REG | 0.04 | <0.01 | 0.04 | <0.01 |
| 7 | 135050891 | CNOT4 | 0.0033 | REG | 0.34 | <0.01 | 0.32 | <0.01 |
| 7 | 135052200 | CNOT4 | 0.0382 | REG | 0.15 | <0.01 | 0.14 | <0.01 |
| 7 | 135073576 | CNOT4 | 0.0066 | REG | 0.23 | <0.01 | 0.21 | <0.01 |
| 7 | 135082953 | CNOT4 | 0.0661 | N-S | 0.00 | 0.00 | 0.00 | 0.00 |
| 7 | 135123060 | CNOT4 | 0.0049 | N-S | 0.00 | 0.00 | 0.00 | 0.00 |
| 7 | 135150784 | CNOT4 | 0.0033 | REG | 0.61 | 0.01 | 0.56 | <0.01 |
| 7 | 135240927 | CNOT4 | 0.0033 | REG | −0.48 | <0.01 | −0.44 | <0.01 |
| 7 | 140148684 | MKRN1 | 0.0870 | REG | −0.20 | 0.01 | −0.28 | 0.01 |
| 7 | 140155296 | MKRN1 | 0.1021 | REG | 0.28 | 0.02 | 0.38 | 0.01 |
| 7 | 140157936 | MKRN1 | 0.0049 | REG | −0.34 | <0.01 | −0.46 | <0.01 |
| 7 | 140158851 | MKRN1 | 0.1443 | N-S | −0.03 | <0.01 | −0.06 | <0.01 |
| 7 | 140179543 | MKRN1 | 0.0016 | REG | 0.44 | <0.01 | 0.61 | <0.01 |
| 7 | 140214502 | MKRN1 | 0.0017 | REG | −0.53 | <0.01 | −0.74 | <0.01 |
| 7 | 140214503 | MKRN1 | 0.0049 | REG | 0.37 | <0.01 | 0.51 | <0.01 |
| 7 | 142640113 | KEL | 0.0033 | N-S | 0.00 | 0.00 | 0.00 | 0.00 |
| 7 | 142640661 | KEL | 0.0082 | N-S | 0.01 | <0.01 | 0.02 | <0.01 |
| 7 | 142641725 | KEL | 0.0016 | REG | −0.58 | <0.01 | −0.70 | <0.01 |
| 7 | 142651354 | KEL | 0.0083 | N-S | 0.59 | 0.01 | 0.98 | 0.01 |
| 7 | 142655008 | KEL | 0.0149 | N-S | 0.00 | 0.00 | 0.00 | 0.00 |
| 7 | 142655047 | KEL | 0.0016 | N-S | 0.00 | 0.00 | 0.00 | 0.00 |
| 7 | 142718726 | KEL | 0.0131 | REG | 0.71 | 0.01 | 0.85 | 0.01 |
| 7 | 150027284 | C7orf29 | 0.0941 | REG | −0.09 | <0.01 | 0.00 | 0.00 |
| 7 | 150028098 | C7orf29 | 0.0593 | N-S | 0.20 | 0.01 | 0.00 | 0.00 |
| 7 | 150041836 | REPIN1 | 0.1032 | REG | −0.54 | 0.06 | 0.00 | 0.00 |
| 7 | 150043205 | REPIN1 | 0.0978 | REG | 0.68 | 0.10 | 0.00 | 0.00 |
| 7 | 150067640 | REPIN1 | 0.2126 | REG | 0.24 | 0.03 | 0.00 | 0.00 |
| 7 | 150068371 | REPIN1 | 0.2084 | N-S | 0.06 | <0.01 | 0.00 | 0.00 |
| 7 | 150068605 | REPIN1 | 0.1577 | N-S | 0.07 | <0.01 | 0.00 | 0.00 |
| 7 | 150068620 | REPIN1 | 0.1577 | N-S | 0.09 | <0.01 | 0.00 | 0.00 |
| 9 | 5778403 | ERMP1 | 0.0306 | REG | −0.19 | <0.01 | 0.00 | 0.00 |
| 9 | 5787242 | ERMP1 | 0.0049 | N-S | 0.00 | 0.00 | 0.00 | 0.00 |
| 9 | 5809295 | ERMP1 | 0.0016 | REG | 0.39 | <0.01 | 0.00 | 0.00 |
| 9 | 5816898 | ERMP1 | 0.0414 | REG | 0.21 | 0.01 | 0.00 | 0.00 |
| 9 | 5819634 | ERMP1 | 0.0381 | REG | 0.16 | <0.01 | 0.00 | 0.00 |
| 9 | 5826333 | ERMP1 | 0.0049 | REG | −0.63 | <0.01 | 0.00 | 0.00 |
| 9 | 5832439 | ERMP1 | 0.0436 | REG | 0.20 | <0.01 | 0.00 | 0.00 |
| 9 | 5832719 | ERMP1 | 0.0116 | N-S | 0.00 | 0.00 | 0.00 | 0.00 |
| 9 | 5832782 | ERMP1 | 0.0166 | N-S | 0.00 | 0.00 | 0.00 | 0.00 |
| 9 | 5832840 | ERMP1 | 0.0084 | N-S | −0.08 | <0.01 | 0.00 | 0.00 |
| 9 | 5832849 | ERMP1 | 0.0033 | N-S | 0.00 | 0.00 | 0.00 | 0.00 |
| 9 | 5889099 | ERMP1 | 0.1703 | REG | 0.11 | <0.01 | 0.00 | 0.00 |
| 9 | 15423548 | SNAPC3 | 0.0796 | REG | −0.25 | 0.01 | −0.24 | <0.01 |
| 9 | 15424682 | SNAPC3 | 0.0033 | REG | −0.40 | <0.01 | −0.38 | <0.01 |
| 9 | 15424989 | SNAPC3 | 0.0065 | REG | 0.38 | <0.01 | 0.37 | <0.01 |
| 9 | 15425845 | SNAPC3 | 0.0065 | REG | −0.48 | <0.01 | −0.46 | <0.01 |
| 9 | 15435967 | SNAPC3 | 0.0588 | REG | 0.38 | 0.01 | 0.36 | 0.01 |
| 9 | 15447204 | SNAPC3 | 0.0016 | N-S | −0.48 | <0.01 | −0.63 | <0.01 |
| 9 | 15459821 | SNAPC3 | 0.0264 | N-S | −0.46 | 0.01 | −0.60 | 0.01 |
| 9 | 15462521 | SNAPC3 | 0.0285 | REG | 0.21 | <0.01 | 0.20 | <0.01 |
| 9 | 15462546 | SNAPC3 | 0.0181 | REG | −0.04 | <0.01 | −0.04 | <0.01 |
| 9 | 21387979 | IFNA8 | 0.0065 | REG | −1.25 | 0.03 | −1.11 | 0.01 |
| 9 | 21409584 | IFNA8 | 0.0131 | N-S | 0.00 | 0.00 | 0.00 | 0.00 |
| 9 | 21436231 | IFNA8 | 0.0049 | REG | −0.74 | 0.01 | −0.66 | <0.01 |
| 9 | 27542457 | C9orf72 | 0.0033 | REG | −0.40 | <0.01 | 0.00 | 0.00 |
| 9 | 27561628 | C9orf72 | 0.0554 | N-S | 0.35 | 0.01 | 0.00 | 0.00 |
| 9 | 27566141 | C9orf72 | 0.2721 | REG | 0.07 | <0.01 | 0.00 | 0.00 |
| 9 | 27576823 | C9orf72 | 0.0373 | REG | 0.12 | <0.01 | 0.00 | 0.00 |
| 9 | 27746174 | C9orf72 | 0.0049 | REG | 0.39 | <0.01 | 0.00 | 0.00 |
| 9 | 27750329 | C9orf72 | 0.0197 | REG | 0.25 | <0.01 | 0.00 | 0.00 |
| 9 | 27938013 | C9orf72 | 0.0033 | REG | −0.33 | <0.01 | 0.00 | 0.00 |
| 9 | 27947491 | C9orf72 | 0.0346 | REG | 0.14 | <0.01 | 0.00 | 0.00 |
| 9 | 32969997 | APTX | 0.0016 | REG | 0.36 | <0.01 | 0.32 | <0.01 |
| 9 | 32994500 | APTX | 0.4203 | REG | 0.36 | 0.07 | 0.33 | 0.03 |
| 9 | 33001449 | APTX | 0.4328 | REG | −0.23 | 0.03 | −0.20 | 0.01 |
| 9 | 34616979 | DCTN3 | 0.0050 | REG | 0.21 | <0.01 | 0.23 | <0.01 |
| 9 | 37875240 | MCART1 | 0.0016 | REG | −0.86 | 0.01 | −0.60 | <0.01 |
| 9 | 80888236 | PSAT1 | 0.0016 | REG | 0.00 | 0.00 | −0.39 | <0.01 |
| 9 | 80888623 | PSAT1 | 0.0197 | REG | 0.00 | 0.00 | 0.17 | <0.01 |
| 9 | 80889625 | PSAT1 | 0.0016 | REG | 0.00 | 0.00 | 0.71 | <0.01 |
| 9 | 80907874 | PSAT1 | 0.0082 | REG | 0.00 | 0.00 | −0.57 | <0.01 |
| 9 | 80946291 | PSAT1 | 0.4198 | REG | 0.00 | 0.00 | 0.06 | <0.01 |
| 9 | 100418331 | NCBP1 | 0.0083 | N-S | −0.08 | <0.01 | 0.00 | 0.00 |
| 9 | 100418820 | NCBP1 | 0.0082 | REG | −0.49 | <0.01 | 0.00 | 0.00 |
| 9 | 104168271 | ZNF189 | 0.0723 | REG | −0.12 | <0.01 | −0.10 | <0.01 |
| 9 | 104171069 | ZNF189 | 0.0033 | N-S | −0.41 | <0.01 | −0.46 | <0.01 |
| 9 | 104171608 | ZNF189 | 0.0033 | N-S | −0.13 | <0.01 | −0.15 | <0.01 |
| 9 | 104171770 | ZNF189 | 0.0034 | N-S | 0.00 | 0.00 | 0.00 | 0.00 |
| 9 | 110084328 | RAD23B | 0.2955 | N-S | 0.00 | 0.00 | 0.00 | 0.00 |
| 9 | 110098733 | KLF4 | 0.0033 | REG | −0.70 | 0.01 | −0.37 | <0.01 |
| 9 | 110249165 | KLF4 | 0.0049 | REG | 0.87 | <0.01 | 0.46 | <0.01 |
| 9 | 110249816 | KLF4 | 0.0033 | N-S | 0.41 | <0.01 | 0.30 | <0.01 |
| 9 | 110685132 | KLF4 | 0.0066 | REG | −0.50 | 0.01 | −0.26 | <0.01 |
| 9 | 112811038 | AKAP2 | 0.0635 | N-S | −0.11 | <0.01 | −0.11 | <0.01 |
| 9 | 112898576 | AKAP2 | 0.0065 | N-S | −0.42 | <0.01 | −0.41 | <0.01 |
| 9 | 112898708 | AKAP2 | 0.0050 | N-S | −0.64 | 0.01 | −0.63 | <0.01 |
| 9 | 112898843 | AKAP2 | 0.0050 | N-S | −0.16 | <0.01 | −0.16 | <0.01 |
| 9 | 112899430 | AKAP2 | 0.0017 | N-S | −0.12 | <0.01 | −0.12 | <0.01 |
| 9 | 112899715 | AKAP2 | 0.0016 | N-S | 0.00 | 0.00 | 0.00 | 0.00 |
| 9 | 112900199 | AKAP2 | 0.3958 | N-S | 0.00 | 0.00 | 0.00 | 0.00 |
| 9 | 112900466 | AKAP2 | 0.0100 | N-S | 0.00 | 0.00 | 0.00 | 0.00 |
| 9 | 112918755 | AKAP2 | 0.0065 | N-S | −0.67 | 0.01 | −0.66 | <0.01 |
| 9 | 115803080 | ZFP37 | 0.0049 | REG | 0.44 | <0.01 | 0.00 | 0.00 |
| 9 | 115805036 | ZFP37 | 0.0746 | N-S | −0.52 | 0.05 | 0.00 | 0.00 |
| 9 | 115806375 | ZFP37 | 0.0033 | N-S | 0.00 | 0.00 | 0.00 | 0.00 |
| 9 | 115806434 | ZFP37 | 0.0049 | N-S | 0.00 | 0.00 | 0.00 | 0.00 |
| 9 | 115817156 | ZFP37 | 0.0800 | REG | −0.15 | <0.01 | 0.00 | 0.00 |
| 9 | 115818949 | ZFP37 | 0.4248 | N-S | 0.00 | 0.00 | 0.00 | 0.00 |
| 9 | 115819250 | ZFP37 | 0.1352 | REG | 0.13 | 0.01 | 0.00 | 0.00 |
| 9 | 115821317 | ZFP37 | 0.1521 | REG | −1.27 | 0.49 | 0.00 | 0.00 |
| 9 | 115821395 | ZFP37 | 0.1507 | REG | 1.12 | 0.38 | 0.00 | 0.00 |
| 9 | 116042291 | PRPF4 | 0.0016 | REG | 0.54 | <0.01 | 0.46 | <0.01 |
| 9 | 116047937 | PRPF4 | 0.0049 | REG | −0.84 | 0.01 | −0.72 | <0.01 |
| 9 | 116052105 | PRPF4 | 0.0115 | REG | 1.23 | 0.02 | 1.06 | 0.01 |
| 9 | 116053878 | PRPF4 | 0.0016 | N-S | 0.30 | <0.01 | 0.36 | <0.01 |
| 9 | 116055502 | PRPF4 | 0.0082 | REG | −0.33 | <0.01 | −0.28 | <0.01 |
| 9 | 123575167 | PSMD5 | 0.0050 | REG | 0.93 | 0.02 | 0.97 | 0.01 |
| 9 | 123578733 | PSMD5 | 0.0147 | REG | 0.73 | 0.01 | 0.77 | 0.01 |
| 9 | 123586801 | PSMD5 | 0.0082 | N-S | −0.19 | <0.01 | −0.27 | <0.01 |
| 9 | 123591028 | PSMD5 | 0.0065 | REG | −0.52 | 0.01 | −0.55 | <0.01 |
| 9 | 123592637 | PSMD5 | 0.0050 | REG | 0.76 | 0.02 | 0.80 | 0.01 |
| 9 | 123605126 | PSMD5 | 0.2195 | N-S | −0.55 | 0.13 | −0.80 | 0.13 |
| 9 | 123969834 | GSN | 0.2442 | REG | 0.00 | 0.00 | −0.11 | <0.01 |
| 9 | 123983945 | GSN | 0.0680 | REG | 0.00 | 0.00 | 0.13 | <0.01 |
| 9 | 123988378 | GSN | 0.0065 | REG | 0.00 | 0.00 | 0.29 | <0.01 |
| 9 | 123994676 | GSN | 0.4787 | REG | 0.00 | 0.00 | −0.59 | 0.09 |
| 9 | 124018929 | GSN | 0.4762 | REG | 0.00 | 0.00 | 0.32 | 0.03 |
| 9 | 124048621 | GSN | 0.0067 | REG | 0.00 | 0.00 | 0.23 | <0.01 |
| 9 | 124064278 | GSN | 0.0082 | N-S | 0.00 | 0.00 | 0.00 | 0.00 |
| 9 | 124065224 | GSN | 0.2041 | N-S | 0.00 | 0.00 | −0.65 | 0.08 |
| 9 | 124072597 | GSN | 0.2998 | REG | 0.00 | 0.00 | 0.07 | <0.01 |
| 9 | 124075254 | GSN | 0.4462 | REG | 0.00 | 0.00 | −0.06 | <0.01 |
| 9 | 124083642 | GSN | 0.0049 | N-S | 0.00 | 0.00 | −0.02 | <0.01 |
| 9 | 124088908 | GSN | 0.0196 | N-S | 0.00 | 0.00 | 0.00 | 0.00 |
| 9 | 124089692 | GSN | 0.0248 | N-S | 0.00 | 0.00 | 0.00 | 0.00 |
| 9 | 124093997 | GSN | 0.0229 | REG | 0.00 | 0.00 | 0.20 | <0.01 |
| 9 | 127124263 | PSMB7 | 0.0679 | REG | 0.12 | <0.01 | 0.13 | <0.01 |
| 9 | 127136924 | PSMB7 | 0.0049 | REG | 0.58 | <0.01 | 0.66 | <0.01 |
| 9 | 127147404 | PSMB7 | 0.0033 | REG | 0.59 | <0.01 | 0.68 | <0.01 |
| 9 | 127147460 | PSMB7 | 0.0033 | REG | 0.92 | 0.01 | 1.05 | <0.01 |
| 9 | 127161254 | PSMB7 | 0.0035 | REG | 1.26 | 0.01 | 1.43 | 0.01 |
| 9 | 127177161 | PSMB7 | 0.3162 | N-S | 0.00 | 0.00 | 0.00 | 0.00 |
| 9 | 131880235 | PPP2R4 | 0.0163 | REG | −0.54 | 0.01 | −0.33 | <0.01 |
| 9 | 131891157 | PPP2R4 | 0.0016 | REG | 0.74 | <0.01 | 0.45 | <0.01 |
| 9 | 131897131 | PPP2R4 | 0.0198 | N-S | 0.00 | 0.00 | 0.00 | 0.00 |
| 9 | 131901396 | PPP2R4 | 0.0049 | REG | −1.28 | 0.01 | −0.77 | <0.01 |
| 9 | 131909736 | PPP2R4 | 0.0451 | N-S | 0.81 | 0.07 | 0.67 | 0.02 |
| 9 | 131912446 | PPP2R4 | 0.0150 | REG | 0.36 | 0.01 | 0.22 | <0.01 |
| 9 | 136223833 | SURF2 | 0.0065 | N-S | 0.00 | 0.00 | −0.08 | <0.01 |
| 9 | 136225209 | SURF2 | 0.1214 | REG | 0.00 | 0.00 | 0.09 | <0.01 |
| 9 | 136227213 | SURF2 | 0.0082 | N-S | 0.00 | 0.00 | 0.00 | 0.00 |
| 9 | 136227260 | SURF2 | 0.3768 | N-S | 0.00 | 0.00 | 0.00 | 0.00 |
| 9 | 136228007 | SURF2 | 0.0231 | N-S | 0.00 | 0.00 | −0.02 | <0.01 |
| 9 | 139538247 | EGFL7 | 0.0250 | REG | 0.00 | 0.00 | −0.30 | <0.01 |
| 9 | 139547693 | EGFL7 | 0.0016 | REG | 0.00 | 0.00 | −0.37 | <0.01 |
| 9 | 139547999 | EGFL7 | 0.1441 | REG | 0.00 | 0.00 | 0.11 | <0.01 |
| 9 | 139564387 | EGFL7 | 0.0033 | N-S | 0.00 | 0.00 | 0.00 | 0.00 |
| 9 | 139564668 | EGFL7 | 0.2536 | N-S | 0.00 | 0.00 | 0.00 | 0.00 |
| 9 | 139566450 | EGFL7 | 0.0016 | N-S | 0.00 | 0.00 | 0.00 | 0.00 |
| 9 | 140093585 | TPRN | 0.0016 | N-S | 0.37 | <0.01 | 0.00 | 0.00 |
| 9 | 140094011 | TPRN | 0.0016 | N-S | 0.42 | <0.01 | 0.00 | 0.00 |
| 9 | 140094091 | TPRN | 0.0049 | N-S | 0.01 | <0.01 | 0.00 | 0.00 |
| 9 | 140096931 | TPRN | 0.0033 | REG | 0.51 | <0.01 | 0.00 | 0.00 |
| 11 | 237087 | PSMD13 | 0.1911 | N-S | 0.00 | 0.00 | 0.00 | 0.00 |
| 11 | 237875 | PSMD13 | 0.1733 | REG | −0.16 | 0.01 | 0.00 | 0.00 |
| 11 | 239535 | PSMD13 | 0.0016 | REG | 0.29 | <0.01 | 0.00 | 0.00 |
| 11 | 247377 | PSMD13 | 0.0065 | N-S | 0.00 | 0.00 | 0.00 | 0.00 |
| 11 | 247630 | PSMD13 | 0.2896 | REG | 0.08 | <0.01 | 0.00 | 0.00 |
| 11 | 252941 | PSMD13 | 0.1491 | REG | 0.12 | <0.01 | 0.00 | 0.00 |
| 11 | 314207 | IFITM1 | 0.0244 | N-S | 0.00 | 0.00 | 0.00 | 0.00 |
| 11 | 316299 | IFITM3 | 0.2366 | REG | 0.13 | 0.01 | 0.11 | <0.01 |
| 11 | 317089 | IFITM3 | 0.0016 | REG | 0.56 | <0.01 | 0.47 | <0.01 |
| 11 | 320805 | IFITM3 | 0.0314 | N-S | −0.24 | <0.01 | −0.27 | <0.01 |
| 11 | 626220 | SCT | 0.0912 | REG | 0.16 | <0.01 | 0.15 | <0.01 |
| 11 | 636079 | SCT | 0.0049 | REG | −0.93 | 0.01 | −0.86 | <0.01 |
| 11 | 840363 | POLR2L | 0.3753 | REG | 0.11 | 0.01 | 0.00 | 0.00 |
| 11 | 840477 | POLR2L | 0.2210 | REG | 0.12 | 0.01 | 0.00 | 0.00 |
| 11 | 2924591 | SLC22A18 | 0.1043 | N-S | 0.00 | 0.00 | 0.12 | <0.01 |
| 11 | 2924610 | SLC22A18 | 0.3566 | N-S | 0.00 | 0.00 | 0.00 | 0.00 |
| 11 | 2930440 | SLC22A18 | 0.0455 | N-S | 0.00 | 0.00 | 0.00 | 0.00 |
| 11 | 2943342 | SLC22A18 | 0.0033 | N-S | 0.00 | 0.00 | 0.66 | <0.01 |
| 11 | 2943671 | SLC22A18 | 0.1441 | N-S | 0.00 | 0.00 | 0.00 | 0.00 |
| 11 | 2947861 | SLC22A18 | 0.0033 | REG | 0.00 | 0.00 | 0.31 | <0.01 |
| 11 | 2961512 | PHLDA2 | 0.2676 | REG | 0.08 | <0.01 | 0.00 | 0.00 |
| 11 | 6681654 | MRPL17 | 0.2333 | REG | −0.10 | <0.01 | −0.06 | <0.01 |
| 11 | 47456867 | PSMC3 | 0.4235 | REG | 0.07 | <0.01 | 0.00 | 0.00 |
| 11 | 47602729 | NDUFS3 | 0.3591 | REG | −0.10 | 0.01 | 0.00 | 0.00 |
| 11 | 47605950 | NDUFS3 | 0.0016 | N-S | −0.72 | <0.01 | 0.00 | 0.00 |
| 11 | 62538661 | TAF6L | 0.0082 | REG | 0.00 | 0.00 | 0.32 | <0.01 |
| 11 | 62542813 | TAF6L | 0.0049 | REG | 0.00 | 0.00 | −0.38 | <0.01 |
| 11 | 62542850 | TAF6L | 0.0033 | REG | 0.00 | 0.00 | 0.44 | <0.01 |
| 11 | 62551017 | TAF6L | 0.0033 | REG | 0.00 | 0.00 | 0.35 | <0.01 |
| 11 | 64085785 | PRDX5 | 0.0238 | N-S | 0.00 | 0.00 | −0.03 | <0.01 |
| 11 | 64085938 | PRDX5 | 0.0050 | REG | 0.00 | 0.00 | −0.60 | <0.01 |
| 11 | 64088252 | PRDX5 | 0.0016 | N-S | 0.00 | 0.00 | 0.00 | 0.00 |
| 11 | 64105454 | PRDX5 | 0.1275 | REG | 0.00 | 0.00 | 0.21 | 0.01 |
| 11 | 64106320 | PRDX5 | 0.0033 | REG | 0.00 | 0.00 | −0.41 | <0.01 |
| 11 | 65670255 | FOSL1 | 0.0165 | REG | −0.45 | <0.01 | −0.47 | <0.01 |
| 11 | 66615020 | PC | 0.0230 | REG | −0.20 | <0.01 | −0.41 | <0.01 |
| 11 | 66633768 | PC | 0.0016 | N-S | 0.04 | <0.01 | 0.11 | <0.01 |
| 11 | 66650060 | PC | 0.1871 | REG | −0.05 | <0.01 | −0.11 | <0.01 |
| 11 | 66694417 | PC | 0.0017 | REG | 0.43 | <0.01 | 0.89 | <0.01 |
| 11 | 66711718 | PC | 0.0065 | REG | −0.49 | <0.01 | −1.01 | <0.01 |
| 11 | 66728547 | PC | 0.0049 | REG | 0.28 | <0.01 | 0.58 | <0.01 |
| 11 | 66820801 | RHOD | 0.0359 | REG | 0.36 | 0.01 | 0.00 | 0.00 |
| 11 | 66831749 | RHOD | 0.0147 | REG | 0.55 | 0.01 | 0.00 | 0.00 |
| 11 | 66832850 | RHOD | 0.0033 | REG | 0.47 | <0.01 | 0.00 | 0.00 |
| 11 | 66834232 | RHOD | 0.0083 | N-S | −0.95 | 0.03 | 0.00 | 0.00 |
| 11 | 66837019 | RHOD | 0.0283 | REG | −0.23 | <0.01 | 0.00 | 0.00 |
| 11 | 66837996 | RHOD | 0.0065 | N-S | −0.84 | 0.01 | 0.00 | 0.00 |
| 11 | 67288594 | CABP2 | 0.3911 | N-S | 0.62 | 0.21 | 0.80 | 0.17 |
| 11 | 67809268 | TCIRG1 | 0.0369 | N-S | 1.07 | 0.08 | 1.38 | 0.06 |
| 11 | 67810474 | TCIRG1 | 0.0065 | N-S | 0.01 | <0.01 | 0.01 | <0.01 |
| 11 | 67814983 | TCIRG1 | 0.0033 | N-S | 0.83 | 0.01 | 1.07 | 0.01 |
| 11 | 67816463 | TCIRG1 | 0.2509 | N-S | 0.00 | 0.00 | 0.00 | 0.00 |
| 11 | 67818269 | TCIRG1 | 0.0065 | N-S | 0.00 | 0.00 | 0.00 | 0.00 |
| 11 | 70037637 | FADD | 0.0165 | REG | −0.29 | <0.01 | −0.21 | <0.01 |
| 11 | 70038218 | FADD | 0.3792 | REG | 0.07 | <0.01 | 0.05 | <0.01 |
| 11 | 70053547 | FADD | 0.3459 | REG | −0.06 | <0.01 | −0.04 | <0.01 |
| 11 | 70054075 | FADD | 0.0098 | REG | 0.56 | <0.01 | 0.40 | <0.01 |
| 11 | 70056704 | FADD | 0.0281 | REG | −0.25 | <0.01 | −0.18 | <0.01 |
| 11 | 70111948 | FADD | 0.0033 | REG | 1.61 | 0.01 | 1.15 | <0.01 |
| 11 | 71640070 | RNF121 | 0.0297 | REG | 0.23 | <0.01 | 0.25 | <0.01 |
| 11 | 71647464 | RNF121 | 0.0034 | REG | −0.57 | <0.01 | −0.61 | <0.01 |
| 11 | 71650582 | RNF121 | 0.0066 | REG | −0.52 | <0.01 | −0.56 | <0.01 |
| 11 | 71687113 | RNF121 | 0.0016 | REG | −0.42 | <0.01 | −0.45 | <0.01 |
| 11 | 71687488 | RNF121 | 0.0049 | REG | 0.48 | <0.01 | 0.52 | <0.01 |
| 11 | 72931983 | P2RY2 | 0.0532 | REG | −0.11 | <0.01 | −0.10 | <0.01 |
| 11 | 72933225 | P2RY2 | 0.0049 | REG | 0.36 | <0.01 | 0.34 | <0.01 |
| 11 | 72945341 | P2RY2 | 0.0364 | N-S | −0.01 | <0.01 | −0.01 | <0.01 |
| 11 | 72946140 | P2RY2 | 0.4395 | N-S | −0.01 | <0.01 | −0.01 | <0.01 |
| 11 | 72946202 | P2RY2 | 0.0016 | N-S | 0.00 | 0.00 | 0.00 | 0.00 |
| 11 | 72946204 | P2RY2 | 0.0985 | N-S | −0.45 | 0.04 | −0.59 | 0.03 |
| 11 | 72946279 | P2RY2 | 0.0164 | N-S | 0.00 | 0.00 | 0.00 | 0.00 |
| 11 | 72946308 | P2RY2 | 0.0131 | REG | −0.22 | <0.01 | −0.21 | <0.01 |
| 11 | 72972860 | P2RY2 | 0.0888 | REG | −0.11 | <0.01 | −0.10 | <0.01 |
| 11 | 72973042 | P2RY2 | 0.0507 | REG | 0.11 | <0.01 | 0.11 | <0.01 |
| 11 | 75238118 | SERPINH1 | 0.0800 | REG | −0.43 | 0.03 | −0.38 | 0.01 |
| 11 | 75239722 | SERPINH1 | 0.0799 | REG | 0.52 | 0.05 | 0.46 | 0.02 |
| 11 | 75274659 | SERPINH1 | 0.0147 | REG | 0.25 | <0.01 | 0.22 | <0.01 |
| 11 | 75286210 | SERPINH1 | 0.0098 | REG | 0.36 | <0.01 | 0.32 | <0.01 |
| 11 | 75287177 | SERPINH1 | 0.0033 | REG | 0.31 | <0.01 | 0.28 | <0.01 |
| 11 | 75293370 | SERPINH1 | 0.0049 | REG | 0.50 | <0.01 | 0.45 | <0.01 |
| 11 | 77935643 | GAB2 | 0.0154 | REG | 0.00 | 0.00 | −1.15 | 0.03 |
| 11 | 77937768 | GAB2 | 0.0082 | N-S | 0.00 | 0.00 | 3.09 | 0.08 |
| 11 | 77960699 | GAB2 | 0.0016 | REG | 0.00 | 0.00 | −1.55 | 0.02 |
| 11 | 77961419 | GAB2 | 0.0815 | N-S | 0.00 | 0.00 | 0.00 | 0.00 |
| 11 | 78081926 | GAB2 | 0.0033 | REG | 0.00 | 0.00 | 1.56 | 0.01 |
| 11 | 93852880 | PANX1 | 0.1330 | REG | 0.64 | 0.09 | 0.48 | 0.03 |
| 11 | 93858774 | PANX1 | 0.0102 | REG | −0.41 | 0.01 | −0.31 | <0.01 |
| 11 | 93862493 | PANX1 | 0.1736 | N-S | 0.00 | 0.00 | 0.00 | 0.00 |
| 11 | 93864393 | PANX1 | 0.1380 | REG | −0.53 | 0.06 | −0.40 | 0.02 |
| 11 | 93895859 | PANX1 | 0.0016 | REG | −0.73 | <0.01 | −0.55 | <0.01 |
| 11 | 93911659 | PANX1 | 0.0033 | N-S | 0.00 | 0.00 | 0.00 | 0.00 |
| 11 | 93913036 | PANX1 | 0.0679 | N-S | 0.00 | 0.00 | 0.00 | 0.00 |
| 11 | 93913392 | PANX1 | 0.0147 | N-S | 0.00 | 0.00 | 0.00 | 0.00 |
| 11 | 93915248 | PANX1 | 0.3197 | REG | −0.14 | 0.01 | −0.10 | <0.01 |
| 11 | 94036653 | PANX1 | 0.0131 | REG | 0.42 | <0.01 | 0.32 | <0.01 |
| 11 | 102180723 | BIRC3 | 0.0049 | REG | −0.84 | <0.01 | −0.49 | <0.01 |
| 11 | 102184196 | BIRC3 | 0.0082 | REG | 0.48 | <0.01 | 0.28 | <0.01 |
| 11 | 102187213 | BIRC3 | 0.0281 | REG | −0.25 | 0.01 | −0.15 | <0.01 |
| 11 | 102196019 | BIRC3 | 0.1103 | N-S | 0.00 | 0.00 | 0.00 | 0.00 |
| 11 | 104841352 | CASP5 | 0.0098 | REG | 0.60 | <0.01 | 0.58 | <0.01 |
| 11 | 104869651 | CASP5 | 0.0049 | N-S | 0.00 | 0.00 | 0.00 | 0.00 |
| 11 | 104869708 | CASP5 | 0.3658 | N-S | 0.00 | 0.00 | 0.00 | 0.00 |
| 11 | 104871047 | CASP5 | 0.0016 | N-S | 0.00 | 0.00 | 0.00 | 0.00 |
| 11 | 104877927 | CASP5 | 0.4908 | N-S | 0.00 | 0.00 | 0.00 | 0.00 |
| 11 | 104879628 | CASP5 | 0.0166 | N-S | 0.00 | 0.00 | 0.00 | 0.00 |
| 11 | 104879658 | CASP5 | 0.0131 | N-S | −0.01 | <0.01 | −0.01 | <0.01 |
| 11 | 104895003 | CASP5 | 0.0114 | REG | −0.34 | <0.01 | −0.33 | <0.01 |
| 11 | 113608355 | ZW10 | 0.0033 | N-S | 0.00 | 0.00 | 0.00 | 0.00 |
| 11 | 113609970 | ZW10 | 0.0050 | N-S | −0.12 | <0.01 | 0.00 | 0.00 |
| 11 | 113621175 | ZW10 | 0.0016 | REG | −0.34 | <0.01 | 0.00 | 0.00 |
| 11 | 113626329 | ZW10 | 0.0937 | REG | 0.05 | <0.01 | 0.00 | 0.00 |
| 11 | 113629002 | ZW10 | 0.0016 | REG | 0.35 | <0.01 | 0.00 | 0.00 |
| 11 | 113642641 | ZW10 | 0.0033 | REG | 0.25 | <0.01 | 0.00 | 0.00 |
| 11 | 117977723 | TMPRSS4 | 0.1769 | REG | −0.08 | <0.01 | −0.12 | <0.01 |
| 11 | 117978578 | TMPRSS4 | 0.0196 | N-S | 0.00 | 0.00 | 0.00 | 0.00 |
| 11 | 117982464 | TMPRSS4 | 0.0553 | N-S | 0.00 | 0.00 | 0.00 | 0.00 |
| 11 | 117982495 | TMPRSS4 | 0.0257 | N-S | 0.00 | 0.00 | 0.00 | 0.00 |
| 11 | 117983899 | TMPRSS4 | 0.0232 | REG | −0.18 | <0.01 | −0.27 | <0.01 |
| 11 | 117988105 | TMPRSS4 | 0.0992 | N-S | 0.51 | 0.05 | 1.05 | 0.10 |
| 11 | 118000289 | SCN4B | 0.0377 | REG | 0.00 | 0.00 | 0.19 | <0.01 |
| 11 | 119986109 | TRIM29 | 0.0033 | REG | 0.45 | <0.01 | 0.00 | 0.00 |
| 11 | 119986119 | TRIM29 | 0.0516 | N-S | 0.57 | 0.03 | 0.00 | 0.00 |
| 11 | 119990877 | TRIM29 | 0.0033 | REG | 0.35 | <0.01 | 0.00 | 0.00 |
| 11 | 119996653 | TRIM29 | 0.0423 | REG | 0.17 | <0.01 | 0.00 | 0.00 |
| 11 | 120008468 | TRIM29 | 0.0133 | N-S | 0.59 | 0.01 | 0.00 | 0.00 |
| 11 | 120078028 | TRIM29 | 0.4891 | REG | −0.08 | <0.01 | 0.00 | 0.00 |
| 11 | 126167696 | DCPS | 0.1609 | REG | 0.00 | 0.00 | −0.17 | <0.01 |
| 11 | 126169009 | DCPS | 0.1814 | REG | 0.00 | 0.00 | 0.28 | 0.01 |
| 11 | 126170990 | DCPS | 0.0065 | REG | 0.00 | 0.00 | −0.49 | <0.01 |
| 11 | 126171932 | DCPS | 0.0352 | REG | 0.00 | 0.00 | −0.21 | <0.01 |
| 11 | 126176484 | DCPS | 0.0016 | N-S | 0.00 | 0.00 | 0.00 | 0.00 |
| 11 | 126210792 | DCPS | 0.0065 | REG | 0.00 | 0.00 | −0.32 | <0.01 |
| 11 | 134255189 | B3GAT1 | 0.1443 | REG | 0.05 | <0.01 | 0.06 | <0.01 |
| 11 | 134264957 | B3GAT1 | 0.0065 | REG | 0.26 | <0.01 | 0.32 | <0.01 |
| 11 | 134272378 | B3GAT1 | 0.0049 | REG | 0.34 | <0.01 | 0.41 | <0.01 |
| 11 | 134290032 | B3GAT1 | 0.2689 | REG | 0.07 | <0.01 | 0.08 | <0.01 |
| 11 | 134300361 | B3GAT1 | 0.0049 | REG | 0.45 | <0.01 | 0.54 | <0.01 |
| 11 | 134301693 | B3GAT1 | 0.0488 | REG | 0.15 | <0.01 | 0.18 | <0.01 |
| 13 | 28009920 | MTIF3 | 0.3708 | N-S | 0.00 | 0.00 | 0.00 | 0.00 |
| 13 | 28010190 | MTIF3 | 0.2978 | REG | −0.33 | 0.06 | 0.00 | 0.00 |
| 13 | 28014173 | MTIF3 | 0.0049 | N-S | −0.19 | <0.01 | 0.00 | 0.00 |
| 13 | 28016521 | MTIF3 | 0.2887 | REG | 0.05 | <0.01 | 0.00 | 0.00 |
| 13 | 28018299 | MTIF3 | 0.2950 | REG | 0.26 | 0.03 | 0.00 | 0.00 |
| 13 | 28118710 | MTIF3 | 0.0049 | REG | 0.48 | <0.01 | 0.00 | 0.00 |
| 13 | 28567172 | FLT3 | 0.3574 | REG | −0.20 | 0.02 | −0.22 | 0.01 |
| 13 | 28587052 | FLT3 | 0.0016 | REG | 1.80 | 0.02 | 2.00 | 0.01 |
| 13 | 28594844 | FLT3 | 0.0164 | REG | 0.68 | 0.01 | 0.76 | 0.01 |
| 13 | 28595634 | FLT3 | 0.0049 | REG | 1.30 | 0.02 | 1.44 | 0.01 |
| 13 | 28601297 | FLT3 | 0.0016 | N-S | 3.24 | 0.11 | 4.21 | 0.09 |
| 13 | 28608473 | FLT3 | 0.0131 | N-S | 0.00 | 0.00 | 0.00 | 0.00 |
| 13 | 28623587 | FLT3 | 0.0099 | N-S | 0.00 | 0.00 | 0.00 | 0.00 |
| 13 | 28624294 | FLT3 | 0.4167 | N-S | 1.38 | 1.01 | 1.79 | 0.81 |
| 13 | 28665187 | FLT3 | 0.2061 | REG | 0.24 | 0.02 | 0.26 | 0.01 |
| 13 | 28670595 | FLT3 | 0.0448 | REG | 0.40 | 0.01 | 0.45 | 0.01 |
| 13 | 46703315 | LCP1 | 0.0035 | REG | 0.32 | <0.01 | 0.26 | <0.01 |
| 13 | 46705070 | LCP1 | 0.0049 | N-S | 0.00 | 0.00 | 0.00 | 0.00 |
| 13 | 46705628 | LCP1 | 0.0033 | REG | −0.67 | <0.01 | −0.54 | <0.01 |
| 13 | 46708291 | LCP1 | 0.0546 | N-S | −0.06 | <0.01 | −0.06 | <0.01 |
| 13 | 46752813 | LCP1 | 0.4181 | REG | 0.06 | <0.01 | 0.05 | <0.01 |
| 13 | 46791850 | LCP1 | 0.0049 | REG | −0.45 | <0.01 | −0.36 | <0.01 |
| 13 | 46800397 | LCP1 | 0.0065 | REG | 0.69 | 0.01 | 0.56 | <0.01 |
| 13 | 46806408 | LCP1 | 0.0016 | REG | 0.43 | <0.01 | 0.34 | <0.01 |
| 13 | 46832755 | LCP1 | 0.3000 | REG | −0.09 | <0.01 | −0.07 | <0.01 |
| 13 | 46858684 | LCP1 | 0.0049 | REG | 0.36 | <0.01 | 0.29 | <0.01 |
| 13 | 46912261 | LCP1 | 0.0049 | REG | −0.34 | <0.01 | −0.27 | <0.01 |
| 13 | 46913963 | LCP1 | 0.4352 | REG | −0.06 | <0.01 | −0.05 | <0.01 |
| 13 | 50799685 | DLEU7 | 0.0049 | REG | 0.45 | <0.01 | 0.45 | <0.01 |
| 13 | 50853031 | DLEU7 | 0.0033 | REG | 0.40 | <0.01 | 0.40 | <0.01 |
| 13 | 50874010 | DLEU7 | 0.0637 | REG | −0.11 | <0.01 | −0.11 | <0.01 |
| 13 | 51036651 | DLEU7 | 0.0016 | REG | 0.39 | <0.01 | 0.39 | <0.01 |
| 13 | 51122436 | DLEU7 | 0.0081 | REG | 0.43 | <0.01 | 0.43 | <0.01 |
| 13 | 51337912 | DLEU7 | 0.0148 | REG | −0.20 | <0.01 | −0.20 | <0.01 |
| 13 | 51349225 | DLEU7 | 0.0268 | REG | −0.19 | <0.01 | −0.20 | <0.01 |
| 13 | 51362573 | DLEU7 | 0.0049 | REG | 0.41 | <0.01 | 0.42 | <0.01 |
| 13 | 51363303 | DLEU7 | 0.2098 | REG | −0.07 | <0.01 | −0.07 | <0.01 |
| 13 | 76445432 | KCTD12 | 0.0402 | REG | 0.19 | <0.01 | 0.00 | 0.00 |
| 13 | 76455511 | KCTD12 | 0.0081 | REG | −0.60 | <0.01 | 0.00 | 0.00 |
| 13 | 77450255 | KCTD12 | 0.0065 | REG | 0.35 | <0.01 | 0.00 | 0.00 |
| 13 | 77451707 | KCTD12 | 0.0098 | REG | 0.62 | <0.01 | 0.00 | 0.00 |
| 13 | 111362093 | ING1 | 0.4297 | REG | 0.00 | 0.00 | 0.13 | <0.01 |
| 13 | 111367876 | ING1 | 0.0066 | N-S | 0.00 | 0.00 | 0.84 | 0.01 |
| 13 | 111368023 | ING1 | 0.0049 | N-S | 0.00 | 0.00 | 0.00 | 0.00 |
| 13 | 111368164 | ING1 | 0.0790 | N-S | 0.00 | 0.00 | 0.00 | 0.00 |
| 13 | 111369622 | ING1 | 0.0082 | REG | 0.00 | 0.00 | 0.77 | <0.01 |
| 13 | 111513946 | ING1 | 0.0647 | REG | 0.00 | 0.00 | 0.24 | 0.01 |
| 13 | 114244568 | TFDP1 | 0.0033 | REG | 0.36 | <0.01 | 0.00 | 0.00 |
| 13 | 114255369 | TFDP1 | 0.0016 | REG | 0.41 | <0.01 | 0.00 | 0.00 |
| 13 | 114267192 | TFDP1 | 0.0049 | REG | −0.67 | <0.01 | 0.00 | 0.00 |
| 13 | 114270408 | TFDP1 | 0.0049 | REG | 0.47 | <0.01 | 0.00 | 0.00 |
| 13 | 114290941 | TFDP1 | 0.0033 | N-S | 0.08 | <0.01 | 0.00 | 0.00 |
| 13 | 114292890 | TFDP1 | 0.0033 | REG | 0.33 | <0.01 | 0.00 | 0.00 |
| 13 | 114300547 | TFDP1 | 0.1057 | REG | 0.08 | <0.01 | 0.00 | 0.00 |
| 15 | 38247268 | SPRED1 | 0.0115 | REG | −0.21 | <0.01 | −0.27 | <0.01 |
| 15 | 38253005 | SPRED1 | 0.0065 | REG | −0.29 | <0.01 | −0.38 | <0.01 |
| 15 | 38327408 | SPRED1 | 0.1207 | REG | −0.03 | <0.01 | −0.04 | <0.01 |
| 15 | 38334210 | SPRED1 | 0.0499 | REG | −0.08 | <0.01 | −0.11 | <0.01 |
| 15 | 38335671 | SPRED1 | 0.0165 | REG | −0.13 | <0.01 | −0.17 | <0.01 |
| 15 | 38336589 | SPRED1 | 0.0297 | REG | −0.10 | <0.01 | −0.14 | <0.01 |
| 15 | 38448188 | SPRED1 | 0.0016 | REG | −0.31 | <0.01 | −0.40 | <0.01 |
| 15 | 38464874 | SPRED1 | 0.1291 | REG | 0.08 | <0.01 | 0.10 | <0.01 |
| 15 | 38473955 | SPRED1 | 0.1314 | REG | −0.05 | <0.01 | −0.07 | <0.01 |
| 15 | 38589695 | SPRED1 | 0.0152 | REG | −0.41 | <0.01 | −0.55 | <0.01 |
| 15 | 38594351 | SPRED1 | 0.0033 | REG | −0.24 | <0.01 | −0.32 | <0.01 |
| 15 | 38633293 | SPRED1 | 0.0033 | REG | 0.26 | <0.01 | 0.35 | <0.01 |
| 15 | 38636745 | SPRED1 | 0.0016 | REG | −0.25 | <0.01 | −0.33 | <0.01 |
| 15 | 38643456 | SPRED1 | 0.0033 | N-S | 0.00 | 0.00 | 0.00 | 0.00 |
| 15 | 38744783 | SPRED1 | 0.0082 | REG | 0.32 | <0.01 | 0.42 | <0.01 |
| 15 | 41049503 | GCHFR | 0.3733 | REG | 0.07 | <0.01 | 0.00 | 0.00 |
| 15 | 41054148 | GCHFR | 0.3682 | REG | 0.06 | <0.01 | 0.00 | 0.00 |
| 15 | 41057507 | GCHFR | 0.0569 | REG | −0.10 | <0.01 | 0.00 | 0.00 |
| 15 | 41525733 | CHP | 0.0235 | REG | −0.28 | <0.01 | 0.00 | 0.00 |
| 15 | 41530395 | CHP | 0.0066 | REG | −0.26 | <0.01 | 0.00 | 0.00 |
| 15 | 41556395 | CHP | 0.0653 | REG | −0.13 | <0.01 | 0.00 | 0.00 |
| 15 | 41564908 | CHP | 0.0033 | REG | 0.47 | <0.01 | 0.00 | 0.00 |
| 15 | 52132166 | TMOD3 | 0.0016 | REG | 0.00 | 0.00 | −0.50 | <0.01 |
| 15 | 52135112 | TMOD3 | 0.0033 | REG | 0.00 | 0.00 | 0.28 | <0.01 |
| 15 | 52166523 | TMOD3 | 0.0033 | REG | 0.00 | 0.00 | −0.67 | <0.01 |
| 15 | 52196830 | TMOD3 | 0.0147 | REG | 0.00 | 0.00 | 0.26 | <0.01 |
| 15 | 52212687 | TMOD3 | 0.0016 | REG | 0.00 | 0.00 | −0.58 | <0.01 |
| 15 | 52214331 | TMOD3 | 0.0017 | REG | 0.00 | 0.00 | −0.34 | <0.01 |
| 15 | 64453924 | PPIB | 0.0203 | REG | 0.00 | 0.00 | 0.25 | <0.01 |
| 15 | 66653196 | MAP2K1 | 0.0016 | REG | −0.56 | <0.01 | −0.48 | <0.01 |
| 15 | 66745125 | MAP2K1 | 0.0083 | REG | −0.29 | <0.01 | −0.25 | <0.01 |
| 15 | 66746483 | MAP2K1 | 0.0049 | REG | −0.38 | <0.01 | −0.33 | <0.01 |
| 15 | 66768655 | MAP2K1 | 0.0082 | REG | −0.31 | <0.01 | −0.26 | <0.01 |
| 15 | 67490186 | AAGAB | 0.0050 | REG | 0.57 | 0.01 | 0.00 | 0.00 |
| 15 | 67494143 | AAGAB | 0.0033 | REG | −0.31 | <0.01 | 0.00 | 0.00 |
| 15 | 67526499 | AAGAB | 0.3691 | REG | 0.19 | 0.02 | 0.00 | 0.00 |
| 15 | 67528374 | AAGAB | 0.4254 | N-S | 0.00 | 0.00 | 0.00 | 0.00 |
| 15 | 70137011 | TLE3 | 0.0033 | REG | 0.25 | <0.01 | 0.00 | 0.00 |
| 15 | 70342654 | TLE3 | 0.0050 | REG | −0.26 | <0.01 | 0.00 | 0.00 |
| 15 | 70350119 | TLE3 | 0.0066 | REG | −0.21 | <0.01 | 0.00 | 0.00 |
| 15 | 70354980 | TLE3 | 0.0033 | REG | −0.39 | <0.01 | 0.00 | 0.00 |
| 15 | 70356226 | TLE3 | 0.0049 | REG | 0.27 | <0.01 | 0.00 | 0.00 |
| 15 | 70375591 | TLE3 | 0.0082 | REG | 0.17 | <0.01 | 0.00 | 0.00 |
| 15 | 70383572 | TLE3 | 0.0016 | REG | 0.67 | <0.01 | 0.00 | 0.00 |
| 15 | 70386386 | TLE3 | 0.0033 | REG | 0.43 | <0.01 | 0.00 | 0.00 |
| 15 | 70399844 | TLE3 | 0.0151 | REG | 0.18 | <0.01 | 0.00 | 0.00 |
| 15 | 70480929 | TLE3 | 0.0801 | REG | −0.10 | <0.01 | 0.00 | 0.00 |
| 15 | 70508450 | TLE3 | 0.0016 | REG | 0.31 | <0.01 | 0.00 | 0.00 |
| 15 | 72978552 | BBS4 | 0.0357 | REG | 0.31 | 0.01 | 0.21 | <0.01 |
| 15 | 72982526 | BBS4 | 0.0033 | REG | −0.62 | <0.01 | −0.43 | <0.01 |
| 15 | 72984698 | BBS4 | 0.0033 | REG | −0.61 | <0.01 | −0.42 | <0.01 |
| 15 | 72993861 | BBS4 | 0.0098 | REG | 0.58 | <0.01 | 0.41 | <0.01 |
| 15 | 72995086 | BBS4 | 0.0116 | REG | −0.45 | <0.01 | −0.31 | <0.01 |
| 15 | 73002101 | BBS4 | 0.0149 | N-S | 0.00 | 0.00 | 0.00 | 0.00 |
| 15 | 73003575 | BBS4 | 0.0065 | REG | −0.57 | <0.01 | −0.39 | <0.01 |
| 15 | 73005266 | BBS4 | 0.0049 | REG | −0.69 | <0.01 | −0.48 | <0.01 |
| 15 | 73027478 | BBS4 | 0.4973 | N-S | 0.00 | 0.00 | 0.00 | 0.00 |
| 15 | 73028152 | BBS4 | 0.0066 | REG | −0.38 | 0.01 | −0.27 | <0.01 |
| 15 | 75022456 | CYP1A2 | 0.0049 | REG | 1.43 | 0.02 | 0.00 | 0.00 |
| 15 | 75040082 | CYP1A2 | 0.0065 | REG | 0.47 | <0.01 | 0.00 | 0.00 |
| 15 | 75041652 | CYP1A2 | 0.0049 | REG | −0.79 | <0.01 | 0.00 | 0.00 |
| 15 | 75043592 | CYP1A2 | 0.0033 | N-S | 0.00 | 0.00 | 0.00 | 0.00 |
| 15 | 75045739 | CYP1A2 | 0.0033 | REG | −0.67 | <0.01 | 0.00 | 0.00 |
| 15 | 75047169 | CYP1A2 | 0.0049 | N-S | 0.96 | <0.01 | 0.00 | 0.00 |
| 15 | 75047412 | CYP1A2 | 0.0017 | N-S | 0.93 | 0.02 | 0.00 | 0.00 |
| 15 | 75752292 | PTPN9 | 0.0067 | REG | −0.43 | <0.01 | 0.00 | 0.00 |
| 15 | 75766087 | PTPN9 | 0.0065 | N-S | 0.00 | 0.00 | 0.00 | 0.00 |
| 15 | 75784903 | PTPN9 | 0.0182 | REG | 0.35 | 0.01 | 0.00 | 0.00 |
| 15 | 75816632 | PTPN9 | 0.0033 | N-S | 0.92 | 0.01 | 0.00 | 0.00 |
| 15 | 75825749 | PTPN9 | 0.0099 | REG | 0.31 | <0.01 | 0.00 | 0.00 |
| 15 | 75829306 | PTPN9 | 0.0065 | REG | 0.94 | 0.01 | 0.00 | 0.00 |
| 15 | 75838027 | PTPN9 | 0.0248 | REG | −0.35 | 0.01 | 0.00 | 0.00 |
| 15 | 79155049 | MORF4L1 | 0.0016 | REG | 0.00 | 0.00 | 0.41 | <0.01 |
| 15 | 79155585 | MORF4L1 | 0.0099 | REG | 0.00 | 0.00 | 0.39 | <0.01 |
| 15 | 79167176 | MORF4L1 | 0.0049 | REG | 0.00 | 0.00 | 0.49 | <0.01 |
| 15 | 79172596 | MORF4L1 | 0.0033 | REG | 0.00 | 0.00 | −0.97 | <0.01 |
| 15 | 79186241 | MORF4L1 | 0.0033 | REG | 0.00 | 0.00 | 0.52 | <0.01 |
| 15 | 79190243 | MORF4L1 | 0.0098 | REG | 0.00 | 0.00 | 0.51 | <0.01 |
| 15 | 79193434 | MORF4L1 | 0.0098 | REG | 0.00 | 0.00 | 0.42 | <0.01 |
| 15 | 89010955 | MRPS11 | 0.0114 | N-S | 0.00 | 0.00 | 0.00 | 0.00 |
| 15 | 89010976 | MRPS11 | 0.0134 | N-S | 0.00 | 0.00 | 0.00 | 0.00 |
| 15 | 89011006 | MRPS11 | 0.0081 | N-S | 0.00 | 0.00 | 0.00 | 0.00 |
| 15 | 89011226 | MRPS11 | 0.0117 | N-S | 0.00 | 0.00 | 0.00 | 0.00 |
| 15 | 89013144 | MRPS11 | 0.0131 | REG | −0.53 | 0.01 | −0.28 | <0.01 |
| 15 | 89019448 | MRPS11 | 0.0083 | REG | −0.35 | <0.01 | −0.19 | <0.01 |
| 15 | 89042462 | MRPS11 | 0.0099 | REG | 0.38 | 0.01 | 0.20 | <0.01 |
| 15 | 89046031 | MRPS11 | 0.0065 | REG | 0.65 | <0.01 | 0.35 | <0.01 |
| 15 | 90437301 | AP3S2 | 0.3954 | REG | −0.07 | <0.01 | −0.09 | <0.01 |
| 17 | 1670370 | SERPINF1 | 0.0016 | REG | 0.75 | <0.01 | 0.71 | <0.01 |
| 17 | 1673276 | SERPINF1 | 0.4821 | N-S | 0.02 | <0.01 | 0.02 | <0.01 |
| 17 | 1674434 | SERPINF1 | 0.0115 | N-S | 0.62 | 0.01 | 0.81 | 0.01 |
| 17 | 1677738 | SERPINF1 | 0.0082 | REG | 0.40 | <0.01 | 0.38 | <0.01 |
| 17 | 1678456 | SERPINF1 | 0.0016 | N-S | 0.00 | 0.00 | 0.00 | 0.00 |
| 17 | 1680558 | SERPINF1 | 0.0033 | N-S | 0.80 | 0.01 | 1.04 | <0.01 |
| 17 | 3582954 | P2RX5 | 0.0050 | N-S | 0.05 | <0.01 | 0.00 | 0.00 |
| 17 | 3583022 | P2RX5 | 0.0016 | N-S | 0.00 | 0.00 | 0.00 | 0.00 |
| 17 | 3599205 | P2RX5 | 0.0170 | N-S | 0.59 | 0.02 | 0.00 | 0.00 |
| 17 | 3921012 | ZZEF1 | 0.0049 | N-S | 0.10 | <0.01 | 0.00 | 0.00 |
| 17 | 3924566 | ZZEF1 | 0.0049 | N-S | 0.00 | 0.00 | 0.00 | 0.00 |
| 17 | 3926110 | ZZEF1 | 0.1587 | N-S | 0.00 | 0.00 | 0.00 | 0.00 |
| 17 | 3947533 | ZZEF1 | 0.1001 | N-S | 0.00 | 0.00 | 0.00 | 0.00 |
| 17 | 3947644 | ZZEF1 | 0.3799 | N-S | 0.00 | 0.00 | 0.00 | 0.00 |
| 17 | 3953102 | ZZEF1 | 0.4729 | N-S | 0.00 | 0.00 | 0.00 | 0.00 |
| 17 | 3955287 | ZZEF1 | 0.0033 | N-S | 0.00 | 0.00 | 0.00 | 0.00 |
| 17 | 3968064 | ZZEF1 | 0.1479 | N-S | 0.00 | 0.00 | 0.00 | 0.00 |
| 17 | 3979935 | ZZEF1 | 0.0033 | N-S | 0.00 | 0.00 | 0.00 | 0.00 |
| 17 | 3980212 | ZZEF1 | 0.0049 | N-S | 0.00 | 0.00 | 0.00 | 0.00 |
| 17 | 3981290 | ZZEF1 | 0.1559 | N-S | 0.00 | 0.00 | 0.00 | 0.00 |
| 17 | 4015917 | ZZEF1 | 0.0016 | N-S | 0.42 | <0.01 | 0.00 | 0.00 |
| 17 | 4046101 | ZZEF1 | 0.3316 | N-S | 0.00 | 0.00 | 0.00 | 0.00 |
| 17 | 4066833 | ANKFY1 | 0.3514 | REG | 0.33 | 0.06 | 0.12 | <0.01 |
| 17 | 4068553 | ANKFY1 | 0.0049 | REG | −1.26 | 0.01 | −0.47 | <0.01 |
| 17 | 4114292 | ANKFY1 | 0.1202 | REG | −0.25 | 0.01 | −0.09 | <0.01 |
| 17 | 4127963 | ANKFY1 | 0.0676 | REG | 0.20 | <0.01 | 0.07 | <0.01 |
| 17 | 4165741 | ANKFY1 | 0.3050 | REG | −0.23 | 0.03 | −0.09 | <0.01 |
| 17 | 4804377 | CHRNE | 0.0033 | N-S | 0.61 | <0.01 | 0.77 | <0.01 |
| 17 | 4806052 | CHRNE | 0.2327 | N-S | 0.05 | <0.01 | 0.06 | <0.01 |
| 17 | 4809038 | CHRNE | 0.0050 | REG | 0.41 | 0.01 | 0.38 | <0.01 |
| 17 | 4810724 | CHRNE | 0.0384 | REG | 0.19 | <0.01 | 0.17 | <0.01 |
| 17 | 5196801 | RABEP1 | 0.0049 | REG | −0.23 | <0.01 | −0.24 | <0.01 |
| 17 | 5199494 | RABEP1 | 0.0328 | REG | −0.08 | <0.01 | −0.08 | <0.01 |
| 17 | 5251654 | RABEP1 | 0.0016 | REG | −0.25 | <0.01 | −0.25 | <0.01 |
| 17 | 5255454 | RABEP1 | 0.1101 | REG | −0.07 | <0.01 | −0.07 | <0.01 |
| 17 | 5270414 | RABEP1 | 0.4614 | REG | 0.10 | 0.01 | 0.10 | <0.01 |
| 17 | 5271763 | RABEP1 | 0.0213 | N-S | −0.33 | 0.01 | −0.47 | 0.01 |
| 17 | 5277598 | RABEP1 | 0.4633 | REG | 0.05 | <0.01 | 0.05 | <0.01 |
| 17 | 5282397 | RABEP1 | 0.0130 | REG | 0.15 | <0.01 | 0.15 | <0.01 |
| 17 | 5284770 | RABEP1 | 0.4962 | REG | −0.09 | 0.01 | −0.09 | <0.01 |
| 17 | 5337039 | C1QBP | 0.0049 | N-S | 0.00 | 0.00 | 0.00 | 0.00 |
| 17 | 5338027 | C1QBP | 0.0033 | REG | 0.64 | <0.01 | 0.82 | <0.01 |
| 17 | 5338269 | C1QBP | 0.0016 | N-S | 0.80 | <0.01 | 1.41 | 0.01 |
| 17 | 5338281 | C1QBP | 0.0098 | N-S | 0.78 | 0.01 | 1.38 | 0.02 |
| 17 | 7123446 | ACADVL | 0.0182 | N-S | −0.04 | <0.01 | −0.09 | <0.01 |
| 17 | 7123506 | ACADVL | 0.0560 | N-S | −0.01 | <0.01 | −0.03 | <0.01 |
| 17 | 7123677 | ACADVL | 0.0033 | REG | 0.39 | <0.01 | 0.60 | <0.01 |
| 17 | 7123838 | ACADVL | 0.0098 | N-S | 0.00 | 0.00 | 0.00 | 0.00 |
| 17 | 7124115 | ACADVL | 0.0049 | N-S | 0.00 | 0.00 | 0.00 | 0.00 |
| 17 | 7124506 | ACADVL | 0.0197 | REG | −0.15 | <0.01 | −0.23 | <0.01 |
| 17 | 7390539 | POLR2A | 0.0016 | REG | 0.00 | 0.00 | −0.41 | <0.01 |
| 17 | 7391761 | POLR2A | 0.0016 | REG | 0.00 | 0.00 | −0.41 | <0.01 |
| 17 | 7404021 | POLR2A | 0.0033 | N-S | 0.00 | 0.00 | 0.25 | <0.01 |
| 17 | 7418780 | POLR2A | 0.0016 | REG | 0.00 | 0.00 | 0.55 | <0.01 |
| 17 | 7448109 | POLR2A | 0.1170 | REG | 0.00 | 0.00 | 0.14 | <0.01 |
| 17 | 7524780 | SAT2 | 0.0016 | REG | 0.50 | <0.01 | 1.17 | 0.01 |
| 17 | 7525548 | SAT2 | 0.2661 | REG | 0.04 | <0.01 | 0.10 | <0.01 |
| 17 | 7529902 | SAT2 | 0.0434 | N-S | 0.34 | 0.01 | 1.11 | 0.04 |
| 17 | 7530892 | SAT2 | 0.0033 | N-S | 0.30 | <0.01 | 0.95 | <0.01 |
| 17 | 7531182 | SAT2 | 0.0082 | REG | −0.29 | <0.01 | −0.67 | <0.01 |
| 17 | 8278956 | RPL26 | 0.1078 | REG | −0.21 | 0.01 | −0.10 | <0.01 |
| 17 | 17409560 | PEMT | 0.4484 | N-S | 0.00 | 0.00 | 0.00 | 0.00 |
| 17 | 17425631 | PEMT | 0.2703 | N-S | 0.00 | 0.00 | 0.00 | 0.00 |
| 17 | 17448024 | PEMT | 0.0016 | REG | 2.07 | 0.02 | 2.33 | 0.01 |
| 17 | 17480319 | PEMT | 0.0049 | N-S | 0.00 | 0.00 | 0.00 | 0.00 |
| 17 | 17498492 | RAI1 | 0.1753 | REG | −0.11 | <0.01 | −0.05 | <0.01 |
| 17 | 17499340 | RAI1 | 0.0082 | REG | −0.46 | <0.01 | −0.22 | <0.01 |
| 17 | 17696531 | RAI1 | 0.4730 | N-S | 0.00 | 0.00 | 0.00 | 0.00 |
| 17 | 17696755 | RAI1 | 0.4870 | N-S | 0.75 | 0.30 | 0.50 | 0.06 |
| 17 | 17697404 | RAI1 | 0.0098 | N-S | 0.11 | <0.01 | 0.07 | <0.01 |
| 17 | 17698990 | RAI1 | 0.0033 | N-S | 0.00 | 0.00 | 0.00 | 0.00 |
| 17 | 17700053 | RAI1 | 0.0033 | N-S | 0.67 | 0.01 | 0.45 | <0.01 |
| 17 | 28574849 | BLMH | 0.4335 | REG | 0.15 | 0.01 | 0.12 | <0.01 |
| 17 | 28576076 | BLMH | 0.3442 | N-S | 0.00 | 0.00 | 0.00 | 0.00 |
| 17 | 28598356 | BLMH | 0.0033 | N-S | −0.41 | <0.01 | −0.44 | <0.01 |
| 17 | 36906480 | PSMB3 | 0.1572 | REG | −0.16 | 0.01 | −0.17 | <0.01 |
| 17 | 36909499 | PSMB3 | 0.3395 | N-S | 0.00 | 0.00 | 0.00 | 0.00 |
| 17 | 37357506 | RPL19 | 0.0017 | N-S | 0.00 | 0.00 | 0.00 | 0.00 |
| 17 | 37365647 | RPL19 | 0.0313 | REG | −0.39 | 0.01 | −0.43 | 0.01 |
| 17 | 38220891 | THRA | 0.0033 | REG | −0.64 | 0.01 | −0.62 | <0.01 |
| 17 | 38244641 | THRA | 0.0098 | REG | 0.27 | <0.01 | 0.26 | <0.01 |
| 17 | 38246286 | THRA | 0.1818 | REG | 0.21 | 0.01 | 0.20 | 0.01 |
| 17 | 38246960 | THRA | 0.0115 | REG | 0.32 | <0.01 | 0.31 | <0.01 |
| 17 | 38342056 | RAPGEFL1 | 0.0033 | REG | 0.47 | <0.01 | 0.41 | <0.01 |
| 17 | 38371750 | RAPGEFL1 | 0.0082 | REG | −0.54 | <0.01 | −0.48 | <0.01 |
| 17 | 38597453 | IGFBP4 | 0.0049 | REG | 0.39 | <0.01 | 0.41 | <0.01 |
| 17 | 38607707 | IGFBP4 | 0.0049 | REG | 0.45 | <0.01 | 0.48 | <0.01 |
| 17 | 38628545 | IGFBP4 | 0.0485 | REG | 0.18 | <0.01 | 0.19 | <0.01 |
| 17 | 38629215 | IGFBP4 | 0.0418 | REG | 0.16 | <0.01 | 0.17 | <0.01 |
| 17 | 38960574 | KRT10 | 0.0033 | REG | −0.89 | 0.01 | 0.00 | 0.00 |
| 17 | 38961122 | KRT10 | 0.2224 | REG | 0.12 | 0.01 | 0.00 | 0.00 |
| 17 | 38978462 | KRT10 | 0.1563 | N-S | 0.06 | <0.01 | 0.00 | 0.00 |
| 17 | 39077408 | KRT23 | 0.0115 | REG | 0.00 | 0.00 | −0.38 | <0.01 |
| 17 | 39083219 | KRT23 | 0.0049 | REG | 0.00 | 0.00 | −0.55 | <0.01 |
| 17 | 39084504 | KRT23 | 0.3293 | N-S | 0.00 | 0.00 | 0.00 | 0.00 |
| 17 | 39092735 | KRT23 | 0.0100 | N-S | 0.00 | 0.00 | 0.00 | 0.00 |
| 17 | 39092756 | KRT23 | 0.1707 | N-S | 0.00 | 0.00 | −0.31 | 0.01 |
| 17 | 39666003 | KRT15 | 0.0475 | REG | −0.16 | <0.01 | 0.00 | 0.00 |
| 17 | 39671724 | KRT15 | 0.0895 | N-S | 0.00 | 0.00 | 0.00 | 0.00 |
| 17 | 39671833 | KRT15 | 0.0033 | N-S | 0.31 | <0.01 | 0.00 | 0.00 |
| 17 | 39672903 | KRT15 | 0.0082 | REG | −0.35 | <0.01 | 0.00 | 0.00 |
| 17 | 39674641 | KRT15 | 0.2036 | N-S | 0.00 | 0.00 | 0.00 | 0.00 |
| 17 | 39675037 | KRT15 | 0.0033 | N-S | 0.03 | <0.01 | 0.00 | 0.00 |
| 17 | 39678128 | KRT15 | 0.0033 | REG | 0.30 | <0.01 | 0.00 | 0.00 |
| 17 | 40313238 | KCNH4 | 0.0033 | REG | 0.63 | <0.01 | 0.66 | <0.01 |
| 17 | 40327123 | KCNH4 | 0.0066 | REG | −1.32 | 0.03 | −1.38 | 0.01 |
| 17 | 41524943 | DHX8 | 0.0034 | REG | 0.48 | <0.01 | 0.43 | <0.01 |
| 17 | 41584589 | DHX8 | 0.0016 | REG | 0.55 | <0.01 | 0.49 | <0.01 |
| 17 | 41590692 | DHX8 | 0.0033 | REG | 0.60 | <0.01 | 0.54 | <0.01 |
| 17 | 41599830 | DHX8 | 0.0201 | REG | −0.27 | <0.01 | −0.24 | <0.01 |
| 17 | 44960539 | GOSR2 | 0.3474 | REG | −0.10 | 0.01 | −0.13 | <0.01 |
| 17 | 45000565 | GOSR2 | 0.0098 | N-S | 0.00 | 0.00 | 0.00 | 0.00 |
| 17 | 45007207 | GOSR2 | 0.1355 | REG | 0.13 | 0.01 | 0.16 | <0.01 |
| 17 | 45008570 | GOSR2 | 0.3118 | N-S | 0.00 | 0.00 | 0.00 | 0.00 |
| 17 | 45010052 | GOSR2 | 0.0016 | REG | 0.60 | <0.01 | 0.77 | <0.01 |
| 17 | 45023456 | GOSR2 | 0.0033 | REG | 1.28 | <0.01 | 1.63 | <0.01 |
| 17 | 45052184 | GOSR2 | 0.0910 | REG | 0.18 | 0.01 | 0.23 | 0.01 |
| 17 | 45053525 | GOSR2 | 0.1915 | REG | −0.13 | 0.01 | −0.16 | <0.01 |
| 17 | 45702026 | KPNB1 | 0.0033 | REG | 0.27 | <0.01 | 0.28 | <0.01 |
| 17 | 45702884 | KPNB1 | 0.0352 | REG | 0.12 | <0.01 | 0.13 | <0.01 |
| 17 | 45735441 | KPNB1 | 0.0033 | REG | 0.20 | <0.01 | 0.21 | <0.01 |
| 17 | 45742931 | KPNB1 | 0.0197 | REG | 0.21 | <0.01 | 0.23 | <0.01 |
| 17 | 45931230 | SP6 | 0.0016 | REG | 0.27 | <0.01 | 0.41 | <0.01 |
| 17 | 45941095 | SP6 | 0.0033 | REG | 0.24 | <0.01 | 0.36 | <0.01 |
| 17 | 45946759 | SP6 | 0.0066 | REG | −0.18 | <0.01 | −0.27 | <0.01 |
| 17 | 45957891 | SP6 | 0.0033 | REG | −0.33 | <0.01 | −0.49 | <0.01 |
| 17 | 48046307 | DLX4 | 0.0739 | REG | 0.30 | 0.02 | 0.25 | 0.01 |
| 17 | 48046963 | DLX4 | 0.0468 | N-S | 0.00 | 0.00 | 0.00 | 0.00 |
| 17 | 48447918 | MRPL27 | 0.0166 | N-S | 0.00 | 0.00 | 0.00 | 0.00 |
| 17 | 49238605 | NME2 | 0.0034 | N-S | 0.80 | 0.01 | 0.98 | 0.01 |
| 17 | 54920944 | DGKE | 0.2043 | REG | 0.13 | 0.01 | 0.00 | 0.00 |
| 17 | 54946968 | DGKE | 0.0049 | REG | −0.63 | <0.01 | 0.00 | 0.00 |
| 17 | 54947550 | DGKE | 0.0065 | REG | 0.78 | <0.01 | 0.00 | 0.00 |
| 17 | 54959254 | DGKE | 0.0116 | REG | 0.34 | <0.01 | 0.00 | 0.00 |
| 17 | 54959504 | DGKE | 0.0049 | REG | 0.44 | <0.01 | 0.00 | 0.00 |
| 17 | 57894459 | VMP1 | 0.3462 | REG | 0.00 | 0.00 | 0.13 | <0.01 |
| 17 | 57914885 | VMP1 | 0.0049 | REG | 0.00 | 0.00 | 1.13 | <0.01 |
| 17 | 61919643 | SMARCD2 | 0.2019 | REG | −0.08 | <0.01 | 0.00 | 0.00 |
| 17 | 61920497 | SMARCD2 | 0.4508 | REG | 0.06 | <0.01 | 0.00 | 0.00 |
| 17 | 66042639 | KPNA2 | 0.0053 | N-S | 0.23 | <0.01 | 0.00 | 0.00 |
| 17 | 68166781 | KCNJ2 | 0.0117 | REG | 0.00 | 0.00 | −0.21 | <0.01 |
| 17 | 68169005 | KCNJ2 | 0.2408 | REG | 0.00 | 0.00 | 0.19 | 0.01 |
| 17 | 68170749 | KCNJ2 | 0.1730 | REG | 0.00 | 0.00 | −0.12 | <0.01 |
| 17 | 69116870 | KCNJ2 | 0.0116 | REG | 0.00 | 0.00 | 0.19 | <0.01 |
| 17 | 69168226 | KCNJ2 | 0.0033 | REG | 0.00 | 0.00 | 0.52 | <0.01 |
| 17 | 71276900 | CDC42EP4 | 0.0199 | REG | 0.16 | <0.01 | 0.00 | 0.00 |
| 17 | 71282336 | CDC42EP4 | 0.0082 | N-S | 0.00 | 0.00 | 0.00 | 0.00 |
| 17 | 71287617 | CDC42EP4 | 0.0218 | REG | −0.13 | <0.01 | 0.00 | 0.00 |
| 17 | 71301361 | CDC42EP4 | 0.0016 | REG | −0.41 | <0.01 | 0.00 | 0.00 |
| 17 | 71301638 | CDC42EP4 | 0.0265 | REG | 0.19 | <0.01 | 0.00 | 0.00 |
| 17 | 71306363 | CDC42EP4 | 0.0033 | REG | −0.34 | <0.01 | 0.00 | 0.00 |
| 17 | 71312262 | CDC42EP4 | 0.0049 | REG | 0.28 | <0.01 | 0.00 | 0.00 |
| 17 | 71328630 | CDC42EP4 | 0.0131 | REG | 0.15 | <0.01 | 0.00 | 0.00 |
| 17 | 71328946 | CDC42EP4 | 0.0568 | REG | −0.08 | <0.01 | 0.00 | 0.00 |
| 17 | 72915919 | USH1G | 0.0049 | N-S | 0.01 | <0.01 | 0.00 | 0.00 |
| 17 | 72916507 | USH1G | 0.0082 | N-S | 0.00 | 0.00 | 0.00 | 0.00 |
| 17 | 72916543 | USH1G | 0.0082 | N-S | 0.33 | <0.01 | 0.00 | 0.00 |
| 17 | 79011383 | BAIAP2 | 0.0230 | REG | −0.37 | 0.01 | 0.00 | 0.00 |
| 17 | 79014782 | BAIAP2 | 0.0033 | REG | −0.58 | <0.01 | 0.00 | 0.00 |
| 17 | 79021982 | BAIAP2 | 0.0050 | REG | −1.09 | 0.02 | 0.00 | 0.00 |
| 17 | 79031160 | BAIAP2 | 0.0081 | REG | 0.58 | <0.01 | 0.00 | 0.00 |
| 17 | 79036463 | BAIAP2 | 0.0016 | REG | 0.62 | <0.01 | 0.00 | 0.00 |
| 17 | 79041209 | BAIAP2 | 0.2567 | REG | 0.15 | 0.01 | 0.00 | 0.00 |
| 17 | 79043205 | BAIAP2 | 0.0052 | REG | −1.89 | 0.06 | 0.00 | 0.00 |
| 17 | 79060247 | BAIAP2 | 0.0033 | N-S | 0.00 | 0.00 | 0.00 | 0.00 |
| 17 | 79068735 | BAIAP2 | 0.0033 | REG | −0.70 | <0.01 | 0.00 | 0.00 |
| 17 | 79071441 | BAIAP2 | 0.3604 | REG | 0.12 | 0.01 | 0.00 | 0.00 |
| 17 | 79080586 | BAIAP2 | 0.0114 | N-S | 0.00 | 0.00 | 0.00 | 0.00 |
| 17 | 79089590 | BAIAP2 | 0.3821 | N-S | 0.00 | 0.00 | 0.00 | 0.00 |
| 17 | 79089679 | BAIAP2 | 0.0016 | REG | −0.86 | 0.01 | 0.00 | 0.00 |
| 18 | 19378049 | MIB1 | 0.0016 | N-S | −0.21 | <0.01 | 0.00 | 0.00 |
| 19 | 521715 | CDC34 | 0.1455 | REG | 0.08 | <0.01 | 0.06 | <0.01 |
| 19 | 522436 | CDC34 | 0.3988 | REG | −0.07 | <0.01 | −0.05 | <0.01 |
| 19 | 526969 | CDC34 | 0.0049 | REG | −0.47 | <0.01 | −0.35 | <0.01 |
| 19 | 527751 | CDC34 | 0.0016 | REG | 0.48 | <0.01 | 0.36 | <0.01 |
| 19 | 534062 | CDC34 | 0.1324 | REG | −0.13 | <0.01 | −0.09 | <0.01 |
| 19 | 535248 | CDC34 | 0.0131 | REG | −0.26 | <0.01 | −0.19 | <0.01 |
| 19 | 1239398 | ATP5D | 0.1590 | REG | −0.06 | <0.01 | −0.07 | <0.01 |
| 19 | 1242915 | ATP5D | 0.0081 | REG | 0.21 | <0.01 | 0.22 | <0.01 |
| 19 | 1243372 | ATP5D | 0.0131 | REG | 0.16 | <0.01 | 0.17 | <0.01 |
| 19 | 1246549 | ATP5D | 0.0164 | REG | 0.20 | <0.01 | 0.22 | <0.01 |
| 19 | 1247144 | ATP5D | 0.4817 | REG | −0.05 | <0.01 | −0.05 | <0.01 |
| 19 | 1448180 | RPS15 | 0.3516 | REG | 0.08 | <0.01 | 0.00 | 0.00 |
| 19 | 5914605 | CAPS | 0.0583 | N-S | 0.00 | 0.00 | 0.00 | 0.00 |
| 19 | 5915008 | CAPS | 0.0033 | N-S | −0.51 | <0.01 | −0.56 | <0.01 |
| 19 | 5915438 | CAPS | 0.0065 | REG | 0.38 | <0.01 | 0.31 | <0.01 |
| 19 | 5915883 | CAPS | 0.3279 | REG | −0.08 | <0.01 | −0.06 | <0.01 |
| 19 | 6222353 | MLLT1 | 0.0265 | N-S | 0.01 | <0.01 | 0.02 | <0.01 |
| 19 | 6224181 | MLLT1 | 0.0049 | REG | −0.33 | <0.01 | −0.27 | <0.01 |
| 19 | 6261777 | MLLT1 | 0.0196 | REG | −0.15 | <0.01 | −0.12 | <0.01 |
| 19 | 9490760 | ZNF177 | 0.0217 | N-S | −0.74 | 0.03 | −0.69 | 0.01 |
| 19 | 9490814 | ZNF177 | 0.4790 | N-S | −0.01 | <0.01 | −0.01 | <0.01 |
| 19 | 9492071 | ZNF177 | 0.0049 | N-S | −0.86 | 0.01 | −0.80 | <0.01 |
| 19 | 9492370 | ZNF177 | 0.4444 | N-S | 0.00 | 0.00 | 0.00 | 0.00 |
| 19 | 9754397 | ZNF562 | 0.0417 | REG | −0.24 | <0.01 | 0.00 | 0.00 |
| 19 | 9757697 | ZNF562 | 0.0084 | REG | −0.30 | <0.01 | 0.00 | 0.00 |
| 19 | 9764117 | ZNF562 | 0.0049 | N-S | −0.04 | <0.01 | 0.00 | 0.00 |
| 19 | 9764192 | ZNF562 | 0.0016 | N-S | 0.00 | 0.00 | 0.00 | 0.00 |
| 19 | 9781436 | ZNF562 | 0.0065 | REG | 0.43 | <0.01 | 0.00 | 0.00 |
| 19 | 9782940 | ZNF562 | 0.1332 | REG | −0.11 | <0.01 | 0.00 | 0.00 |
| 19 | 9788298 | ZNF562 | 0.0099 | REG | 0.31 | <0.01 | 0.00 | 0.00 |
| 19 | 9788619 | ZNF562 | 0.0082 | REG | 0.48 | <0.01 | 0.00 | 0.00 |
| 19 | 10071347 | COL5A3 | 0.2223 | N-S | 0.03 | <0.01 | 0.06 | <0.01 |
| 19 | 10077419 | COL5A3 | 0.3279 | N-S | 0.00 | 0.00 | 0.00 | 0.00 |
| 19 | 10079093 | COL5A3 | 0.1587 | N-S | 0.07 | <0.01 | 0.12 | <0.01 |
| 19 | 10084292 | COL5A3 | 0.2977 | N-S | 0.00 | 0.00 | 0.00 | 0.00 |
| 19 | 10084460 | COL5A3 | 0.0167 | N-S | 0.26 | <0.01 | 0.44 | <0.01 |
| 19 | 10085054 | COL5A3 | 0.1448 | N-S | 0.32 | 0.03 | 0.54 | 0.04 |
| 19 | 10085062 | COL5A3 | 0.1349 | N-S | 0.35 | 0.03 | 0.59 | 0.04 |
| 19 | 10088127 | COL5A3 | 0.0033 | N-S | 0.37 | <0.01 | 0.62 | <0.01 |
| 19 | 10088271 | COL5A3 | 0.2304 | N-S | 0.00 | 0.00 | 0.00 | 0.00 |
| 19 | 10097073 | COL5A3 | 0.0049 | N-S | 0.11 | <0.01 | 0.19 | <0.01 |
| 19 | 10103700 | COL5A3 | 0.0033 | N-S | 0.14 | <0.01 | 0.23 | <0.01 |
| 19 | 10104342 | COL5A3 | 0.0049 | N-S | 0.17 | <0.01 | 0.29 | <0.01 |
| 19 | 10112346 | COL5A3 | 0.1680 | N-S | 0.01 | <0.01 | 0.02 | <0.01 |
| 19 | 10114740 | COL5A3 | 0.1188 | N-S | 0.03 | <0.01 | 0.04 | <0.01 |
| 19 | 10116508 | COL5A3 | 0.0618 | N-S | 0.01 | <0.01 | 0.01 | <0.01 |
| 19 | 10117024 | COL5A3 | 0.4350 | REG | 0.09 | 0.01 | 0.11 | <0.01 |
| 19 | 10117055 | COL5A3 | 0.2969 | REG | −0.11 | 0.01 | −0.13 | <0.01 |
| 19 | 12513424 | ZNF443 | 0.0114 | REG | 0.46 | 0.01 | 0.39 | <0.01 |
| 19 | 12516909 | ZNF443 | 0.0082 | REG | −1.26 | 0.01 | −1.08 | 0.01 |
| 19 | 12541191 | ZNF443 | 0.0016 | N-S | −0.56 | <0.01 | −0.66 | <0.01 |
| 19 | 12541214 | ZNF443 | 0.3652 | N-S | 0.00 | 0.00 | 0.00 | 0.00 |
| 19 | 12541250 | ZNF443 | 0.0305 | N-S | −0.58 | 0.03 | −0.69 | 0.02 |
| 19 | 12541497 | ZNF443 | 0.0049 | N-S | 0.00 | 0.00 | 0.00 | 0.00 |
| 19 | 12541532 | ZNF443 | 0.2299 | N-S | 0.00 | 0.00 | 0.00 | 0.00 |
| 19 | 12541547 | ZNF443 | 0.4150 | N-S | −0.20 | 0.02 | −0.23 | 0.02 |
| 19 | 12541795 | ZNF443 | 0.3624 | N-S | −0.65 | 0.26 | −0.77 | 0.17 |
| 19 | 12541811 | ZNF443 | 0.0033 | N-S | 0.00 | 0.00 | 0.00 | 0.00 |
| 19 | 12543974 | ZNF443 | 0.0082 | N-S | −0.60 | 0.01 | −0.71 | <0.01 |
| 19 | 12551028 | ZNF443 | 0.2788 | REG | −0.09 | <0.01 | −0.08 | <0.01 |
| 19 | 12552029 | ZNF443 | 0.0065 | REG | 1.12 | 0.01 | 0.96 | <0.01 |
| 19 | 14263184 | LPHN1 | 0.0016 | N-S | 0.23 | <0.01 | 0.29 | <0.01 |
| 19 | 14269279 | LPHN1 | 0.0131 | N-S | 0.00 | 0.00 | 0.00 | 0.00 |
| 19 | 14286489 | LPHN1 | 0.0033 | REG | 0.90 | 0.01 | 0.85 | <0.01 |
| 19 | 14290238 | LPHN1 | 0.2763 | REG | 0.10 | <0.01 | 0.09 | <0.01 |
| 19 | 14297207 | LPHN1 | 0.0766 | REG | −0.13 | <0.01 | −0.12 | <0.01 |
| 19 | 14297942 | LPHN1 | 0.0016 | REG | 0.40 | <0.01 | 0.38 | <0.01 |
| 19 | 14306357 | LPHN1 | 0.0320 | REG | 0.25 | <0.01 | 0.23 | <0.01 |
| 19 | 14306739 | LPHN1 | 0.0065 | REG | 0.57 | <0.01 | 0.54 | <0.01 |
| 19 | 14459314 | LPHN1 | 0.1128 | REG | −0.16 | 0.01 | −0.15 | <0.01 |
| 19 | 14487877 | LPHN1 | 0.3870 | REG | 0.07 | <0.01 | 0.07 | <0.01 |
| 19 | 14677580 | NDUFB7 | 0.0281 | N-S | 0.00 | 0.00 | 0.00 | 0.00 |
| 19 | 14725723 | EMR3 | 0.0033 | REG | 0.23 | <0.01 | 0.39 | <0.01 |
| 19 | 14728673 | EMR3 | 0.2939 | REG | −0.11 | 0.01 | −0.19 | 0.01 |
| 19 | 14730547 | EMR3 | 0.0148 | REG | −0.21 | <0.01 | −0.35 | <0.01 |
| 19 | 14736316 | EMR3 | 0.1436 | REG | −0.06 | <0.01 | −0.09 | <0.01 |
| 19 | 14737514 | EMR3 | 0.0033 | REG | 0.33 | <0.01 | 0.56 | <0.01 |
| 19 | 14747767 | EMR3 | 0.0016 | REG | −0.57 | <0.01 | −0.95 | <0.01 |
| 19 | 14749131 | EMR3 | 0.0016 | N-S | 0.10 | <0.01 | 0.22 | <0.01 |
| 19 | 14752325 | EMR3 | 0.0789 | N-S | 0.00 | 0.00 | 0.00 | 0.00 |
| 19 | 14752344 | EMR3 | 0.0065 | N-S | 0.09 | <0.01 | 0.20 | <0.01 |
| 19 | 14755209 | EMR3 | 0.0049 | REG | 0.50 | <0.01 | 0.83 | <0.01 |
| 19 | 14758168 | EMR3 | 0.2046 | N-S | 0.00 | 0.00 | 0.00 | 0.00 |
| 19 | 14758650 | EMR3 | 0.0033 | REG | 0.52 | <0.01 | 0.87 | <0.01 |
| 19 | 14769339 | EMR3 | 0.4530 | N-S | 0.00 | 0.00 | 0.00 | 0.00 |
| 19 | 14773659 | EMR3 | 0.0033 | REG | 0.34 | <0.01 | 0.57 | <0.01 |
| 19 | 14776476 | EMR3 | 0.0098 | REG | −0.24 | <0.01 | −0.40 | <0.01 |
| 19 | 14785226 | EMR3 | 0.0134 | REG | 0.25 | <0.01 | 0.42 | <0.01 |
| 19 | 16994090 | F2RL3 | 0.3731 | REG | 0.05 | <0.01 | 0.00 | 0.00 |
| 19 | 17000632 | F2RL3 | 0.2457 | N-S | 0.00 | 0.00 | 0.00 | 0.00 |
| 19 | 17001203 | F2RL3 | 0.0033 | N-S | 0.48 | <0.01 | 0.00 | 0.00 |
| 19 | 19017862 | COPE | 0.1140 | N-S | 0.00 | 0.00 | 0.00 | 0.00 |
| 19 | 19019800 | COPE | 0.0608 | REG | −0.16 | <0.01 | −0.25 | <0.01 |
| 19 | 19023812 | COPE | 0.0016 | N-S | 0.51 | <0.01 | 1.12 | 0.01 |
| 19 | 19026862 | COPE | 0.0016 | REG | 0.25 | <0.01 | 0.39 | <0.01 |
| 19 | 19030120 | COPE | 0.0099 | N-S | 0.11 | <0.01 | 0.25 | <0.01 |
| 19 | 33671172 | LRP3 | 0.3860 | REG | −0.11 | 0.01 | 0.00 | 0.00 |
| 19 | 33682977 | LRP3 | 0.0033 | REG | 0.38 | <0.01 | 0.00 | 0.00 |
| 19 | 33683053 | LRP3 | 0.0065 | REG | 0.45 | <0.01 | 0.00 | 0.00 |
| 19 | 33693784 | LRP3 | 0.0049 | N-S | 0.58 | 0.01 | 0.00 | 0.00 |
| 19 | 33697048 | LRP3 | 0.0050 | N-S | 0.52 | <0.01 | 0.00 | 0.00 |
| 19 | 33697380 | LRP3 | 0.0320 | REG | −0.13 | <0.01 | 0.00 | 0.00 |
| 19 | 33698291 | LRP3 | 0.2825 | N-S | 0.00 | 0.00 | 0.00 | 0.00 |
| 19 | 33698426 | LRP3 | 0.0016 | N-S | 0.00 | 0.00 | 0.00 | 0.00 |
| 19 | 33698476 | LRP3 | 0.0049 | N-S | 0.55 | <0.01 | 0.00 | 0.00 |
| 19 | 33699535 | LRP3 | 0.2936 | REG | 0.09 | <0.01 | 0.00 | 0.00 |
| 19 | 36211359 | MLL4 | 0.0049 | REG | 0.00 | 0.00 | 0.95 | <0.01 |
| 19 | 36600743 | POLR2I | 0.2868 | REG | 0.10 | <0.01 | 0.06 | <0.01 |
| 19 | 36603703 | POLR2I | 0.4065 | REG | 0.13 | 0.01 | 0.08 | <0.01 |
| 19 | 36645231 | ZNF565 | 0.0016 | REG | −0.48 | <0.01 | −0.59 | <0.01 |
| 19 | 36645683 | ZNF565 | 0.0065 | REG | −0.81 | 0.01 | −0.99 | <0.01 |
| 19 | 36674305 | ZNF565 | 0.3686 | N-S | 0.00 | 0.00 | 0.00 | 0.00 |
| 19 | 36678220 | ZNF565 | 0.0051 | REG | 0.46 | <0.01 | 0.56 | <0.01 |
| 19 | 36681135 | ZNF565 | 0.0049 | REG | −0.57 | <0.01 | −0.70 | <0.01 |
| 19 | 36684491 | ZNF565 | 0.3828 | REG | 0.07 | <0.01 | 0.08 | <0.01 |
| 19 | 36684504 | ZNF565 | 0.0016 | REG | −0.54 | <0.01 | −0.67 | <0.01 |
| 19 | 36703207 | ZNF565 | 0.0017 | REG | 0.25 | <0.01 | 0.31 | <0.01 |
| 19 | 39225690 | CAPN12 | 0.0049 | REG | −0.50 | <0.01 | −0.68 | <0.01 |
| 19 | 39227253 | CAPN12 | 0.0732 | REG | −0.19 | 0.01 | −0.26 | 0.01 |
| 19 | 39228244 | CAPN12 | 0.1054 | N-S | 0.30 | 0.02 | 0.56 | 0.03 |
| 19 | 39229089 | CAPN12 | 0.1129 | N-S | 0.00 | 0.00 | 0.00 | 0.00 |
| 19 | 39230046 | CAPN12 | 0.2536 | REG | 0.13 | 0.01 | 0.18 | 0.01 |
| 19 | 39230852 | CAPN12 | 0.0349 | N-S | 0.41 | 0.01 | 0.77 | 0.02 |
| 19 | 39234511 | CAPN12 | 0.1087 | REG | −0.14 | <0.01 | −0.19 | <0.01 |
| 19 | 39256965 | CAPN12 | 0.0049 | REG | 0.49 | <0.01 | 0.67 | <0.01 |
| 19 | 39260000 | CAPN12 | 0.4213 | REG | 0.08 | <0.01 | 0.10 | <0.01 |
| 19 | 39260282 | CAPN12 | 0.3117 | REG | −0.10 | 0.01 | −0.13 | <0.01 |
| 19 | 40325254 | FBL | 0.0049 | REG | −0.28 | <0.01 | −0.58 | <0.01 |
| 19 | 40325564 | FBL | 0.0147 | REG | 0.28 | <0.01 | 0.57 | <0.01 |
| 19 | 40330874 | FBL | 0.0033 | N-S | 0.00 | 0.00 | 0.00 | 0.00 |
| 19 | 40331352 | FBL | 0.0017 | N-S | 0.24 | <0.01 | 0.68 | 0.01 |
| 19 | 40340611 | FBL | 0.0548 | REG | 0.13 | <0.01 | 0.27 | <0.01 |
| 19 | 40978640 | SPTBN4 | 0.0869 | N-S | 0.00 | 0.00 | −0.01 | <0.01 |
| 19 | 41038574 | SPTBN4 | 0.2815 | N-S | 0.00 | 0.00 | 0.00 | 0.00 |
| 19 | 41056229 | SPTBN4 | 0.0327 | N-S | −0.59 | 0.01 | −1.11 | 0.02 |
| 19 | 41073863 | SPTBN4 | 0.0904 | N-S | −0.06 | <0.01 | −0.12 | <0.01 |
| 19 | 41074162 | SPTBN4 | 0.0115 | N-S | 0.00 | 0.00 | 0.00 | 0.00 |
| 19 | 41259576 | SNRPA | 0.0410 | REG | −0.15 | <0.01 | −0.18 | <0.01 |
| 19 | 41263403 | SNRPA | 0.0478 | REG | −0.20 | <0.01 | −0.25 | <0.01 |
| 19 | 42574183 | ZNF574 | 0.0065 | REG | −0.31 | <0.01 | −0.27 | <0.01 |
| 19 | 42583644 | ZNF574 | 0.0066 | N-S | 0.00 | 0.00 | 0.00 | 0.00 |
| 19 | 42584518 | ZNF574 | 0.0049 | N-S | 0.22 | <0.01 | 0.27 | <0.01 |
| 19 | 42584890 | ZNF574 | 0.0082 | N-S | 0.00 | 0.00 | 0.00 | 0.00 |
| 19 | 42584938 | ZNF574 | 0.0016 | N-S | 0.00 | 0.00 | 0.00 | 0.00 |
| 19 | 43430638 | PSG7 | 0.0049 | REG | 0.86 | <0.01 | 0.84 | <0.01 |
| 19 | 43459724 | PSG7 | 0.0149 | REG | 0.32 | <0.01 | 0.31 | <0.01 |
| 19 | 43464801 | PSG7 | 0.0033 | REG | −1.06 | 0.01 | −1.04 | <0.01 |
| 19 | 43472497 | PSG7 | 0.0033 | REG | 0.81 | <0.01 | 0.80 | <0.01 |
| 19 | 44035399 | ZNF575 | 0.0065 | REG | −0.67 | 0.01 | −0.58 | <0.01 |
| 19 | 44040582 | ZNF575 | 0.0335 | REG | −0.27 | 0.01 | −0.23 | <0.01 |
| 19 | 44673059 | ZNF226 | 0.0166 | REG | −0.30 | <0.01 | 0.00 | 0.00 |
| 19 | 44677156 | ZNF226 | 0.0287 | REG | −0.39 | 0.01 | 0.00 | 0.00 |
| 19 | 44714922 | ZNF226 | 0.0168 | REG | 0.17 | <0.01 | 0.00 | 0.00 |
| 19 | 44954931 | ZNF180 | 0.0033 | REG | 0.44 | <0.01 | 0.37 | <0.01 |
| 19 | 44956058 | ZNF180 | 0.2453 | REG | −0.06 | <0.01 | −0.05 | <0.01 |
| 19 | 44979910 | ZNF180 | 0.0082 | REG | 0.57 | 0.01 | 0.48 | <0.01 |
| 19 | 44981079 | ZNF180 | 0.0017 | N-S | −0.45 | <0.01 | −0.51 | <0.01 |
| 19 | 44981800 | ZNF180 | 0.0082 | N-S | 0.00 | 0.00 | 0.00 | 0.00 |
| 19 | 44981832 | ZNF180 | 0.0115 | N-S | −0.04 | <0.01 | −0.04 | <0.01 |
| 19 | 44981862 | ZNF180 | 0.0033 | N-S | −0.03 | <0.01 | −0.03 | <0.01 |
| 19 | 44981883 | ZNF180 | 0.3337 | N-S | 0.00 | 0.00 | 0.00 | 0.00 |
| 19 | 44983567 | ZNF180 | 0.3417 | N-S | −0.40 | 0.08 | −0.46 | 0.05 |
| 19 | 44988567 | ZNF180 | 0.0033 | REG | 0.32 | <0.01 | 0.26 | <0.01 |
| 19 | 44988639 | ZNF180 | 0.0033 | N-S | 0.00 | 0.00 | 0.00 | 0.00 |
| 19 | 44991346 | ZNF180 | 0.0082 | REG | −0.57 | <0.01 | −0.47 | <0.01 |
| 19 | 45001346 | ZNF180 | 0.1841 | N-S | 0.00 | 0.00 | 0.00 | 0.00 |
| 19 | 45513615 | RELB | 0.2553 | REG | −0.04 | <0.01 | 0.00 | 0.00 |
| 19 | 45531163 | RELB | 0.0066 | REG | −0.25 | <0.01 | 0.00 | 0.00 |
| 19 | 45537823 | RELB | 0.0033 | REG | 0.64 | <0.01 | 0.00 | 0.00 |
| 19 | 45541414 | RELB | 0.0444 | REG | −0.08 | <0.01 | 0.00 | 0.00 |
| 19 | 45944237 | FOSB | 0.0033 | REG | −0.42 | <0.01 | 0.00 | 0.00 |
| 19 | 45955273 | FOSB | 0.1647 | REG | 0.11 | <0.01 | 0.00 | 0.00 |
| 19 | 45964964 | FOSB | 0.0033 | REG | 0.34 | <0.01 | 0.00 | 0.00 |
| 19 | 45966114 | FOSB | 0.0066 | REG | 0.28 | <0.01 | 0.00 | 0.00 |
| 19 | 45986373 | FOSB | 0.0016 | REG | 0.39 | <0.01 | 0.00 | 0.00 |
| 19 | 46009596 | VASP | 0.0016 | REG | −0.34 | <0.01 | −0.44 | <0.01 |
| 19 | 46011609 | VASP | 0.0049 | REG | −0.54 | <0.01 | −0.69 | <0.01 |
| 19 | 46268902 | SIX5 | 0.2380 | N-S | 0.00 | 0.00 | 0.00 | 0.00 |
| 19 | 46269076 | SIX5 | 0.3784 | N-S | 0.38 | 0.08 | 0.00 | 0.00 |
| 19 | 46269313 | SIX5 | 0.1978 | N-S | 0.00 | 0.00 | 0.00 | 0.00 |
| 19 | 46735860 | HIF3A | 0.0033 | REG | 1.91 | 0.03 | 1.33 | 0.01 |
| 19 | 46736440 | HIF3A | 0.0437 | REG | −0.36 | 0.01 | −0.25 | <0.01 |
| 19 | 46736926 | HIF3A | 0.0165 | REG | 0.86 | 0.02 | 0.60 | 0.01 |
| 19 | 46795807 | HIF3A | 0.0049 | REG | 1.18 | 0.03 | 0.82 | 0.01 |
| 19 | 46812451 | HIF3A | 0.0369 | N-S | 0.99 | 0.09 | 0.95 | 0.04 |
| 19 | 46823702 | HIF3A | 0.3217 | N-S | 0.00 | 0.00 | 0.00 | 0.00 |
| 19 | 46823803 | HIF3A | 0.0033 | N-S | 0.00 | 0.00 | 0.00 | 0.00 |
| 19 | 46828843 | HIF3A | 0.0033 | N-S | 0.00 | 0.00 | 0.00 | 0.00 |
| 19 | 48827882 | EMP3 | 0.0393 | REG | 0.00 | 0.00 | 0.28 | <0.01 |
| 19 | 48833608 | EMP3 | 0.0306 | N-S | 0.00 | 0.00 | −0.65 | 0.02 |
| 19 | 48834349 | EMP3 | 0.0082 | REG | 0.00 | 0.00 | −0.75 | <0.01 |
| 19 | 48834553 | EMP3 | 0.0066 | REG | 0.00 | 0.00 | −0.40 | <0.01 |
| 19 | 49997571 | RPS11 | 0.1086 | REG | 0.19 | 0.01 | 0.00 | 0.00 |
| 19 | 50031075 | RCN3 | 0.0758 | REG | 0.26 | 0.01 | 0.35 | 0.01 |
| 19 | 50040353 | RCN3 | 0.0065 | N-S | 0.54 | <0.01 | 0.99 | <0.01 |
| 19 | 50045878 | RCN3 | 0.0417 | N-S | 0.52 | 0.02 | 0.96 | 0.03 |
| 19 | 50045979 | RCN3 | 0.0131 | N-S | 0.00 | 0.00 | 0.00 | 0.00 |
| 19 | 51644501 | SIGLEC7 | 0.1307 | REG | −0.08 | <0.01 | −0.06 | <0.01 |
| 19 | 51646140 | SIGLEC7 | 0.0366 | REG | −0.17 | <0.01 | −0.12 | <0.01 |
| 19 | 51649123 | SIGLEC7 | 0.0016 | N-S | 0.47 | <0.01 | 0.48 | <0.01 |
| 19 | 51667554 | SIGLEC7 | 0.0229 | REG | 0.24 | <0.01 | 0.18 | <0.01 |
| 19 | 52229176 | FPR1 | 0.0033 | REG | 0.41 | <0.01 | 0.37 | <0.01 |
| 19 | 52249211 | FPR1 | 0.1419 | N-S | −0.22 | 0.01 | −0.28 | 0.01 |
| 19 | 52249680 | FPR1 | 0.1126 | N-S | 0.00 | 0.00 | 0.00 | 0.00 |
| 19 | 52249947 | FPR1 | 0.4209 | N-S | 0.00 | 0.00 | 0.00 | 0.00 |
| 19 | 52250216 | FPR1 | 0.1852 | N-S | 0.00 | 0.00 | 0.00 | 0.00 |
| 19 | 52255196 | FPR1 | 0.2222 | REG | 0.22 | 0.02 | 0.20 | 0.01 |
| 19 | 52258510 | FPR1 | 0.0016 | REG | 0.49 | <0.01 | 0.44 | <0.01 |
| 19 | 52468203 | ZNF350 | 0.1370 | N-S | −0.16 | 0.01 | 0.00 | 0.00 |
| 19 | 52468292 | ZNF350 | 0.0256 | N-S | 0.00 | 0.00 | 0.00 | 0.00 |
| 19 | 52471872 | ZNF350 | 0.1688 | N-S | 0.00 | 0.00 | 0.00 | 0.00 |
| 19 | 52473689 | ZNF350 | 0.0382 | REG | 0.18 | <0.01 | 0.00 | 0.00 |
| 19 | 52492062 | ZNF350 | 0.0065 | REG | −0.28 | <0.01 | 0.00 | 0.00 |
| 19 | 52492352 | ZNF350 | 0.4174 | REG | 0.08 | <0.01 | 0.00 | 0.00 |
| 19 | 55587822 | EPS8L1 | 0.1393 | N-S | 0.00 | 0.00 | 0.00 | 0.00 |
| 19 | 55588936 | EPS8L1 | 0.0033 | REG | 1.05 | 0.01 | 0.85 | <0.01 |
| 19 | 55590594 | EPS8L1 | 0.1798 | REG | 0.14 | 0.01 | 0.11 | <0.01 |
| 19 | 55594996 | EPS8L1 | 0.1523 | N-S | 0.01 | <0.01 | 0.01 | <0.01 |
| 19 | 55598724 | EPS8L1 | 0.4456 | N-S | 0.50 | 0.14 | 0.55 | 0.08 |
| 19 | 55598927 | EPS8L1 | 0.0016 | N-S | 0.00 | 0.00 | 0.00 | 0.00 |
| 19 | 55897327 | RPL28 | 0.1956 | REG | 0.00 | 0.00 | −0.23 | 0.01 |
| 19 | 55901036 | RPL28 | 0.0033 | REG | 0.00 | 0.00 | −1.03 | <0.01 |
| 19 | 55908158 | RPL28 | 0.0016 | REG | 0.00 | 0.00 | −0.46 | <0.01 |
| 19 | 55908199 | RPL28 | 0.2624 | REG | 0.00 | 0.00 | −0.08 | <0.01 |
| 19 | 56061360 | ZNF579 | 0.0848 | REG | 0.09 | <0.01 | 0.00 | 0.00 |
| 19 | 56086762 | ZNF579 | 0.0049 | REG | 0.42 | <0.01 | 0.00 | 0.00 |
| 19 | 56087676 | ZNF579 | 0.0016 | REG | 0.51 | <0.01 | 0.00 | 0.00 |
| 19 | 56091899 | ZNF579 | 0.0751 | REG | −0.09 | <0.01 | 0.00 | 0.00 |
| 19 | 56094778 | ZNF579 | 0.1504 | REG | 0.08 | <0.01 | 0.00 | 0.00 |
| 19 | 57931303 | ZNF17 | 0.2993 | N-S | −0.47 | 0.10 | −0.37 | 0.03 |
| 19 | 57931425 | ZNF17 | 0.0179 | N-S | 0.00 | 0.00 | 0.00 | 0.00 |
| 19 | 57932052 | ZNF17 | 0.0033 | N-S | −0.69 | 0.01 | −0.55 | <0.01 |
| 19 | 57932571 | ZNF17 | 0.0016 | N-S | −0.66 | 0.01 | −0.53 | <0.01 |
| 19 | 58135693 | ZNF211 | 0.0034 | REG | 0.66 | <0.01 | 0.00 | 0.00 |
| 19 | 58144256 | ZNF211 | 0.1251 | REG | −0.08 | <0.01 | 0.00 | 0.00 |
| 19 | 58144418 | ZNF211 | 0.0099 | REG | −0.37 | <0.01 | 0.00 | 0.00 |
| 19 | 58144715 | ZNF211 | 0.1308 | N-S | 0.00 | 0.00 | 0.00 | 0.00 |
| 19 | 58144731 | ZNF211 | 0.0049 | N-S | 0.00 | 0.00 | 0.00 | 0.00 |
| 19 | 58152795 | ZNF211 | 0.1308 | N-S | 0.00 | 0.00 | 0.00 | 0.00 |
| 19 | 58158765 | ZNF211 | 0.0049 | REG | −0.44 | <0.01 | 0.00 | 0.00 |
| 19 | 58175855 | ZNF211 | 0.2158 | REG | 0.05 | <0.01 | 0.00 | 0.00 |
| 19 | 58176227 | ZNF211 | 0.0149 | REG | 0.31 | <0.01 | 0.00 | 0.00 |
| 19 | 58273441 | ZNF586 | 0.1519 | REG | −0.26 | 0.02 | −0.31 | 0.01 |
| 19 | 58277771 | ZNF586 | 0.0081 | REG | 0.36 | <0.01 | 0.43 | <0.01 |
| 19 | 58282878 | ZNF586 | 0.4774 | REG | 0.38 | 0.08 | 0.45 | 0.06 |
| 19 | 58291082 | ZNF586 | 0.0116 | N-S | 0.00 | 0.00 | 0.00 | 0.00 |
| 19 | 58310895 | ZNF586 | 0.4792 | REG | 0.25 | 0.03 | 0.29 | 0.02 |
| 19 | 58316489 | ZNF586 | 0.1486 | REG | 0.18 | 0.01 | 0.22 | 0.01 |
| 19 | 58740339 | ZNF544 | 0.1883 | REG | −0.23 | 0.02 | −0.13 | <0.01 |
| 19 | 58750597 | ZNF544 | 0.0033 | REG | −0.37 | <0.01 | −0.21 | <0.01 |
| 19 | 58772321 | ZNF544 | 0.0049 | N-S | 0.00 | 0.00 | 0.00 | 0.00 |
| 19 | 58772510 | ZNF544 | 0.0033 | N-S | 0.00 | 0.00 | 0.00 | 0.00 |
| 19 | 58772579 | ZNF544 | 0.2150 | N-S | −0.71 | 0.18 | −0.54 | 0.05 |
| 19 | 58773831 | ZNF544 | 0.0049 | N-S | −0.01 | <0.01 | −0.01 | <0.01 |
| 19 | 58774071 | ZNF544 | 0.4440 | N-S | 0.00 | 0.00 | 0.00 | 0.00 |
| 19 | 58774094 | ZNF544 | 0.0049 | N-S | −0.72 | <0.01 | −0.55 | <0.01 |
| 19 | 58785900 | ZNF544 | 0.4235 | REG | 0.10 | 0.01 | 0.06 | <0.01 |
| 19 | 58931557 | ZNF132 | 0.2751 | REG | 0.28 | 0.03 | 0.42 | 0.04 |
| 19 | 58931966 | ZNF132 | 0.0739 | REG | −0.09 | <0.01 | −0.13 | <0.01 |
| 19 | 58939165 | ZNF132 | 0.2937 | REG | −0.22 | 0.02 | −0.33 | 0.02 |
| 19 | 58944778 | ZNF132 | 0.0049 | N-S | −0.09 | <0.01 | −0.19 | <0.01 |
| 19 | 58945948 | ZNF132 | 0.0049 | N-S | −0.06 | <0.01 | −0.12 | <0.01 |
| 19 | 58946056 | ZNF132 | 0.3013 | N-S | 0.00 | 0.00 | 0.00 | 0.00 |
| 19 | 58946203 | ZNF132 | 0.1111 | N-S | 0.00 | 0.00 | 0.00 | 0.00 |
| 19 | 58951647 | ZNF132 | 0.0065 | REG | −0.58 | <0.01 | −0.87 | <0.01 |
| 19 | 58958265 | ZNF132 | 0.0033 | REG | 0.58 | <0.01 | 0.87 | <0.01 |
| 21 | 32253513 | KRTAP11−1 | 0.2422 | N-S | 0.71 | 0.19 | 0.84 | 0.13 |
| 21 | 32253629 | KRTAP11−1 | 0.1120 | N-S | 0.00 | 0.00 | 0.00 | 0.00 |
| 21 | 33780298 | C21orf63 | 0.1265 | REG | −0.07 | <0.01 | −0.09 | <0.01 |
| 21 | 33791873 | C21orf63 | 0.0516 | REG | −0.18 | <0.01 | −0.23 | <0.01 |
| 21 | 33798932 | C21orf63 | 0.0083 | REG | 0.19 | <0.01 | 0.24 | <0.01 |
| 21 | 33801408 | C21orf63 | 0.0459 | REG | −0.48 | 0.03 | −0.61 | 0.02 |
| 21 | 33807279 | C21orf63 | 0.0357 | REG | 0.18 | <0.01 | 0.22 | <0.01 |
| 21 | 33825695 | C21orf63 | 0.0033 | N-S | 0.49 | <0.01 | 0.85 | <0.01 |
| 21 | 33837613 | C21orf63 | 0.0456 | REG | 0.36 | 0.01 | 0.46 | 0.01 |
| 21 | 33838713 | C21orf63 | 0.0474 | REG | 0.37 | 0.01 | 0.47 | 0.01 |
| 21 | 33844624 | C21orf63 | 0.0440 | REG | −0.38 | 0.01 | −0.48 | 0.01 |
| 21 | 33850239 | C21orf63 | 0.0386 | REG | 0.22 | <0.01 | 0.28 | <0.01 |
| 21 | 33850397 | C21orf63 | 0.3731 | REG | −0.07 | <0.01 | −0.09 | <0.01 |
| 21 | 33887315 | C21orf63 | 0.0033 | N-S | 0.00 | 0.00 | 0.00 | 0.00 |
| 21 | 36171613 | RUNX1 | 0.0049 | N-S | 0.00 | 0.00 | 0.00 | 0.00 |
| 21 | 36259324 | RUNX1 | 0.0116 | N-S | −0.03 | <0.01 | −0.03 | <0.01 |
| 21 | 45553106 | C21orf33 | 0.0016 | REG | 0.00 | 0.00 | −0.51 | <0.01 |
| 21 | 45553596 | C21orf33 | 0.3286 | N-S | 0.00 | 0.00 | 0.09 | <0.01 |
| 21 | 45560145 | C21orf33 | 0.0065 | N-S | 0.00 | 0.00 | 0.12 | <0.01 |
| 21 | 45560848 | C21orf33 | 0.0432 | REG | 0.00 | 0.00 | 0.17 | <0.01 |
| 21 | 45564766 | C21orf33 | 0.0986 | N-S | 0.00 | 0.00 | 0.00 | 0.00 |
| 21 | 45566467 | C21orf33 | 0.3759 | REG | 0.00 | 0.00 | −0.10 | <0.01 |
| 21 | 46266768 | PTTG1IP | 0.0428 | REG | −0.98 | 0.10 | −0.76 | 0.03 |
| 21 | 46267290 | PTTG1IP | 0.0411 | REG | 1.24 | 0.16 | 0.95 | 0.04 |
| 21 | 46268783 | PTTG1IP | 0.3481 | REG | −0.10 | 0.01 | −0.08 | <0.01 |
| 21 | 46268897 | PTTG1IP | 0.0065 | REG | −0.63 | <0.01 | −0.48 | <0.01 |
| 21 | 46279169 | PTTG1IP | 0.0033 | REG | 0.46 | <0.01 | 0.35 | <0.01 |
| 21 | 46288005 | PTTG1IP | 0.0098 | REG | −0.53 | <0.01 | −0.41 | <0.01 |
| 21 | 46294986 | PTTG1IP | 0.2598 | REG | −0.10 | 0.01 | −0.08 | <0.01 |
